# Exciton trapping with a twist

**DOI:** 10.1039/d5sc06393k

**Published:** 2025-12-18

**Authors:** Chinju Govind, Israa Shioukhi, Yinon Deree, Jhon Sebastian Oviedo Ortiz, Jeanne Crassous, Ori Gidron, Eric Vauthey

**Affiliations:** a Department of Physical Chemistry, University of Geneva 30 Quai Ernest-Ansermet CH-1211 Geneva 4 Switzerland eric.vauthey@unige.ch; b Institute of Chemistry, The Hebrew University of Jerusalem Givat Ram Jerusalem 9190401 Israel ori.gidron@mail.huji.ac.il; c University of Rennes, CNRS ISCR – UMR 6226 35000 Rennes France

## Abstract

In electron donor–acceptor (D–A) molecules, the relative orientation of constituents has a dramatic influence over their performance. However, the D and A subunits are generally composed of planar aromatic backbones, and the effect of curvature is rarely explored. Here, we investigate how the twist of the aromatic core of a symmetric double-branched D–π–A molecule affects the nature and dynamics of its lower singlet excited state. We show that the twisting of the central donor not only affects the chiroptical properties, and increases the triplet yield, but also facilitates excited-state symmetry breaking (ESSB) and the trapping of the exciton on one D–π–A branch of the molecule. This enhancement is attributed to the decrease in the interbranch coupling upon distortion. Because of this, the loss of the coupling upon ESSB requires a smaller gain in solvation energy to be compensated for and, thus, exciton trapping occurs in a less polar solvent. Consequently, distortion can be viewed as an additional tuning knob for controlling the localisation of electronic excitation in large conjugated systems.

## Abstract 2

Twisting of the central donor unit of a quadrupolar molecule induces chirality, enhances intersystem crossing and facilitates the localisation of the electronic excitation on one side *via* excited-state symmetry breaking.

## Graphical abstract

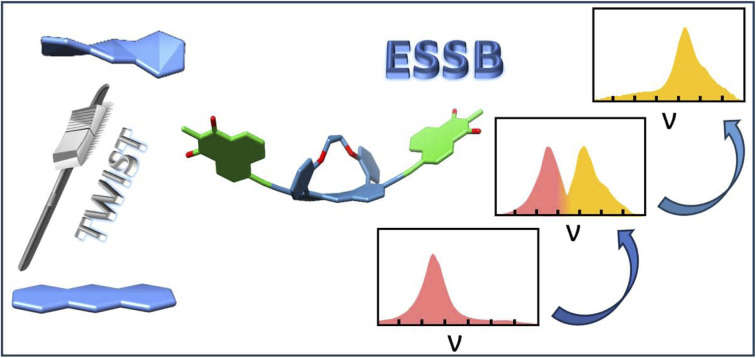

## 1 Introduction

Molecules containing two or more donor–acceptor (D–A) branches serve as active materials in an increasing number of applications such as organic photovoltaics and light-emitting devices.^1–4^ The relative orientation between the donor and acceptor units can affect the nature of the lowest electronic excited state (*e.g.* charge-transfer or locally excited state) and, in turn, the device performance. Over the past few years, there has been a growing interest in exploring how out-of-plane twisting distortion affects the excited-state properties of aromatic molecules. These investigations were mostly focused on the effect of distortion on the intersystem-crossing (ISC) dynamics, the motivation being to achieve high triplet yield without recourse to the heavy-atom effect.^5–7^ They revealed that, although twisting can, in some cases, increase spin–orbit coupling (SOC) and accelerate ISC,^7–13^ this effect is not general.^14–17^ Out-of-plane distortions can also induce chirality and can thus be used to develop materials with circularly polarized luminescence (CPL).^18,19^ CPL emitting materials have potential applications in quantum computing, optical data storage, bioresponsive imaging, 3D displays, and optical spintronics.^20^

**Fig. 1:**
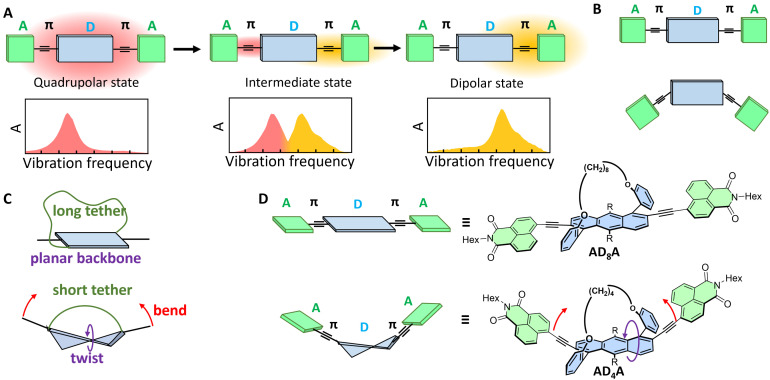
(A) Schematic representation of excited-state symmetry breaking and exciton localisation in A–π–D–π–A molecules together with their effect on the IR absorption spectrum in the –C≡C– stretching region. (B) A–π–D–π–A molecules with centrosymmetric (top) non-centrosymmetric (bottom) orientation. (C) Molecular tethering approach to control the twisting and bending of the donor. (D) Structure of the A–π–D–π–A molecules studied in this work.

Multibranched D–π–A dyes are known for their tendency to undergo excited-state symmetry breaking (ESSB).^21–27^ During this process, the electronic excitation, initially distributed evenly over the whole molecule (quadrupolar state, Fig. 1A), localises, at least partially, on a single branch, conferring a strong dipolar character to the S_1_ state, despite the symmetric molecular structure (dipolar state, Fig. 1A). Such exciton localisation upon ESSB, which often involves an intermediate state with uneven distribution of the excitation (Fig. 1A), was found to depend on multiple parameters, such as the solvent polarity,^24,28^ the electron donating and withdrawing strength of the D and A subunits as well as the branch length,^29,30^ and the relative orientation between donor and acceptor (*e.g.* centrosymmetric *vs.* noncentrosymmetric, Fig. 1B).^31^ However, the influence of out-of-plane distortion on exciton localisation is less understood.

ESSB was recently reported in helical A–π–D–π–A molecules, with a helicene donor.^18^ The trapping of the exciton on one D–π–A branch was identified by the strong decrease of the CPL upon increasing solvent polarity. This work inspired us to quantitatively explore how ESSB is affected by the extent of donor twist. Such quantitative control of the backbone twisting in aromatic molecules can be obtained by the tether-to-distortion approach. Diagonally tethered acenes can be twisted and bent out of plane, depending on the alkyl tether length (Fig. 1C).^32,33^ Such distortions affect their (chiro)optical and electronic properties. In addition, we demonstrated that twisting increases the ISC rate by enhancing the spin–orbit coupling.^11^

Here, we explore how twisting in the aromatic backbone affects the photophysical properties of two A–π–D–π–A molecules, AD_n_A, varying by the amount of distortion. The latter is achieved upon twisting the anthracene donor core *via* an alkyl-chain tether of varying length (*n* = 4 or 8, Fig. 1D). We observe both electronic circular dichroism (ECD) and CPL in particular with the more twisted AD_4_A. Using a combination of ultrafast transient electronic and vibrational absorption spectroscopies, we find that, in addition to having a larger triplet yield, the more twisted molecule, AD_4_A, has a higher propensity to undergo ESSB than the quasi-planar one, AD_8_A. Consequently, molecular distortion can be viewed as a new tuning knob for controlling the spatial distribution of exciton in large conjugated systems.

## 2 Results

### 2.1 Synthesis and structure

**Fig. 2:**
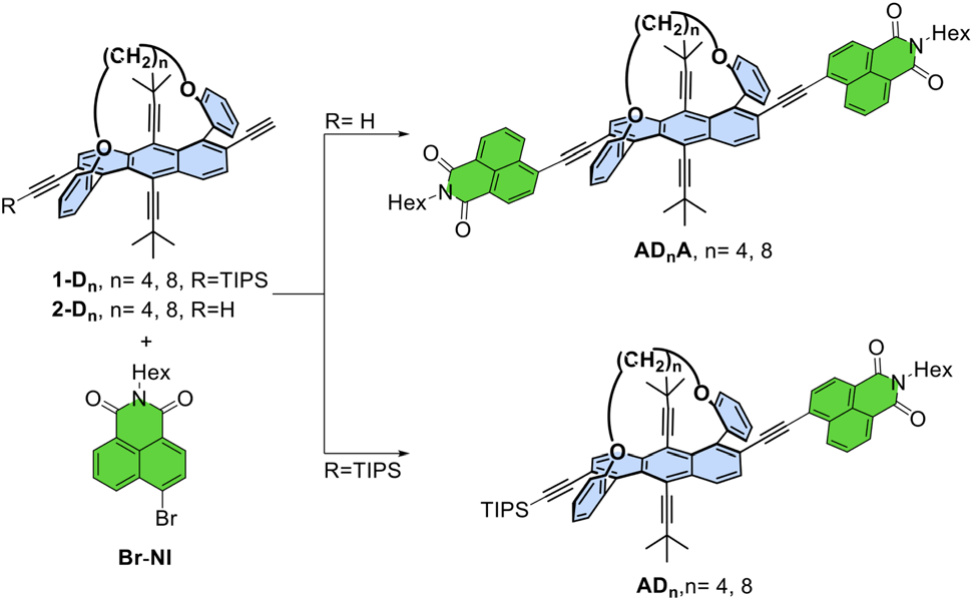
Synthesis of AD_n_ and AD_n_A. Reagents and conditions: Pd(PPh_3_)_4_ 5% mol, CuI 10% mol, NEt_3_, THF, room temperature.

Acceptor–π–donor–π–acceptor AD_n_A molecules were synthesized *via* the Sonogashira coupling reaction between the butyl or octyl-tethered twistacenes, 2-D_4_ and 2-D_8_ respectively, with *N*-hexyl-4-bromo-1,8-napthalimide (Br–NI) as depicted in Fig. 2.^33^AD_n_ was synthesized by coupling 1-D_n_ with Br–NI (Fig. 2). The products were obtained in both racemic and enantiopure forms, starting from either *P*-1-D_n_, *M*-1-D_n_, or racemic 1-D_n_, where the twistacenes exhibit either *P* or *M* helicities. The coupling products were obtained with yields ranging from 30% to 60%, and characterized using NMR spectroscopy and HRMS spectrometry (Fig. S1–S32).

**Fig. 3:**
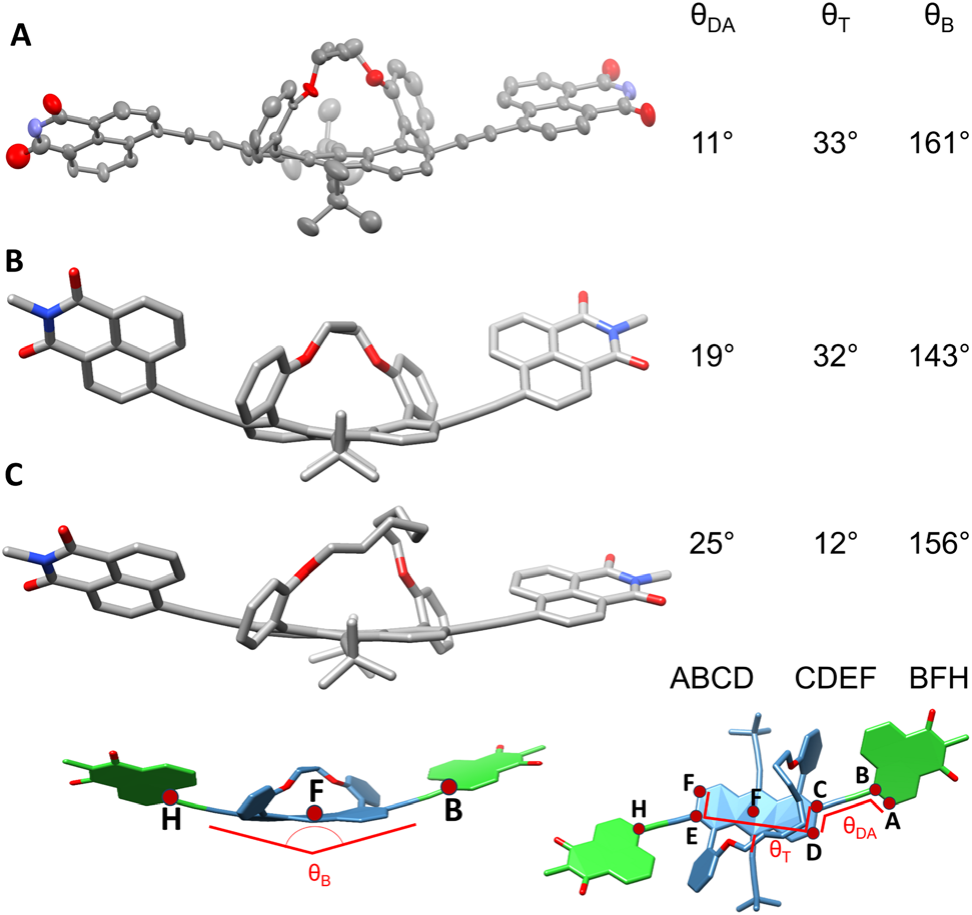
(A) X-ray structure of racemic AD_4_A (ellipsoid representation). (B) Calculated (DFT/B3LYP/6-31G(*d*)-D3) structures of AD_4_A and (C) AD_8_A.

Fig. 3A displays the single crystal X-ray structure of AD_4_A grown by the slow evaporation of hexane and ethyl acetate mixture. The end-to-end twist dihedral angle, expressed as *θ*_T_ in Fig. 3, is 33°, consistent with previously reported values for anthracene cores with butyl tether.^33^AD_4_A is V-shaped, with a bend of *θ*_B_ = 161°. Gas-phase quantum-chemical calculations at the density functional theory (DFT) level with Grimme's dispersion correction (B3LYP/6-31g(d,p)-D3)^34,35^ were carried out to obtain a better insight into the structural distortion brought about by the tethers (see Section S7 in SI for details).

The optimized geometry of AD_4_A exhibits a distortion of *θ*_T_ = 32°, in agreement with the X-ray structure (Fig. 3B). However, the calculated bending angle of 143° predicts a more bent structure. In contrast, the optimized geometry for AD_8_A is more planar, with a smaller twist angle of *θ*_T_ = 12° and a bending angle of 156° (Fig. 3C). As predicted, the end-to-end backbone twist directly affects the bending between the donors and the acceptors.

A 2-dimensional scan around the two rotatable bonds reveals three minima, with the acceptor in either an anti or syn orientation (Fig. S63). However, the most stable conformation, in which the acceptors are slightly rotated with respect to the donor, with a dihedral angle of 25°, is 1.8 and 3.6 kcal mol^−1^ lower than the other conformers (thus over 95% more abundant). This orientation is stabilised by the H⋯π interactions between hydrogen on the electron-deficient naphthalimide and electron-rich *ortho*-anisole. However, it should be noted that these calculations were performed in the gas phase, and therefore the molecules could adopt a different conformation depending on the solvent.

### 2.2 Stationary spectroscopy

#### 2.2.1 Electronic absorption and emission

**Fig. 4:**
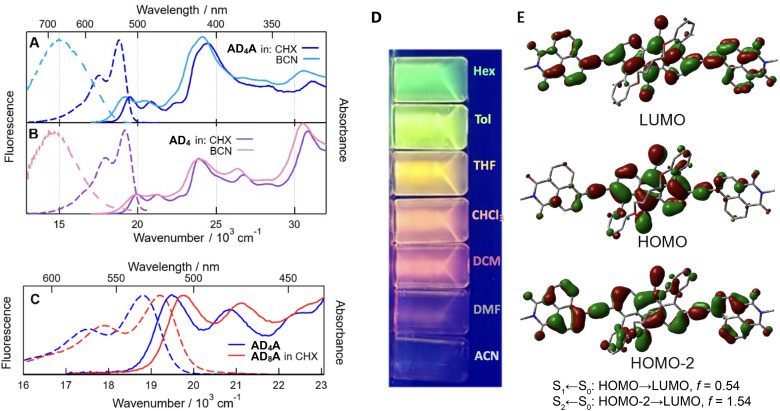
Stationary electronic absorption and emission spectra of AD_4_A (A) and AD_4_ (B) in cyclohexane and benzonitrile and of AD_4_A (C) and AD_8_A in cyclohexane. (D) Emission of AD_4_A in different solvents, irradiated at 365 nm. (E) Frontier molecular orbitals involved in the lowest-energy transitions (S_1_ ← S_0_ and S_2_ ← S_0_) of AD_4_A and associated oscillator strengths, *f*.

AD_n_A dyes exhibit continuous absorption below ∼530 nm due to multiple electronic transitions (Fig. 4A and S33–S35). According to time-dependent (TD) DFT calculations (TD-DFT/CAM-B3LYP/6-31G(d)),^36^ the lowest-energy band with a vibronic structure can be attributed to the S_1_ ← S_0_ transition and is mostly associated with a one-electron HOMO to LUMO transition. As illustrated in Fig. 4E, this transition involves significant charge transfer (CT) from the anthracene core to the naphthalimide ends. This results in a strong quadrupolar character of the S_1_ state of AD_n_A. The more intense absorption band peaking around 410 nm can be assigned to the S_2_ ← S_0_ transition. Calculations also predict a more intense S_2_ ← S_0_ band due predominantly to a one-electron HOMO-2 to LUMO transition, pointing to a negligible CT character. The large oscillator strength of this transition is typical of highly delocalised excitation, as it is also the case for other linear conjugated molecules.^37–39^

The absorption spectra of the single-branch AD_n_ analogues are very similar apart from a 440 cm^−1^ blue shift of the low-energy band and the higher intensity of the bands at 380 and 300 nm (Fig. 4B). This intensity difference is probably due to the reduction of symmetry upon going from AD_n_A to AD_n_ and the lifting of the Laporte rules.^40^ The calculations predict a high density of excited states above S_2_, in agreement with the continuous absorption below ∼530 nm. Because of this, the origin of these 380 and 330 nm bands could not be identified. For both AD_n_A and AD_n_, the lowest-energy band shifts to the red by about 300 cm^−1^ upon shortening the tether (Fig. 4C). This shift is consistent with the decrease of the S_1_ ← S_0_ gap upon increasing distortion predicted by the calculations (Fig. S62).

All four compounds show only a small absorption solvatochromism, which correlates with the refractive index (Fig. S43–S46). This points to a small permanent dipole moment in the ground state and to dispersion as the dominant solute–solvent interactions.

The AD_n_A molecules are highly fluorescent in cyclohexane (CHX) with fluorescence quantum yields ranging from 0.4 to 0.5 (Table S6) and a spectrum that mirrors the lowest-energy absorption band. Strong emission solvatochromism is observed with all dyes (Fig. 4D and S39–S46, Tables S2–S5). The correlation with the orientational polarization function of the solvent, Δ*f*, indicates that it is dominated by dipole–dipole interactions.^41^ The solvatochromic shift observed when going from hexane to acetonitrile (ACN) amounts to about 4000 cm^−1^ for AD_n_A and to ∼5000 cm^−1^ for AD_n_. Whereas a large permanent dipole moment is expected for D–π–A dyads in a CT excited state, it is more surprising for the near-centrosymmetric AD_n_A. Their large fluorescence solvatochromism, demonstrated in Fig. 4D points to the occurrence of ESSB and to a dipolar excited state in a polar environment, with the exciton trapped on a single D–π–A branch. The red shift of the fluorescence upon increasing solvent polarity is accompanied by a broadening of the band and a substantial decrease in quantum yield (Table S6).

#### 2.2.2 Circular dichroism and circular polarised luminescence

**Fig. 5:**
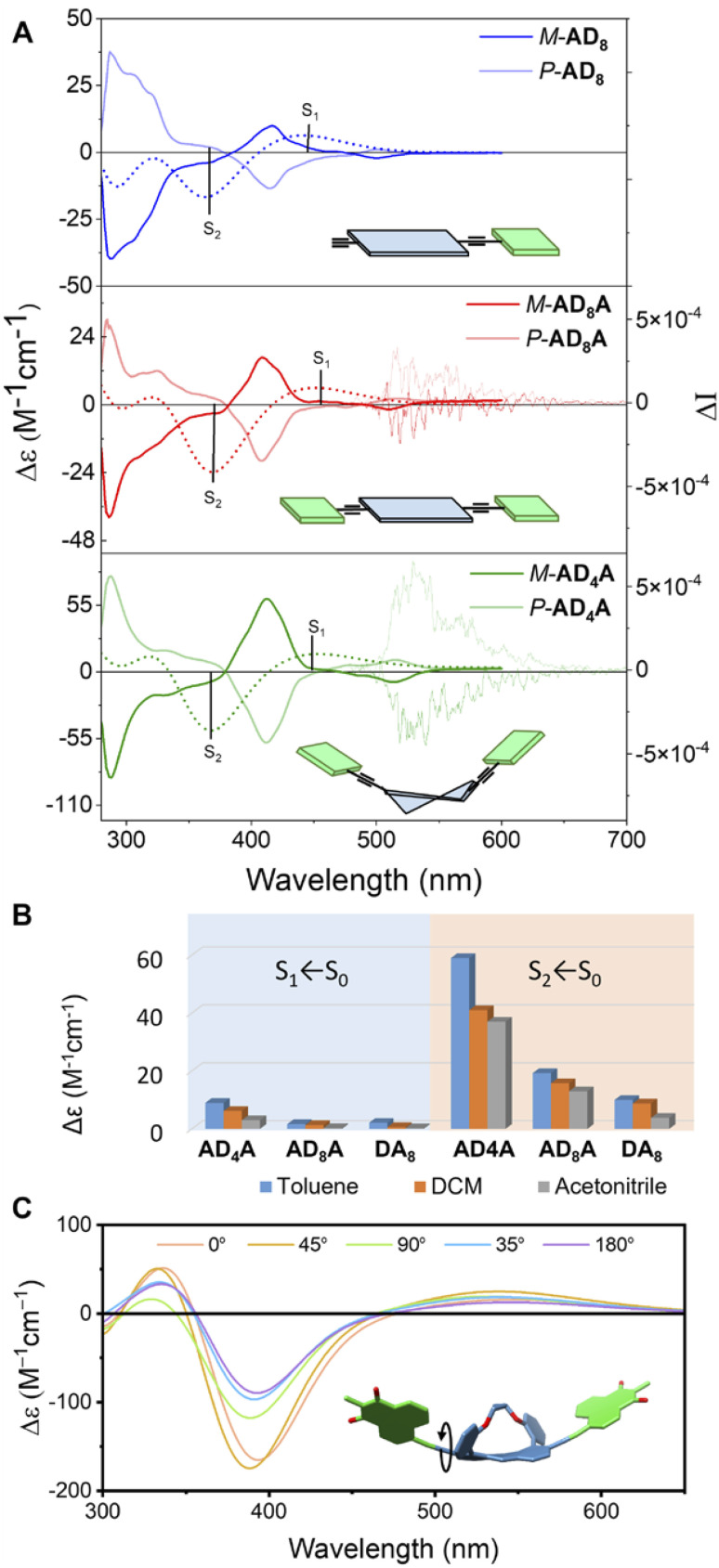
(A) ECD and CPL spectra of AD_8_, AD_8_A and AD_4_A in hexane. Dotted line: calculated spectrum for the *P* enantiomers (TD-DFT/CAM-B3LYP/6-31G(d)). (B) Maximal molar ellipticity of AD_4_A, AD_8_A and AD_8_ in different solvents. (C) Calculated ECD spectra of AD_4_A with different A–D orientations.

Fig. 5A displays the electronic circular dichroism (ECD) and CPL spectra of the *M* and *P* enantiomers of AD_8_, AD_8_A and AD_4_A. The calculated spectra (TD-DFT/CAM-B3LYP/6-31G(d)) for all three compounds match the experimental spectra with a slight energy shift, confirming the absolute configuration of the *M* enantiomers and the assignments of the nature of the transitions as discussed above (Fig. 5A, dotted lines). The ECD spectra display both the above-mentioned S_1_ ← S_0_ and S_2_ ← S_0_ bands. The molar ellipticity of the S_1_ ← S_0_ transition increases upon twisting, from 1.6 M^−1^ cm^−1^ for AD_8_A to 9 M^−1^ cm^−1^ for AD_4_A, whereas for the more intense S_2_ ← S_0_ band, it changes from 19 M^−1^ cm^−1^ for AD_8_A to 60 M^−1^ cm^−1^ for AD_4_A.

We note that compared to the reactant (anthracene with no acceptor, 2-D_n_), the optical activity of the S_2_ ← S_0_ transition is significantly stronger, and of opposite sign relative to that observed for the S_1_ ← S_0_ transition. This can be explained by the delocalisation of this excitation over the whole molecule as suggested by the quantum-chemical calculations (Fig. 4E). The molar ellipticity, particularly that of the S_2_ ← S_0_ transition of AD_4_A varies with the solvent (Fig. 5C and S37), although no clear trend with a solvent property can be identified. TD-DFT calculations of the ECD spectra suggest that this effect could be due to different relative orientations of the D–A subunits, depending on the solvent (Fig. 5C).

Distortion and solvent polarity also impact the CPL activity. Indeed, as can be seen Fig. 5A, S48 and S49 for AD_4_A and AD_8_A in hexane, higher distortion results in a more intense CPL signal and a stronger dissymmetry factor (P-AD_4_A*g*_lum_ = 6 × 10^−4^ at 530 nm; P-AD_8_A*g*_lum_ = 3 × 10^−4^ at 517 nm in hexane). In polar solvents such as DCM, the CPL signal was too weak to be properly measured. Finally, no CPL signal could be clearly detected with the polar AD_n_ systems.

### 2.3 Time-resolved spectroscopy

#### 2.3.1 Transient electronic spectroscopy

**Fig. 6:**
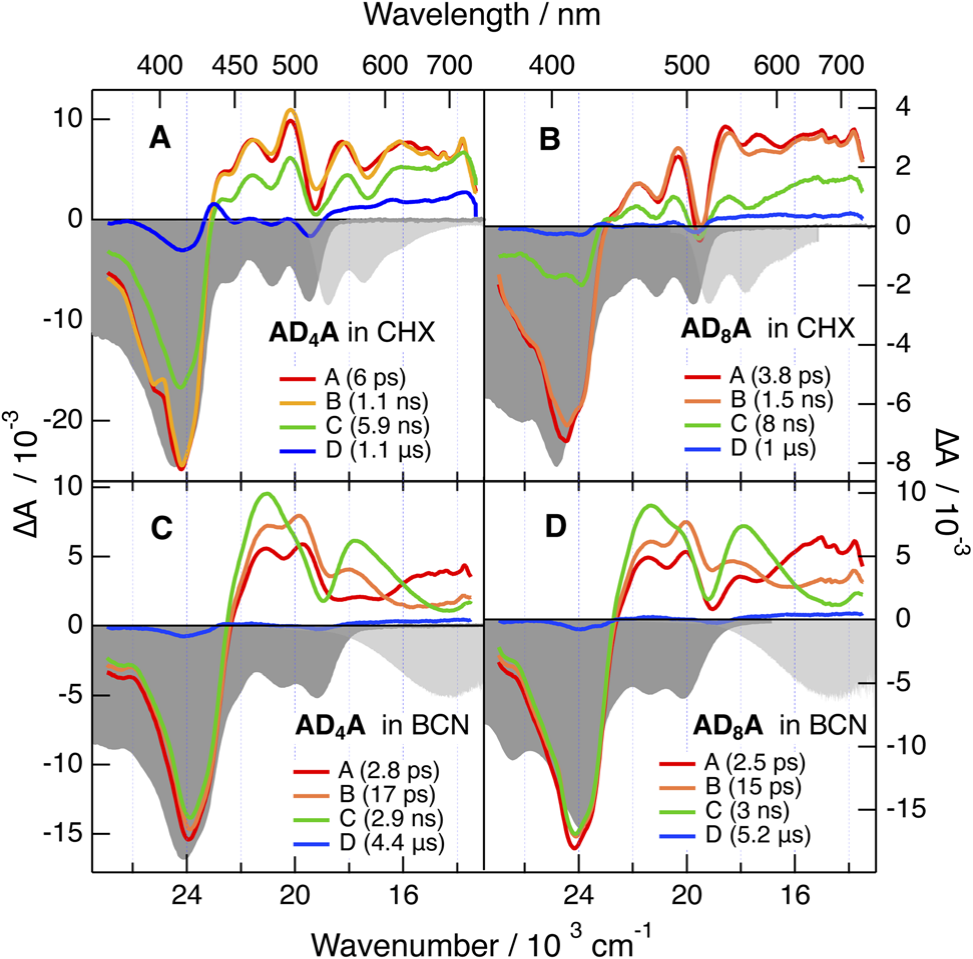
Evolution-associated difference absorption spectra and time constants obtained from a global analysis of the transient electronic absorption spectra recorded after 400 nm excitation of AD_n_A in cyclohexane (A and B) and benzonitrile (C and D), assuming a series of four successive exponential steps (A → B → …). The negative stationary absorption and stimulated emission spectra are shown in gray.

Transient electronic absorption (TA) measurements were performed in the non-polar CHX and in the polar benzonitrile (BCN) up to 100 µs with an approx. 100 fs resolution (Fig. S51 and S52). The data were analysed globally assuming a series of successive exponential steps to obtain evolution-associated difference absorption spectra (EADS) and time constants (Fig. 6 and S53). As illustrated in Fig. 6A, B and S51–S53, the early TA spectra recorded with all dyes in CHX consist of negative bands below 430 nm and positive bands above. The negative bands can be assigned to the ground state bleach (GSB) of higher–energy transitions. The positive bands can be attributed to excited-state absorption (ESA) overlapping with negative bands due to both S_1_ ← S_0_ GSB and S_1_ → S_0_ stimulated emission (SE). The SE band around 515 nm is significantly more intense for AD_n_A than AD_n_. All transient bands decay on the ns timescale to a residual spectrum exhibiting the GSB bands as well as ESA bands above 520 nm and at 430 nm and decaying in a few µs. The ns dynamics after which the SE band is no longer visible can be attributed to the decay of the S_1_ state. The ESA bands in the residual spectrum are assigned to the T_1_ state, in accordance with the 430 nm band reported in the triplet state spectrum of anthracene.^42,43^ This feature is the most intense with AD_4_A and is hardly visible with AD_8_A.

The TA dynamics measured in BCN are very similar for all four molecules (Fig. 6C, D, S51 and S52). The early spectra resemble those in CHX. However, they evolve within about 20 ps into spectra dominated by two ESA features separated by the S_1_ ← S_0_ GSB at 500 nm. Concurrently to these changes, the SE band shifts to the red, broadens and becomes similar to the stationary emission band. Afterwards, these features decay on a few ns timescale to a very weak residual spectrum that itself decays in a few µs.

Given its timescale, the shift of the SE band can be assigned to the solvent relaxation around the CT excited state.^44^ As discussed below, the accompanying transformation of the ESA bands most probably reflects the increase of the CT character of the excited state as solvent relaxation takes place. This significant CT character of the S_1_ state is consistent with the absorption spectra of the radical anion of naphthalimide and of the radical cation of diphenylethynyl-anthracene with bands around 490 nm and in the 400–550 nm region, respectively.^45,46^

The resemblance of the spectra measured with AD_n_A and AD_n_ points to a similar equilibrium S_1_ state, hence to ESSB in AD_n_A and trapping of the excitation on one branch. This interpretation will be corroborated by the time-resolved IR (TRIR) spectroscopic measurements described below. The lifetime of the S_1_ state is markedly shorter than in CHX, namely 2–3 ns *vs.* 6–8 ns. This difference can be explained by an acceleration of the non-radiative decay resulting from the decrease of the S_1_–S_0_ gap as reflected by the large fluorescence solvatochromism.

The residual spectrum is difficult to interpret due to the weakness of the signal. As it is not largely different from that measured in CHX, it is tentatively attributed to the triplet state. The smaller triplet yield in BCN compared to CHX is consistent with the faster non-radiative decay of the S_1_ state to the ground state in polar solvents.

#### 2.3.2 Time-resolved IR absorption spectroscopy

Time-resolved IR absorption (TRIR) measurements in the –C≡C– stretching region were carried out in solvents of increasing polarity up to 2 ns. The TRIR data were also analysed globally to obtain EADS and the related time constants.

**Fig. 7:**
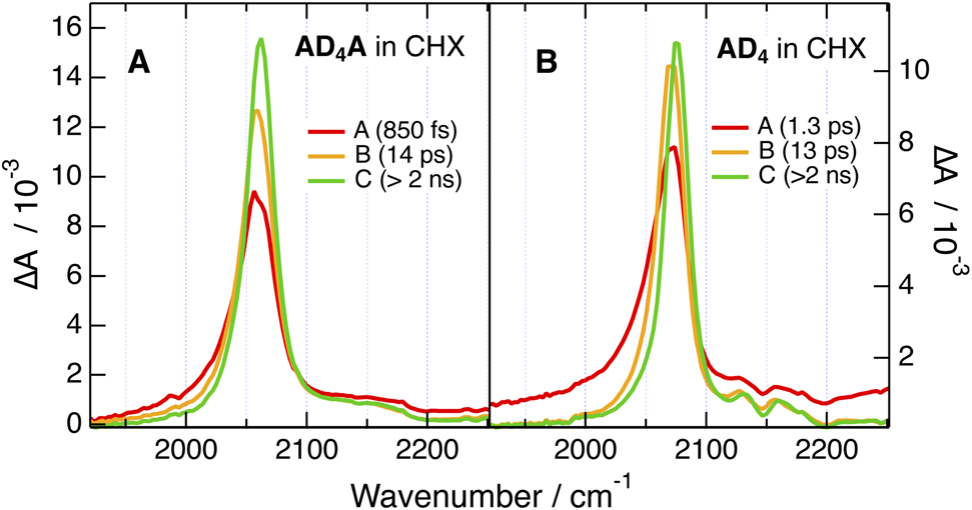
Evolution-associated difference absorption spectra and time constants obtained from a global analysis of the time-resolved IR data recorded after 400 nm excitation of (A) AD_4_A and (B) AD_4_ in cyclohexane, assuming a series of three successive exponential steps (A → B → …).

The TRIR dynamics measured in CHX are very similar for both AD_n_A. The early spectra exhibit an intense ESA band around 2060 cm^−1^ (ESA1) with a broad pedestal on its high-frequency side (ESA2), as well as a weak GSB band around 2200 cm^−1^ (Fig. 7A and S54–S57). Within about 10–20 ps, ESA1 increases, narrows and undergoes a small blue shift. Afterwards, all bands decay on a ns timescale. Considering that pumping was achieved at 400 nm in the intense S_2_ ← S_0_ absorption band, the early spectral dynamics can be attributed to the dissipation of excess excitation energy *via* vibrational relaxation,^47^ whereas the slower ns dynamics can be assigned to the decay of the equilibrated S_1_ state.

According to the calculations, AD_8_A is hardly twisted and, given that the tether is not involved in the lower-energy transitions, it can be considered as a quasi-centrosymmetric chromophore. Consequently, the intense ESA1 can be attributed to the antisymmetric –C≡C– stretching mode with its large intensity arising from the strong quadrupolar nature of the S_1_ state. The small ESA2 pedestal is attributed to the symmetric –C≡C– stretch, which is not totally IR forbidden, because the molecule is not strictly centrosymmetric. The vibrations of the ethynyl groups in 9, 10 positions of the core are not visible in the TRIR spectra. As they do not lead to significant change in dipole moment in both ground and excited states, they have negligible IR intensity.

The center of inversion is obviously lost in AD_4_A, because of its more bent structure induced by the twist. Despite this, the similarity of the TRIR spectra with those of AD_8_A points to an quasi-even distribution of the electronic excitation over both D–π–A branches as well. For the sake of simplicity, this delocalised S_1_ state will be further on called quadrupolar state, Q.

Similar spectra are observed with AD_n_, the only difference being a bleach feature around 2150 cm^−1^ overlapping with the pedestal (Fig. 7B and S58–S61). This band is also present in the stationary IR spectra of AD_n_ and can be assigned to the –C≡C– stretching mode of the core-π-TIPS branch (Fig. S50). ESA1 and ESA2 are attributed to the –C≡C– stretching mode of the D–π–A and core-π-TIPS branches of AD_n_ in the S_1_ state, respectively. The larger intensity of ESA1 is due to the large dipole moment of the D–π–A branch in the excited state. By contrast, the CT character on the core-π-TIPS branch is negligible, hence the small amplitude of ESA2.

**Fig. 8:**
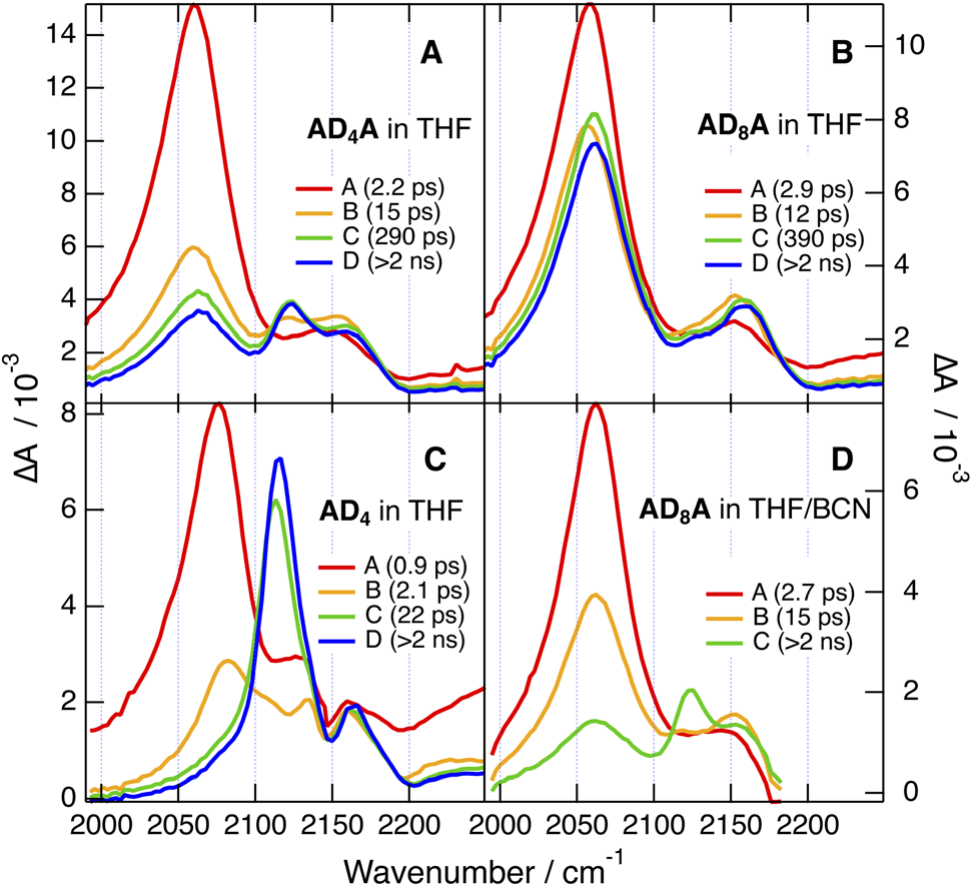
Evolution-associated difference absorption spectra and time constants obtained from a global analysis of the time-resolved IR data recorded after 400 nm excitation of AD_4_A (A), AD_8_A (B), and AD_4_ (C) in tetrahydrofuran and of AD_8_A in a tetrahydrofuran/benzonitrile mixture (D).

The early spectra measured with both AD_n_A in the medium polar THF show no significant difference from CHX, apart from a relatively more intense ESA2 (Fig. 8A, B and S57–S60). Over the first ps, ESA1 decays partially, while ESA2 grows concurrently. ESA1 remains more intense than ESA2 with AD_8_A, whereas both bands reach comparable amplitude with AD_4_A. These spectra are assigned to an intermediate state, I, with a lopsided distribution of the excitation on the two branches (Fig. 1A). As a consequence, the –C≡C– stretching vibrations become more localised on each branches, with ESA1 and ESA2 due to the stretching mode of the branch with higher and lower extent of excitation, respectively. The decrease of ESA1 and the concurrent rise of ESA2 occurring just after excitation reflect an increase in the asymmetry of the excited state. It is consistent with previous observations that ESSB takes place on timescales comparable to those of solvent motion.^24,28,48,49^ Most probably, the exciton is evenly delocalised over the whole molecule in the Franck–Condon S_1_ state and then localises partially as inertial and diffusive solvent relaxation take place. Given the sub-ps timescale of inertial motion,^44^ this early stage of ESSB is not resolved here and only the slower one due to diffusive motion is visible. The larger relative intensity of ESA2 with AD_4_A points to a more asymmetric distribution of the exciton.

After this initial stage, the spectral dynamics are no longer the same for the two dyes. In the case of AD_8_A, the spectral shape remains mostly unchanged and both bands decay on the ns timescale (Fig. 8B). For AD_4_A, the early intensity changes of ESA1 and ESA2 is rapidly followed by the rise of a new band (ESA3) around 2120 cm^−1^ between the first two. Afterwards, all three bands decay simultaneously on the ns timescale (Fig. 8A).

**Fig. 9:**
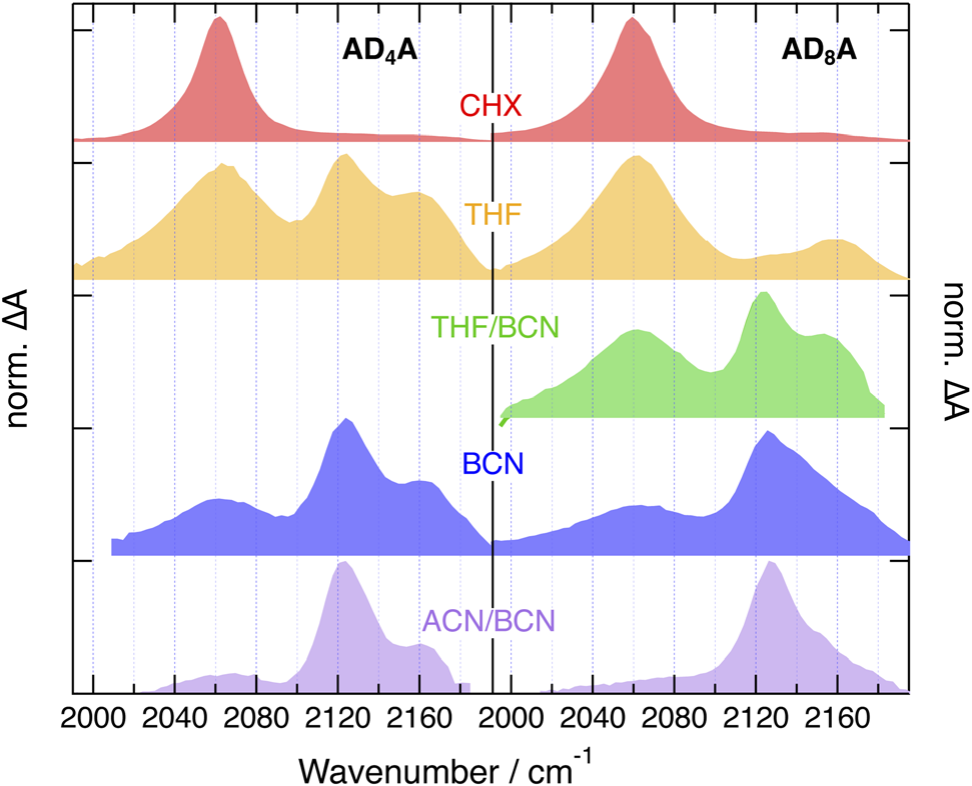
Evolution associated difference absorption spectra of the equilibrium S_1_ state of AD_n_A as a function of solvent polarity. The spectra are vertically shifted for better visualization.

Upon further increase of the solvent polarity, *i.e.* in BCN and in a 9 : 1 (v/v) acetonitrile (ACN)/BCN mixture, the TRIR spectra measured with AD_n_A do no longer depend on the length of the tether, and the spectrum of the equilibrated S_1_ state becomes increasingly dominated by ESA3 (Fig. 9 and S54–S57).

Interestingly, a band at the same frequency as ESA3 is visible in the TRIR spectra recorded a few ps after excitation of AD_n_ in THF and BCN (Fig. 8C and S58–S61). Before that, the spectra resemble those in CHX with a relatively more intense shoulder. They evolve within 2–3 ps to spectra dominated by a band at 2120 cm^−1^ like ESA3 and a small shoulder at similar position to ESA2, before decaying on the ns timescale. The early spectral dynamics are attributed to the increase of the CT character of the S_1_ state of AD_n_ upon solvent relaxation, as also suggested above for the early TA dynamics in BCN (Fig. 6C and D). As also observed with other D–π–A dyads with an ethynyl bridge, an increase of CT favours localisation of the excited electron on the acceptor to the expense of the ethyne bond.^29,50,51^ This results in an increase of the bond order and a parallel shift of the stretching frequency toward its ground state value, which is around 2200 cm^−1^ for AD_n_.

Consequently, ESA3 can be assigned to a dipolar excited state, D, with the exciton trapped on one D–π–A branch of AD_n_A. It is at higher frequency than ESA1 because of the larger CT character of the D state compared to the fully delocalised state, as explained just above with AD_n_. It is at lower frequency than ESA2, because the latter is associated the vibration of the less-excited branch of the I state and is at a frequency close to that measured in the electronic ground state.

The coexistence and parallel decay of all three bands in THF probably reflect an equilibrium between the I and D states, as already observed with other multibranched D–π–A molecules in medium polar solvents.^24,31^ Exciton trapping becomes more favoured upon increasing solvent polarity. Spectra with the three ESA bands like those measured with AD_4_A in THF can be obtained with AD_8_A when using a more polar environment, namely, a 1 : 1 (v/v) THF/BCN mixture (Fig. 8D and 9). In BCN, ESA3 is clearly visible with AD_8_A and dominates with AD_4_A. In the most polar medium used here, namely a 9 : 1 (v/v) ACN/BCN mixture, the TRIR spectrum after equilibration is almost only due to ESA3, pointing to a D S_1_ state and to full exciton localisation for both AD_n_A (Fig. 9). The presence of ESA3 in THF with AD_4_A but not AD_8_A evidences that the twisting of the anthracene core facilitates ESSB and exciton trapping.

## 3 Discussion

### 3.1 Twist-induced increase of triplet yield

Until now, the photophysical investigations of out-of-plane distortion have mostly concentrated on its effect on the ISC dynamics and the resulting triplet yield. In some cases, a clear increase of triplet yield attributed to an enhanced SOC upon twisting was detected.^5–13^ In other cases, distortion had no significant effect.^14–17^

In a previous study with anthracene twisted using the same tether as here, the triplet yield was reported to vary from 0.83 to 0.91, when changing *n* from 6 to 3, compared to 0.73 for the planar analogue.^11^ This effect was attributed to an acceleration of ISC due to a twist-induced enhancement of SOC.

In the present case, the D_n_ core is substituted in 9 and 10 positions by ethynyl groups. A close analogue is the highly fluorescent 9,10-diphenylethynyl-anthracene, which has a negligibly small triplet yield.^52^ Therefore, although twisting of the core may favour higher SOC, this might not be sufficient to make ISC very competitive with the other decay channels of the S_1_ state of AD_n_A and to result in a large triplet yield.

The TA results obtained here in CHX point unambiguously to a higher triplet yield with AD_4_A. However, according to the relative amplitude of the residual GSB band, the triplet yield is small and can be estimated to be of the order of 0.1 and <0.05 for AD_4_A and AD_8_A, respectively. The higher triplet yield of AD_4_A agrees with its smaller fluorescence quantum yield, and is consistent with the shorter lifetime of its S_1_ state, namely 6 *vs.* 8 ns (Fig. 6A and B).

In principle, the increasing triplet yield upon distortion could be due to a concurrent decrease of the singlet–triplet gap and an acceleration of ISC. TD-DFT calculations predict the presence of four triplet excited state below S_1_, but do not point to significant changes in the S_1_–T_*n*_ gaps upon torsion (Fig. S62). Calculations of the SOC were not conclusive as no significant increase with torsion were predicted. However, given the small triplet yield, the slow ISC dynamics and the fact that all four triplet states can contribute, even minor changes in SOC for each ISC pathway could result in the small increase in triplet yield observed here.

**Fig. 10:**
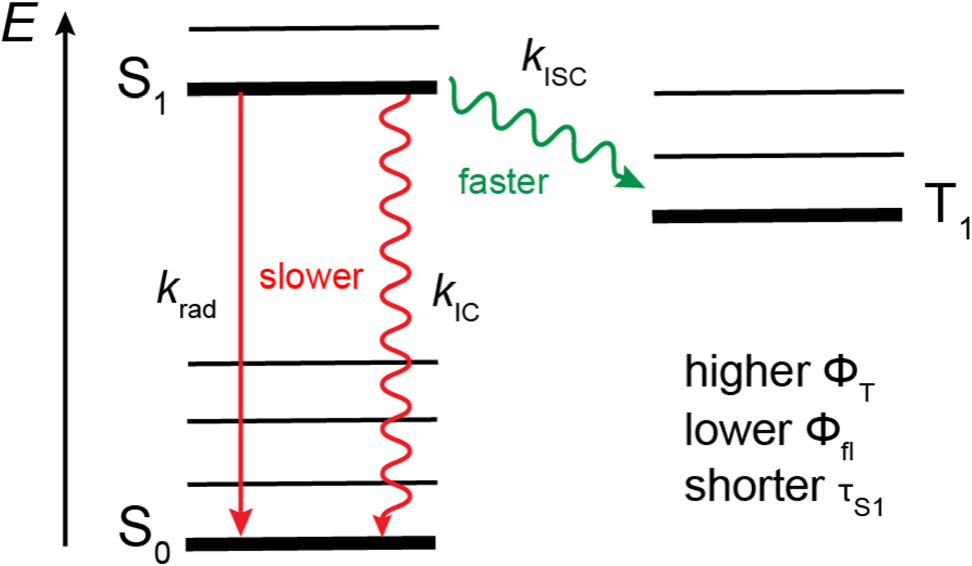
Jablonski diagram illustrating the effect of the twist on the decay pathways of the single excited state of AD_n_A. For the sake of simplicity, only the lowest triplet state is represented.

Apart from enhancing SOC, out-of-plane distortion can also affect the other decay pathways of the S_1_ state, *i.e.*, fluorescence and internal conversion (Fig. 10). TD-DFT calculations predict the S_1_ ← S_0_ oscillator strength to be smaller for AD_4_A than AD_8_A, namely, 0.54 *vs.* 0.60. This is consistent with the smaller intensity of the SE band measured with AD_4_A in CHX (Fig. 6A and B) and points to a decrease of the radiative rate constant, *k*_rad_, upon twisting, as observed with anthracene and perylene bisimide.^11,17^ The short tether in AD_4_A could also rigidify the structure of the molecule. The intensity ratio of the 0–0 and first vibronic band, *I*_00_/*I*_10_, in the electronic absorption and emissions spectra is larger for AD_4_A than AD_8_A and points to smaller geometry changes for AD_4_A upon S_1_ ← S_0_ excitation. This in turn can be expected to affect the Franck–Condon factors and to slow down S_1_ → S_0_ internal conversion (IC).^17^ Slower radiative decay and internal conversion but faster ISC upon increasing torsion can account for the observed decrease of fluorescence quantum yield and excited-state lifetime. Therefore, the higher triplet yield is consistent with a twist-induced enhancement of SOC (Fig. 10).

### 3.2 Twist-induced exciton trapping

**Fig. 11:**
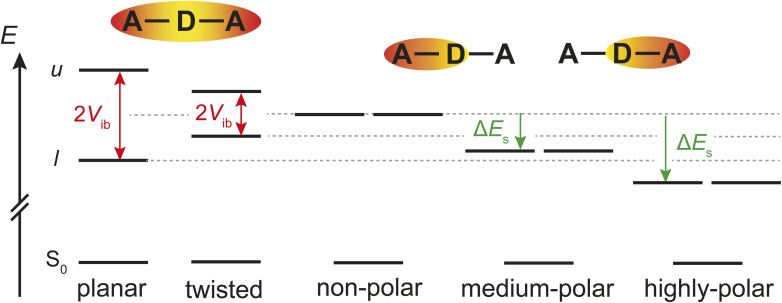
Qualitative illustration of the energetics of excited-state symmetry breaking and the effect of twisting distortion. Upon localisation of the exciton, the stabilisation of the lowest excitonic state (*l*, corresponding here to the S_1_ state) *via* interbranch coupling, *V*_ib_, is lost and the S_1_ state becomes doubly degenerate with the exciton on either the left or right side. In polar media, solvation energy is larger by Δ*E*_s_ for the localised dipolar excited state than for the delocalised quadrupolar state. Localisation of the excitation occurs when −Δ*E*_s_ > – *V*_ib_. As *V*_ib_ decreases upon twisting, symmetry breaking occurs in a less polar solvent than for a planar molecule. Increase and decrease of electronic density are represented in red and yellow, respectively.

The TRIR results reveal that the nature of the excited state of AD_n_A in a medium polar environment is affected by the distortion of the anthracene core. The twist-induced ESSB observed with AD_4_A in THF can be explained in terms of a simple excitonic model,^53–55^ by considering that each D–π–A branch is a single chromophore with a CT transition (Fig. 11). Interbranch coupling, *V*_ib_, results in two excitonic states (*u* and *l* in Fig. 11) with the excitation evenly distributed over the whole molecule and with a Davydov splitting of 2*V*_ib_. This coupling stabilises the lowest delocalised excited state, *i.e.* the Q state, relative to the localised D state. Therefore, ESSB and exciton trapping should in principle not be energetically feasible, unless there is a mechanism that stabilises the D state relatively to the Q state. Such mechanism is present in polar media, where dipolar solvation energy is significantly larger for the localised D state than the delocalised Q state.^29,30^ If the gain of solvation energy upon localisation of the charges compensates for the loss of interbranch coupling, the D state is more stable than the Q state and ESSB takes place.^29,30^ According to this picture, the fact that ESSB occurs in a less polar solvent with AD_4_A than with AD_8_A points to a smaller interbranch coupling for the former (Fig. 11).

In principle, *V*_ib_ corresponds to half the gap between the upper and lower excitonic states, *u* and *l* (Fig. 11). However, as the *u* ← S_0_ transition is one-photon forbidden for a centrosymmetric chromophore,^56^ it is not really possible to detect it, especially considering the continuous absorption spectrum below 530 nm. The same problem was encountered with the quantum-chemical calculations. The upper excitonic state could not be clearly identified, most probably because of its mixing with other nearby states. However, the markedly more intense SE band measured in CHX with AD_8_A relatively to AD_4_A points to a larger S_1_ → S_0_ transition dipole for AD_8_A, in agreement with a stronger interbranch coupling.

Two mechanisms, one through space and one through bond, could contribute to this difference in *V*_ib_. The first involves the dipolar interactions between the branches. Kasha's excitonic model predicts the S_1_–S_0_ transition dipole of the delocalised Q state of a linear double-branched dye to be larger by a factor of 
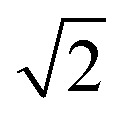
 than that of a single branch.^57^ This agrees well with the larger amplitude of the SE band in the TA spectra of AD_n_A compared to AD_n_. The larger bending angle of AD_4_A relative to AD_8_A predicted by the calculations should result in a weaker dipolar interaction in AD_4_A. The second mechanism involves exchange interaction through the anthracene core.^53^ Such through-bond contribution to the interbranch coupling can be expected to be reduced upon distortion of the π conjugated system. Independently of which of these two mechanisms contributes most to the interbranch coupling, the twist results in smaller *V*_ib_ and consequently a less polar solvent is required for ESSB to be operative and for the trapping of the exciton on a single branch.

## 4 Conclusion

We investigated here how out-of-plane distortion of the central aromatic core of a double-branch D–π–A molecule affects the nature of the excited state and its dynamics. The donor twisting strongly affects the chiroptical properties, with a significant increase of the ECD response and CPL activity for AD_4_A. The main goal of previous studies was to exploit distortion for increasing spin–orbit coupling to accelerate intersystem crossing and achieve large triplet yield without heavy atoms. Such twist-induced enhancement of spin–orbit coupling is also observed here, although the triplet yield remains relatively small.

Our TRIR results reveal unambiguously that ESSB, hence exciton trapping, is facilitated by the twist of the core. Consequently, this phenomenon is operative in a medium polar solvent like THF, whereas a markedly more polar environment is required in the absence of distortion. This higher tendency toward the localisation of the exciton upon twisting arises from the decrease of interbranch coupling. Consequently, the loss of this coupling upon localisation needs a less polar solvent to be compensated for. Previous investigations showed how the propensity for ESSB and exciton trapping can be tuned upon varying the electron donating and accepting strength of the constituents, the length of the D–π–A branches as well as their position on the central core. Here, we could show that it can also be modified without any change in the chemical nature of the molecule, but by mere distortion. In principle, such distortion could also be induced in a constrained environment without the need of a tether. These findings are also relevant for our understanding of the excited-state properties of large conjugated systems and could be used to better control the spatial distribution of the electronic excitation and potentially their photochemistry.

## Author contributions

C. G. carried out the stationary and time-resolved photophysical measurements and contributed to the quantum-chemical calculations. J. S. O. O. and J. C. measured and analysed the CPL data. E. V., O. G. and I. S. conceived the project and wrote the manuscript with the input from all authors.

## Conflicts of interest

There are no conflicts to declare.

## Supplementary Material

SC-OLF-D5SC06393K-s001

## Data Availability

All data can be downloaded from: https://doi.org/10.26037/yareta:j5ecxqy32jcehjnbc4nopl4shm. Supplementary information (SI): synthesis and characterization, experimental and computational details, additional results: photophysical and chiroptical data, solvatochromism, transient electronic absorption data, time-resolved IR data, X-ray diffraction. See DOI: https://doi.org/10.1039/d5sc06393k.
