# Supplementary material for: Exciton trapping with a twist
Source: Chem Sci. 2025 Dec 18;17(6):3056–65. doi: 10.1039/d5sc06393k (PMC12714126; doi:10.1039/d5sc06393k)
Supplement: SC-017-D5SC06393K-s001 [file SC-017-D5SC06393K-s001.pdf]

Supplementary Information (SI) for Chemical Science.
This journal is © The Royal Society of Chemistry 2025

Supporting information

Exciton Trapping with a Twist

Chinju Govind,[†,¶] Israa Shioukhi,[‡,¶] Yinon Deree,[‡] Jhon Sebastian Oviedo Ortiz,[§] Jeanne Crassous,[§] Ori Gidron,[*,‡] and Eric Vauthey[*,†]

†*Department of Physical Chemistry, University of Geneva, CH-1211 Geneva, Switzerland.*

‡*Institute of Chemistry, The Hebrew University of Jerusalem, Givat Ram, Jerusalem, Israel 9190401*

§*University of Rennes, CNRS, ISCR – UMR 6226, 35000 Rennes, France*

¶*Contributed equally to this work*

E-mail: ori.gidron@mail.huji.ac.il; eric.vauthey@unige.ch

Contents

S1 General 1

S2 Synthesis 4

S2.1 General procedure (**A**) for the synthesis of **1-D$_n$** 4

S2.2 General procedure (**B**) for the synthesis of **AD$_n$** 4

S2.3 General procedure (**C**) for the synthesis of **AD$_n$A** 5

S3 Characterization 6

S3.1 NMR 6

S3.2 Mass spectroscopy 27

S3.2.1 MALDI 27

S4 Photophysical and chiroptical properties 29

S4.1 Electronic absorption spectra 29

S4.2 ECD spectra 32

S4.3 Stationary fluorescence spectra 33

S4.4 Solvatochromism 36

S4.5 Fluorescence excitation spectra 40

S4.6 Fluorescence quantum yields 40

S4.7 Circularly polarized luminescence (CPL) 41

S4.7.1 **AD$_4$A** 41

S4.7.2. **AD$_8$A** 41

S4.8 Stationary vibrational spectroscopy 42

S5. Time-resolved spectroscopic measurements 42

S5.1 Electronic transient absorption spectroscopy 42

S5.2 Time-resolved IR spectroscopy 45

S6 Single crystal X-ray diffraction crystallography (SCXRD) 52

S7 Quantum-chemical calculations 53

S8 References 55

S1 General

Commercially available reagents and chemicals were used without further purification unless otherwise stated. Di-TIPS-$\mathbf{D_n}$ and 6-Bromo-2-hexyl-1H-benzo[de]isoquinoline-1,3(2H)-dione (6-**Br-NI**) were synthesized according to previously published methods. [1,2]

Flash chromatography (FC) was performed using CombiFlash SiO_2 columns. Chiral HPLC separations were performed with a Chiralpak® IG semi-preparative column and CHIRALPAK® IB-N (250 × 4.6 mm / 5μm) preparative columns, with hexane/dichloromethane as eluent.

^{1}H and ^{13}C NMR spectra were recorded in solution on a Bruker-Neo 400 MHz and 500 MHz spectrometers using the ^{1}H signal of tetramethylsilane (TMS) or the residual solvent peak that had been previously calibrated to TMS as the external standard. ^{13}C-NMR spectra were referenced to the ^{1}H frequency multiplied by the standard factor of 0.25145020. ^{13}C-NMR spectra were ^{1}H decoupled. The spectra were recorded using chloroform-*d*. Chemical shifts (δ) are expressed in ppm.

UV-vis absorption spectra were recorded using an Agilent Cary 5000 spectrophotometer. The spectra were measured using a quartz cuvette (1 cm) at 25 °C. The absorption wavelengths are reported in nm with the extinction coefficient ε ($M^{-1}cm^{-1}$) in brackets.

Steady-state fluorescence measurements were performed on a HORIBA JOBIN YVON Fluoromax-4 spectrofluorometer with the excitation/emission geometry at right angles. Fluorescence quantum yields (Θ_f) were determined using an integrating sphere. The lifetimes of the excited species were measured using an NL-C2 Pulsed Diode Controller NanoLED light source with time-correlated single photon counting (TSCPC) Controller DeltaHub (HORIBA), referenced against colloidal Ludox solution (50 wt. % solution in water) obtained from Aldrich. Electronic Circular Dichroism (ECD) spectra were recorded on a MOS-500 spectrophotometer from BioLogic Science Instruments.

Circular polarized luminescence (CPL) measurements were performed using a home-built CPL spectrofluoropolarimeter (constructed with the help of the JASCO Company). The samples were excited using a 90° geometry with a xenon ozone-free lamp 150 W LS.

High-resolution mass spectrometry (HRMS) measurements were performed using a Q Exactive *Plus* mass spectrometer (Thermo Fisher Scientific) equipped with an electrospray ionization (ESI) source operated in positive ion mode. Compounds were introduced by direct injection through a non-interacting column. Using 100% acetonitrile as the mobile phase. MALDI-TOF/TOF autoflex speed mass spectrometer (Bruker Daltonilk GmbH, Bremen, Germany)

equipped with a smartbeam-II solid-state laser (modified Nd:YAG laser) λ = 355 nm. The instrument was operated in positive ion, reflection mode. The accelerating voltage was 21.0 kV. The delay time was 130 ns. Laser fluence was optimized for each sample. The laser was fired at a frequency of 2 kHz and spectra were accumulated in multiples of 500 laser shots to achieve 1500 shots in total. Sample preparation: 2-[(2E)-3-(4-tert-Butylphenyl)-2-methylprop2- enylidene] malononitrile (DCTB) matrix solutions were made to a concentration of 20 mg/mL in dichloromethane (DCM). Sample solutions were made to an approximate concentration of 5 mg/mL in DCM. Sample and matrix solutions were premixed at a ratio of 1:9 or 1:40 (v/v). A volume of 0.5 µL of this mixture was deposited onto a MALDI steel target plate. After evaporation of the solvent, the target was inserted into the mass spectrometer.

X-Ray diffraction data sets and solution for crystals of *rac*-**AD$_4$A** were collected by mounting a single crystal on a MiTeGen MicroLoops E™, then using a XtaLAB Synergy-S, Single source at offset/far, HyPix diffractometer equipped with oxford Cryostream 800 operating at T = 100.0(1) K. Data were measured using MoKα radiation. Using Olex2, the structure was solved with the SHELXT structure solution program using Intrinsic Phasing and refined with the SHELXL refinement package using Least Squares minimization.

Electronic transient absorption (TA) measurements were performed with a setup described in detail previously.[3] Briefly, excitation was carried out with 400 nm pulses generated by frequency doubling part of the output of a regeneratively amplified Ti:Sapphire system (Spectra-Physics, Solstice, 800 nm, 35 fs, 5 KHz). Probing was achieved with a white light supercontinuum (320 - 750 nm) generated by focusing a fraction of the Solstice output into a 3 mm CaF_2 plate. The polarization of the pump pulses was at magic angle with respect to the probe pulses. The pump fluence at the sample position was about 0.3 mJ/cm^2. The samples were located in a 1 mm pathlength cell under constant nitrogen bubbling. The FWHM of the IRF was around 100 - 400 fs depending on the wavelength.

TA measurements on the ns-µs timescales were performed with a setup described in detail in ref.[4] Excitation was done at 355 nm using a passively Q-switched, frequency tripled Nd:YAG laser (Teem Photonics, Powerchip NanoUV) producing 300 ps pulses at 500 Hz. The pump fluence on the sample was around 2 mJ/cm^2. Probing was achieved as in the fs-ps transient absorption experiment. The FWHM of the IRF was around 370 ps.

Femtosecond time-resolved IR (TRIR) spectra were obtained using a homebuilt setup based on a Ti:Sapphire amplified system (Spectra Physics Solstice) producing 100 fs pulses at 800 nm

and 1 kHz repetition rate as described in detail previously.[5] Excitation was carried out 400 nm by frequency doubling a fraction of the amplifier output. The polarization was controlled with a combination of Glan-Laser polarizer and zero-order half-wave plate, limiting the time resolution of the experiment to 300 fs. The pulses were focused on the sample onto 350 μm spot, resulting in a fluence of 0.05-0.3 mJ/cm^2. Mid-IR probe pulses at around 4.7-5.2 μm were generated by difference frequency mixing of the output of an optical parametric amplifier (Light Conversion, TOPAS-C with NDFG module) that was pumped at 800 nm. The polarization of the IR beam was controlled using a wire-grid polarizer. Two horizontally polarized IR beams were produced with a CaF_2 wedge and focused onto the sample. One of the beams was overlapped with the pump beam, whereas the second was used as reference. Both IR beams were focused onto the entrance slit of an imaging spectrograph (Horiba, Triax 190, 150 lines/mm) equipped with a liquid nitrogen cooled 2 x 64 element MCT array (Infrared Systems Development), giving a resolution of 3-4 cm^{-1} in the -C≡C- stretching region. The average of 500 signal shots was taken to collect one data point with the polarization of the pump pulses at the magic angle with respect to that of the IR pulse. This procedure was carried out for at least four times depending on the signal reproducibility and intensity.

S2 Synthesis

S2.1 General procedure (A) for the synthesis of **1-D$_n$**

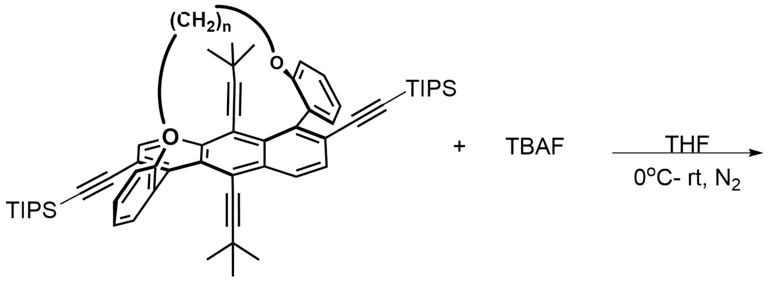

n= 4, 8

n= 4, 8

To a solution of Di-TIPS-Dn (0.053 mmol) in 20 mL of THF, kept under Argon in a single-neck RB flask, tetrabutylammonium fluoride (TBAF) solution (1 M in THF, 0.05 mmol) was added dropwise at 0°C. The reaction mixture was then stirred at room temperature for an additional 2 h. The THF was evaporated, and the resulting crude was directly subjected to silica gel column chromatography using DCM/hexane (1:10) to obtain a bright yellow solid.

S2.2 General procedure (B) for the synthesis of AD$_n$

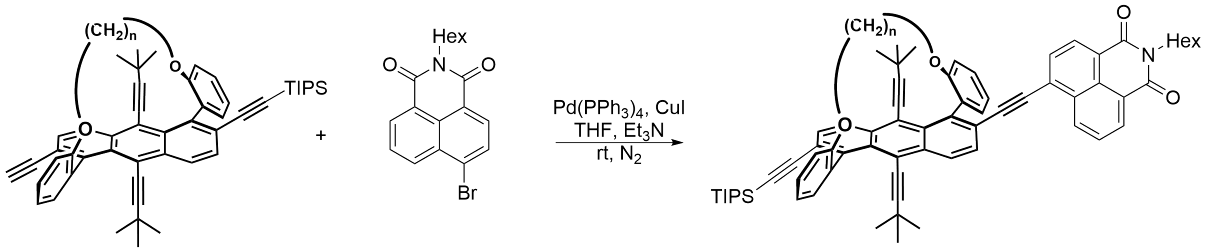

n= 4, 8

n= 4, 8

Under an inert atmosphere, enantiopure **1-D$_n$** (0.025mmol), 6-Bromo-2-hexyl-1H-benzo[de]isoquinoline-1,3(2H)-dione (0.032 mmol), $Pd(PPh_3)_4$ (1.4 mg, 5% mol, 0.00125 mmol), and CuI (0.05 mg, 10% mol, 0.0025 mmol) were added to an oven-dried one-necked round bottomed flask equipped with a magnetic stirrer. A mixture (4 mL) of dry trimethylamine and tetrahydrofuran (1:1) was added to the previously mixed reagents. The reaction mixture was stirred for 24 hours at room temperature. The solvents were evaporated and the reaction mixture was loaded into a silica gel column. A 10% of a mixture of ethyl acetate in hexane was used to obtain the desired products.

S2.3 General procedure (C) for the synthesis of AD_nA

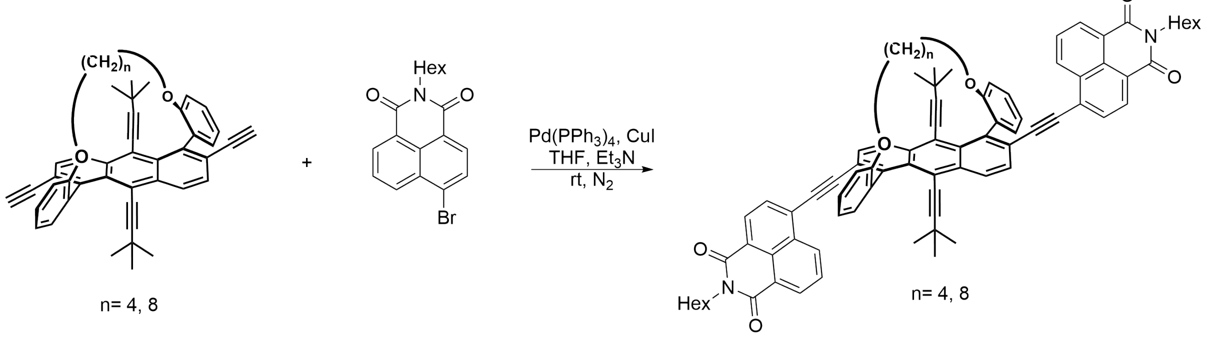

Under an inert atmosphere, enantiopure **2-D$_n$** (0.044 mmol), 6-Bromo-2-hexyl-1H-benzo[de]isoquinoline-1,3(2H)-dione (0.11 mmol), $Pd(PPh_3)_4$ (2.55 mg, 5% mol, 0.0022 mmol), and CuI (0.084 mg, 10% mol, 0.0044 mmol) were added to an oven-dried one-necked round bottomed flask equipped with a magnetic stirrer. A mixture (4 mL) of dry trimethylamine and tetrahydrofuran (1:1) was added to the previously mixed reagents. The reaction mixture was stirred for 24 hours at room temperature. The solvents were evaporated and the reaction mixture was loaded into a silica gel column. A 20% of a mixture of ethyl acetate in hexane was used to obtain the desired products.

S3 Characterization

S3.1 NMR

1-D₈

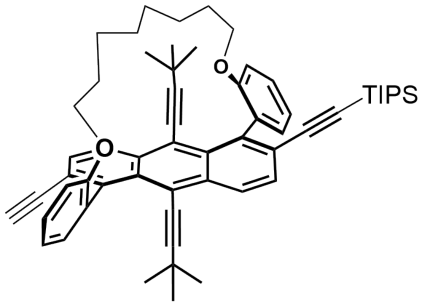

Following the general procedure **A**, 53 mg of Di-TIPS-**D_8** was used to obtain **1-D_8** as a yellow solid (35 mg, 78 % yield).

^{1}H NMR (500 MHz, $CDCl_3$) δ 8.65 (d, *J* = 9.2 Hz, 1H), 8.62 (d, *J* = 9.2 Hz, 1H), 7.57 (d, *J* = 7.4 Hz, 1H), 7.55 (d, *J* = 7.5 Hz, 1H), 7.43 (ddd, *J* = 8.2, 7.4, 1.7 Hz, 1H), 7.39 (dd, *J* = 7.4, 1.7 Hz, 1H), 7.36 – 7.31 (m, 2H), 7.06 (td, *J* = 7.4, 1.0 Hz, 1H), 6.99 (td, *J* = 7.4, 1.0 Hz, 1H), 6.93 (dd, *J* = 8.1, 1.0 Hz, 1H), 6.89 – 6.81 (m, 1H), 3.96 (dt, *J* = 8.7, 4.3 Hz, 1H), 3.94 – 3.87 (m, 1H), 3.77 (dtd, *J* = 10.4, 8.8, 4.4 Hz, 2H), 2.98 (s, 1H), 1.38 – 1.24 (m, 3H), 1.13 (s, 11H), 1.13 (s, 16H), 0.96 (d, *J* = 3.3 Hz, 7H), 0.69 (q, *J* = 7.9 Hz, 1H), 0.63 – 0.46 (m, 4H). **^{13}C NMR** (126 MHz, $CDCl_3$) δ 157.87 (d, *J* = 3.4 Hz), 141.60, 141.05, 134.83 (d, *J* = 2.0 Hz), 132.31, 131.45 (d, *J* = 2.7 Hz), 130.18, 129.81, 129.59, 129.31, 129.18, 128.96, 127.77, 127.47, 123.67, 122.05, 120.43, 120.26, 119.90, 119.85, 115.96, 112.11, 111.80, 107.57, 95.95, 84.24, 81.64, 78.89, 69.28 (d, *J* = 7.2 Hz), 31.04, 31.01, 29.89, 29.72, 29.55, 29.33, 28.70, 27.34, 26.96, 18.78, 11.36.

HR-ESI-MS m/z (100%): 837.5019 (100, $[M]^+$) calcd. for $C_{59}H_{69}O_2Si^+$: 837.50613.

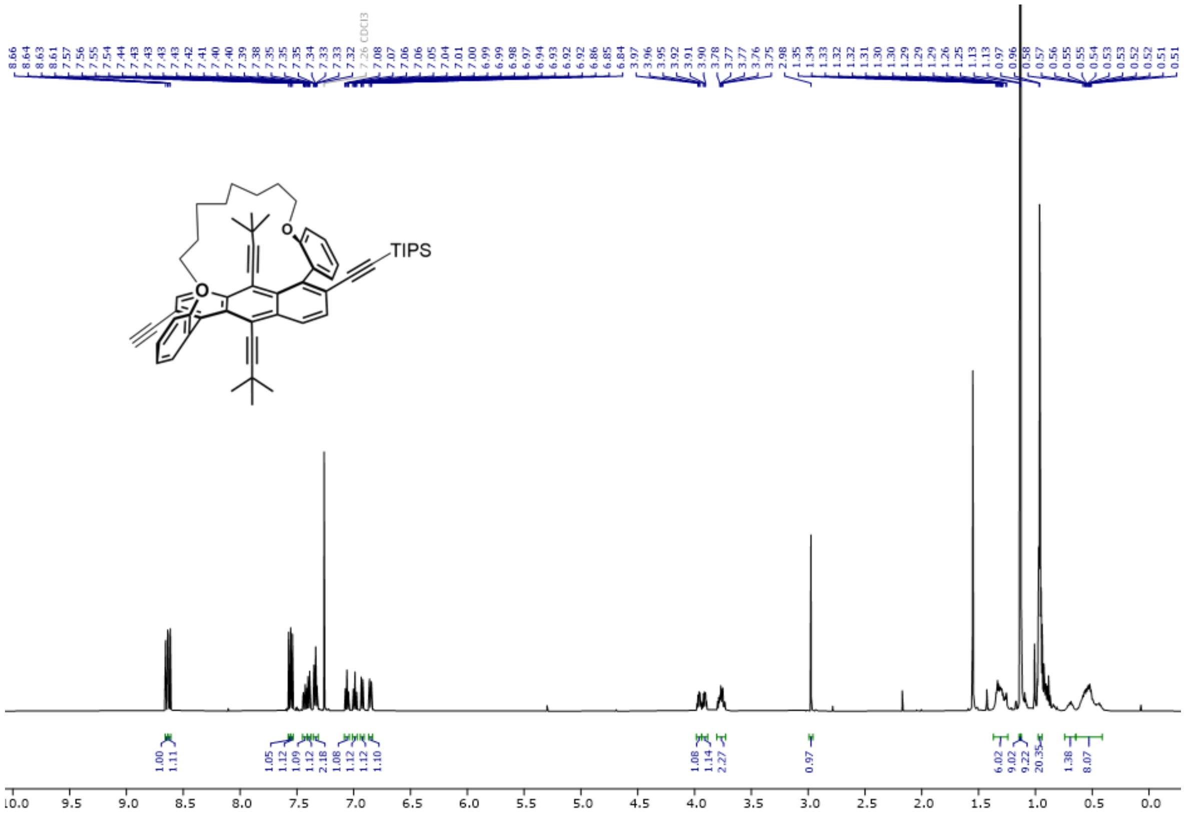

Figure S1. ^{1}H NMR (500 MHz) of **1-D$_8$** in $CDCl_3$, measured at 298 K.

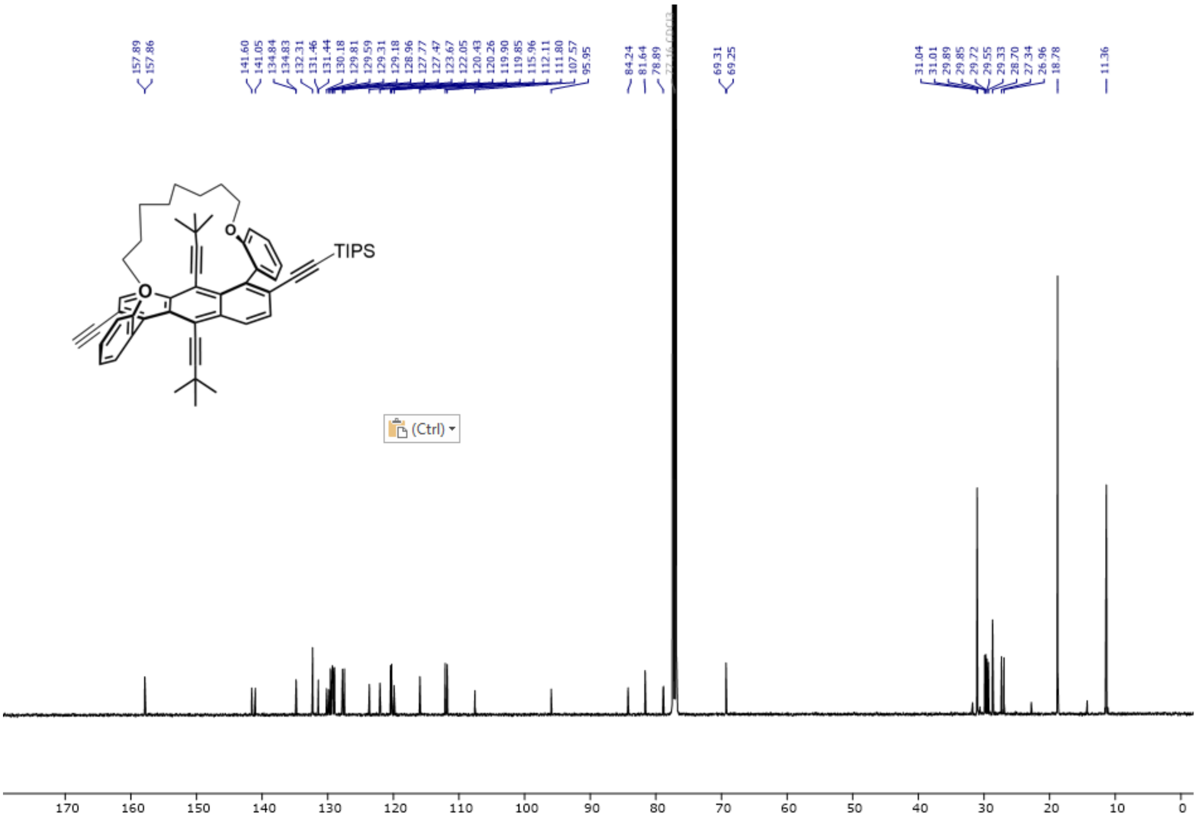

Figure S2. ^{13}C NMR (126 MHz) of **1-D$_8$** in $CDCl_3$, measured at 298 K.

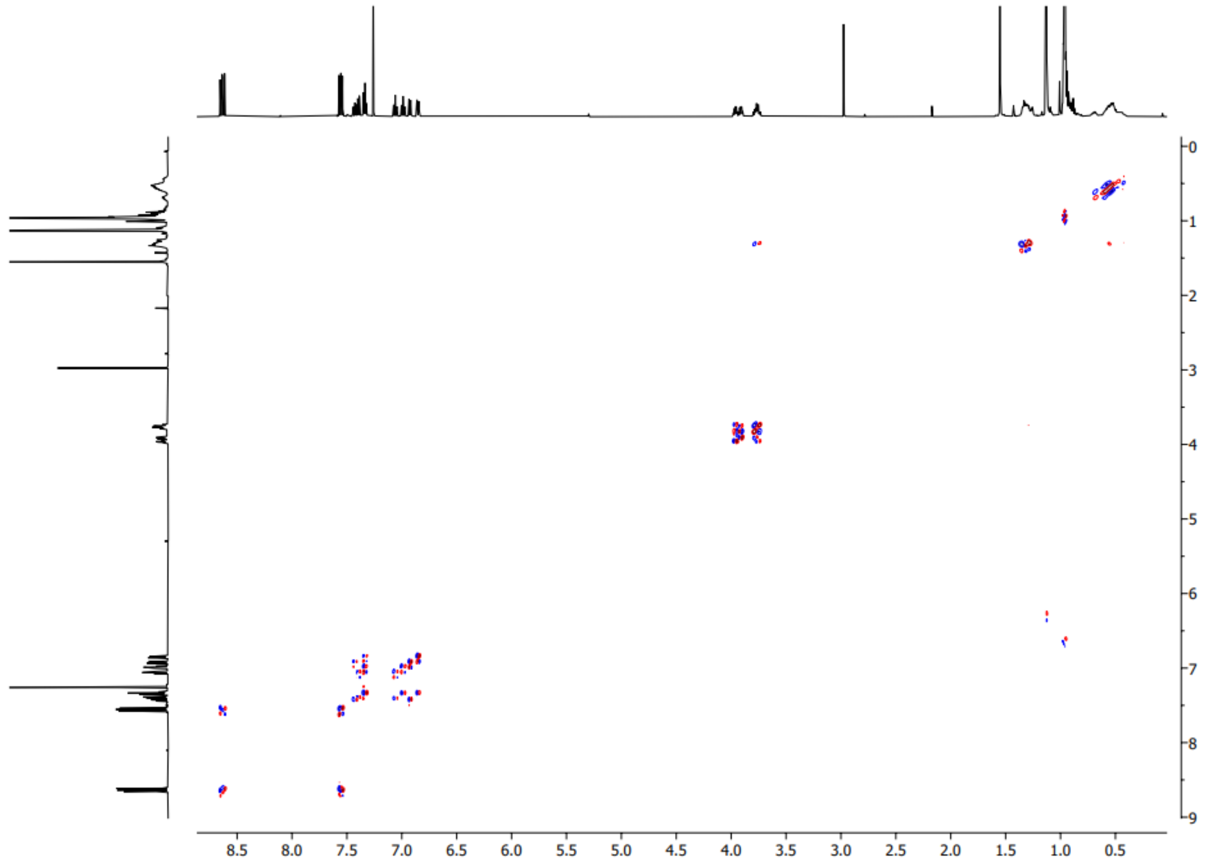

Figure S3. COSY (500 MHz) of **1-D$_8$** in $CDCl_3$, measured at 298 K.

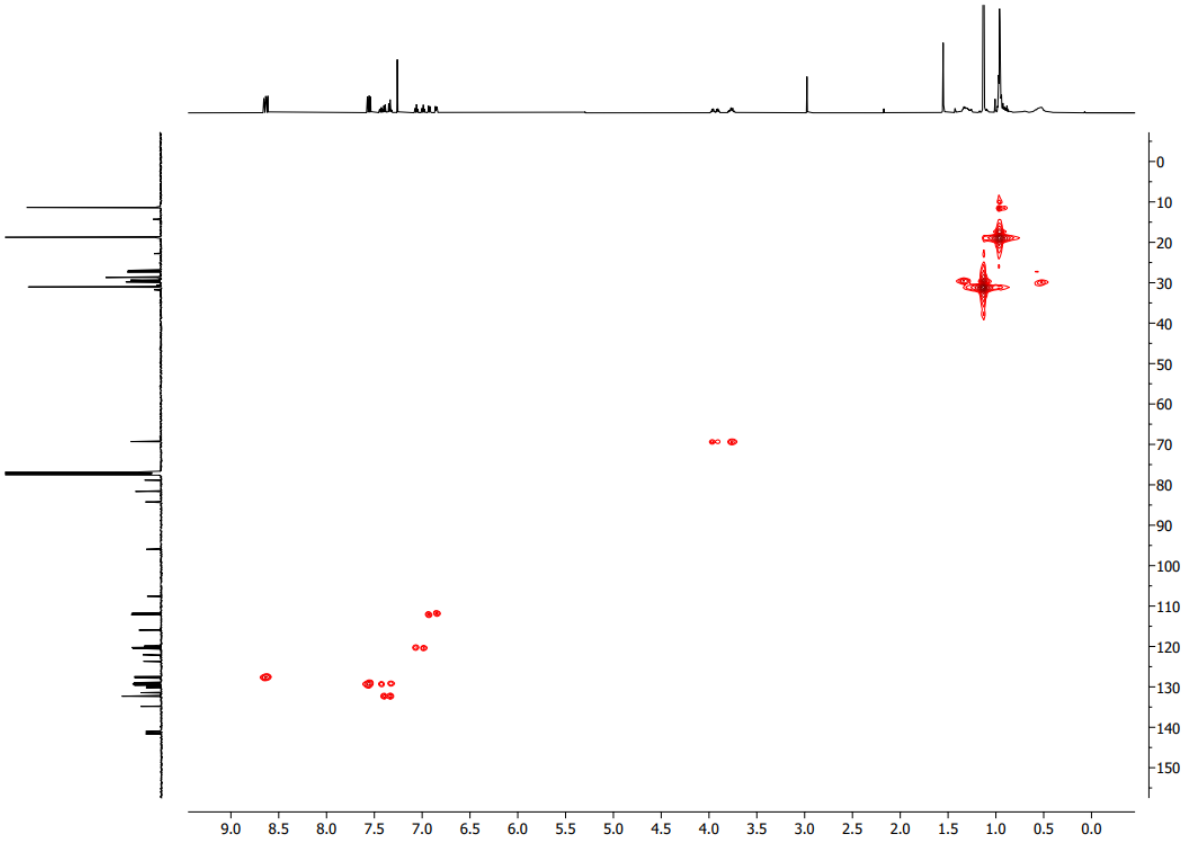

Figure S4. HSQC (500 MHz) of **1-D$_8$** in $CDCl_3$, measured at 298 K.

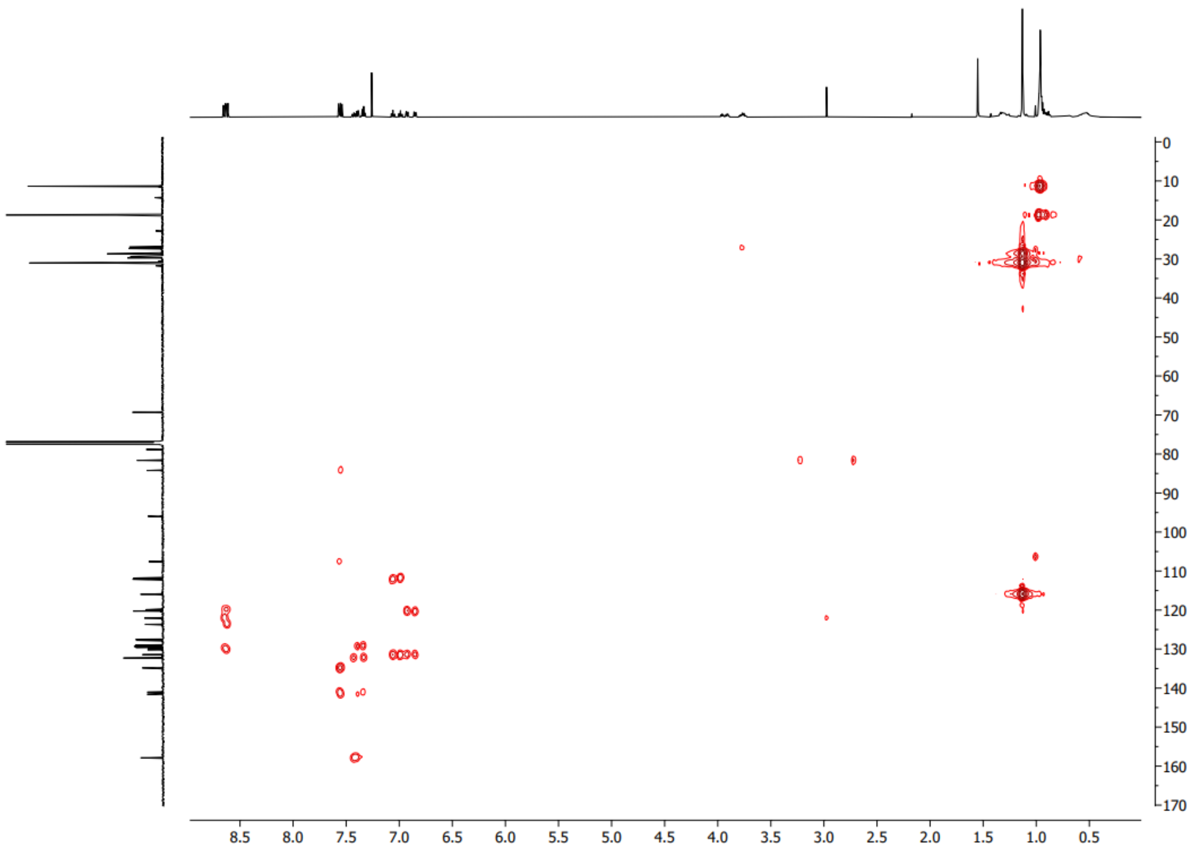

Figure S5. HMBC (500 MHz) of **1-D$_8$** in $CDCl_3$, measured at 298 K.

1-D$_4$

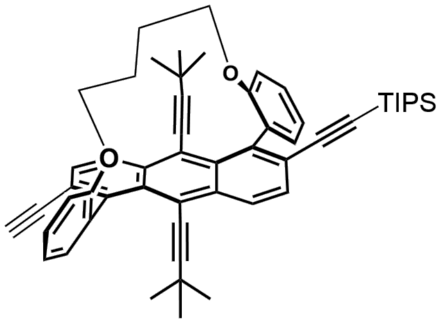

Following the general procedure **A**, 50 mg of Di-TIPS-**D$_4$** was used to obtain **1-D$_4$** as a yellow solid (37 mg, 88 % yield).

^{1}H NMR (400 MHz, $CDCl_3$) δ 8.25 (d, *J* = 7.2 Hz, 2H), 8.22 (d, *J* = 7.2 Hz, 2H), 7.90 – 7.85 (m, 2H), 7.46 (t, *J* = 9.1 Hz, 1H), 7.26 (td, *J* = 7.8, 1.7 Hz, 1H), 7.22 – 7.16 (m, 1H), 7.03 (td, *J* = 7.5, 1.0 Hz, 1H), 6.96 (td, *J* = 7.5, 1.1 Hz, 1H), 6.60 (d, *J* = 8.1 Hz, 1H), 6.55 (d, *J* = 8.1 Hz, 1H), 3.64 – 3.49 (m, 2H), 3.34 – 3.14 (m, 2H), 3.02 (s, 1H), 1.03 (d, *J* = 1.1 Hz, 18H), 0.96 (s, 21H), 0.77 – 0.63 (m, 2H), 0.49 (qd, *J* = 12.6, 4.1 Hz, 2H). **^{13}C NMR** (101 MHz, $CDCl_3$) δ 156.03 (d, J = 5.0 Hz), 140.04, 139.25, 133.10, 131.89, 130.09, 129.65, 129.49, 129.37,

129.28, 128.97, 126.28, 125.97, 122.28, 120.64, 119.93, 119.48, 118.77, 118.62, 113.85 (d, J = 2.2 Hz), 110.23, 110.07, 107.72, 96.17, 84.43, 81.74, 66.10 (d, J = 3.1 Hz), 30.91 (d, J = 2.1 Hz), 28.51, 26.21, 26.12, 18.80 (d, J = 1.5 Hz), 11.50.

HR-ESI-MS m/z (100%): 780.43453(100, $[M]^+$) calcd. for $C_{55}H_{60}O_2Si^+$: 780.43571.

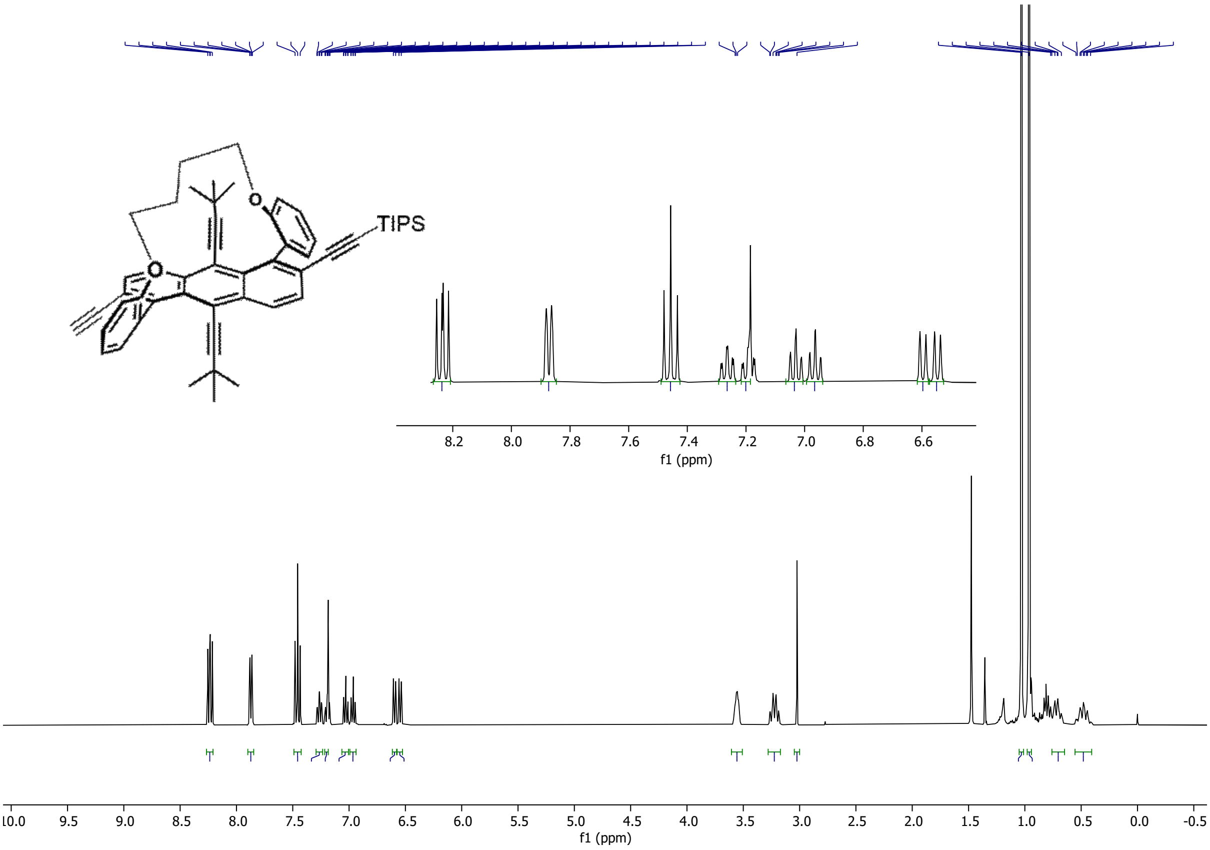

Figure S6. ^{1}H NMR (400 MHz) of **1-D$_4$** in $CDCl_3$, measured at 298 K.

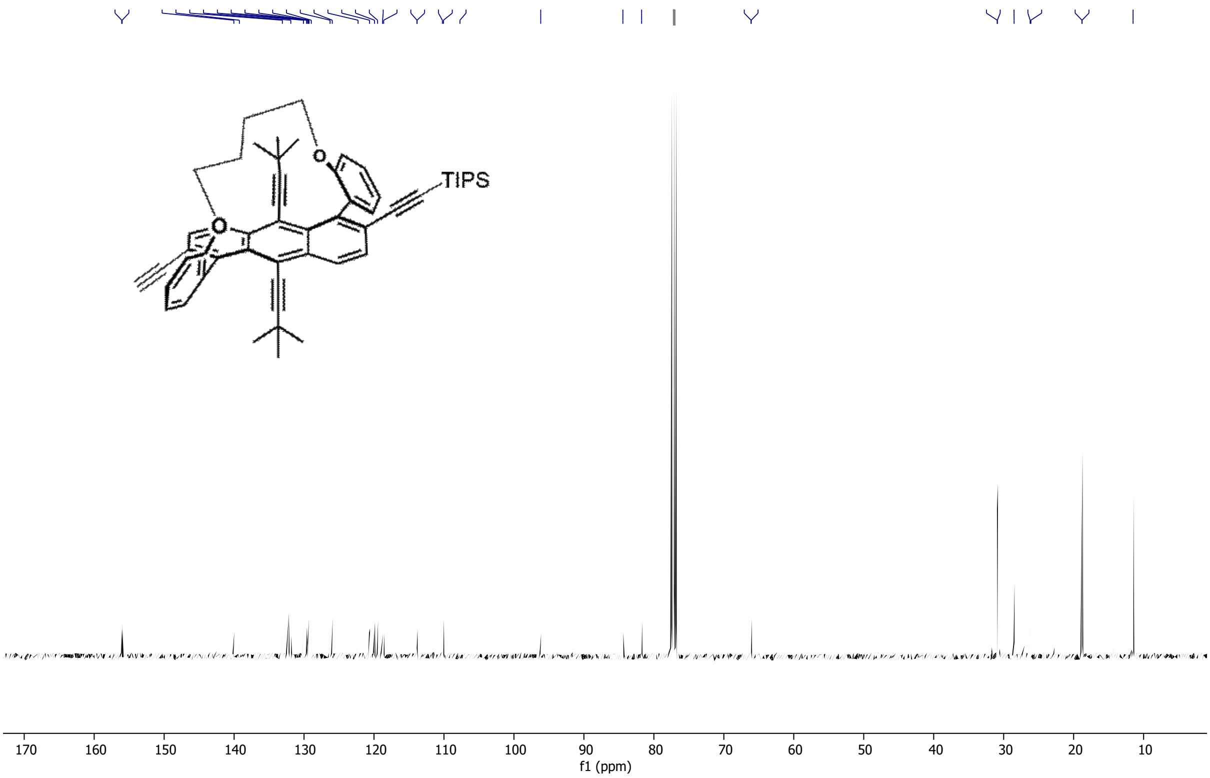

Figure S7. ^{13}C NMR (101 MHz) of **1-D$_4$** in $CDCl_3$, measured at 298 K.

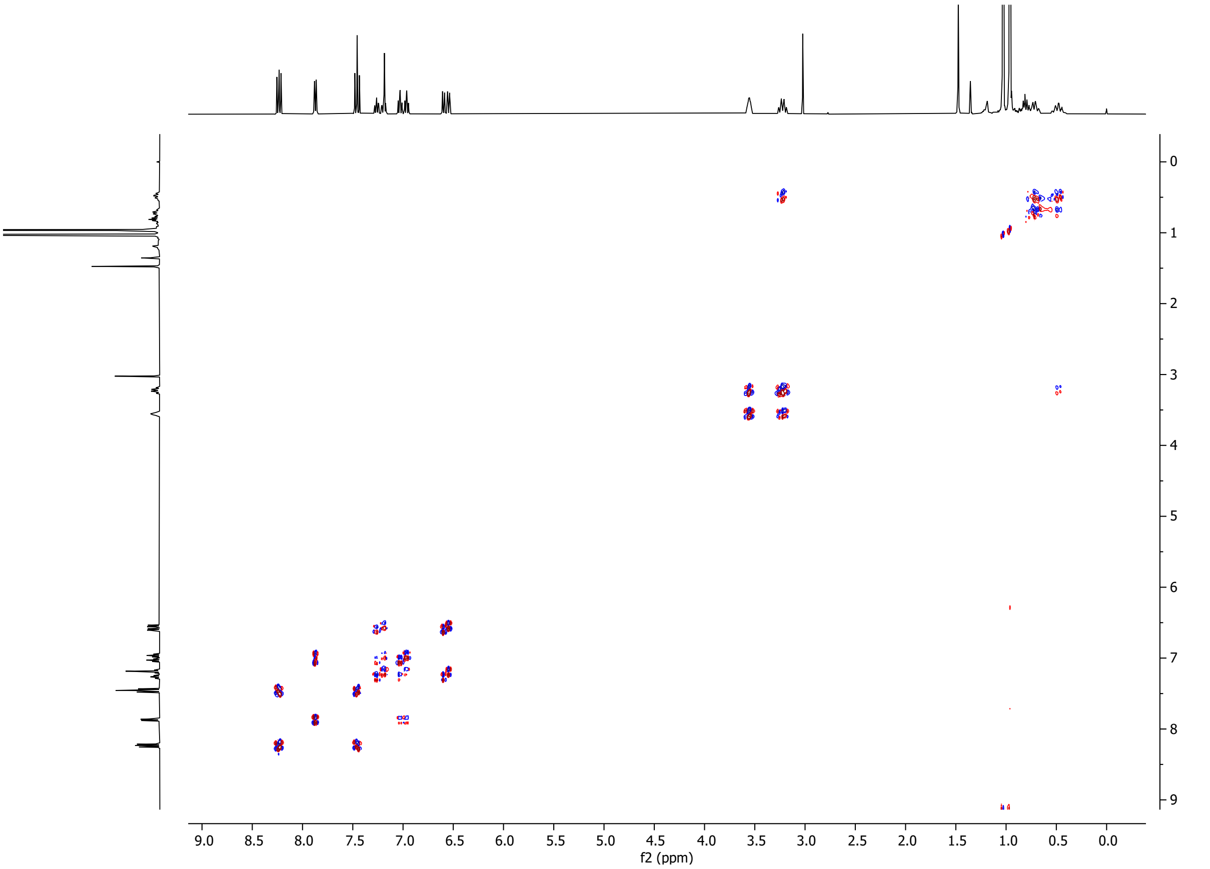

Figure S8. COSY (400 MHz) of **1-D$_4$** in $CDCl_3$, measured at 298 K.

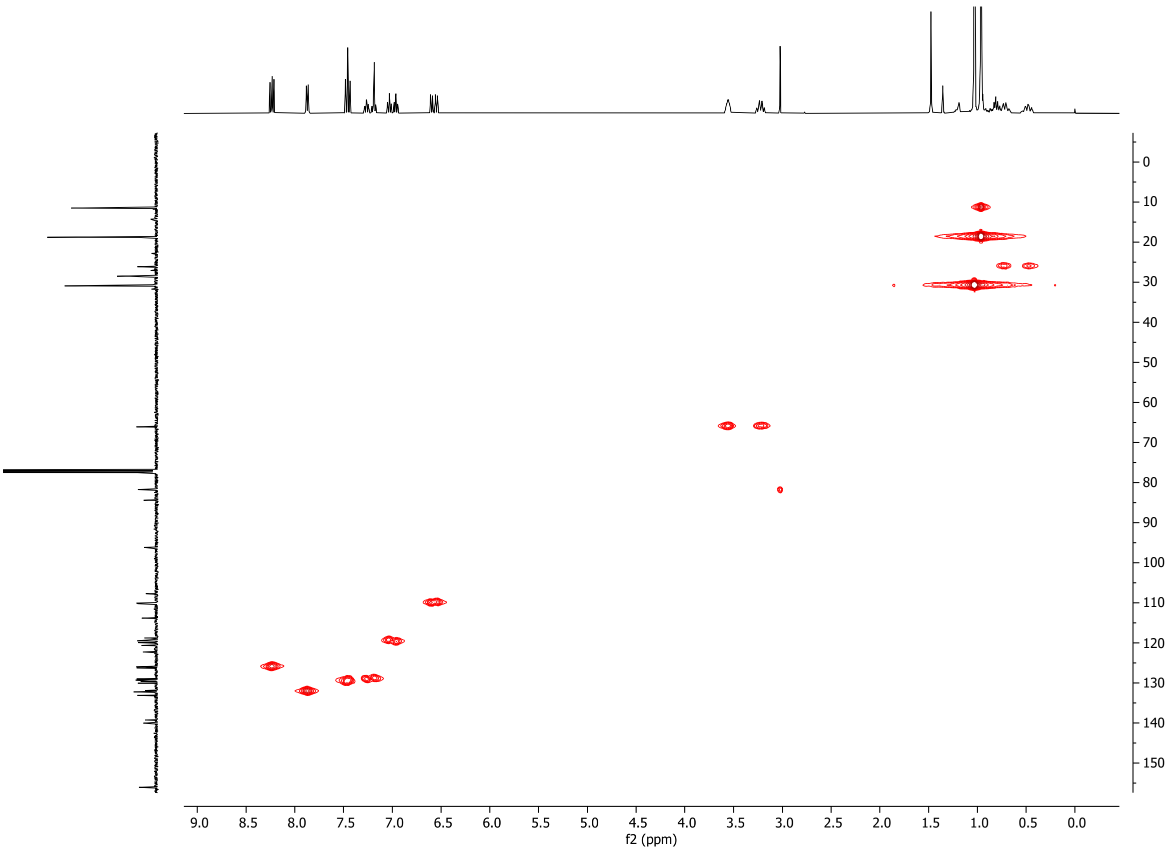

Figure S9. HSQC (400 MHz) of **1-D$_4$** in $CDCl_3$, measured at 298 K.

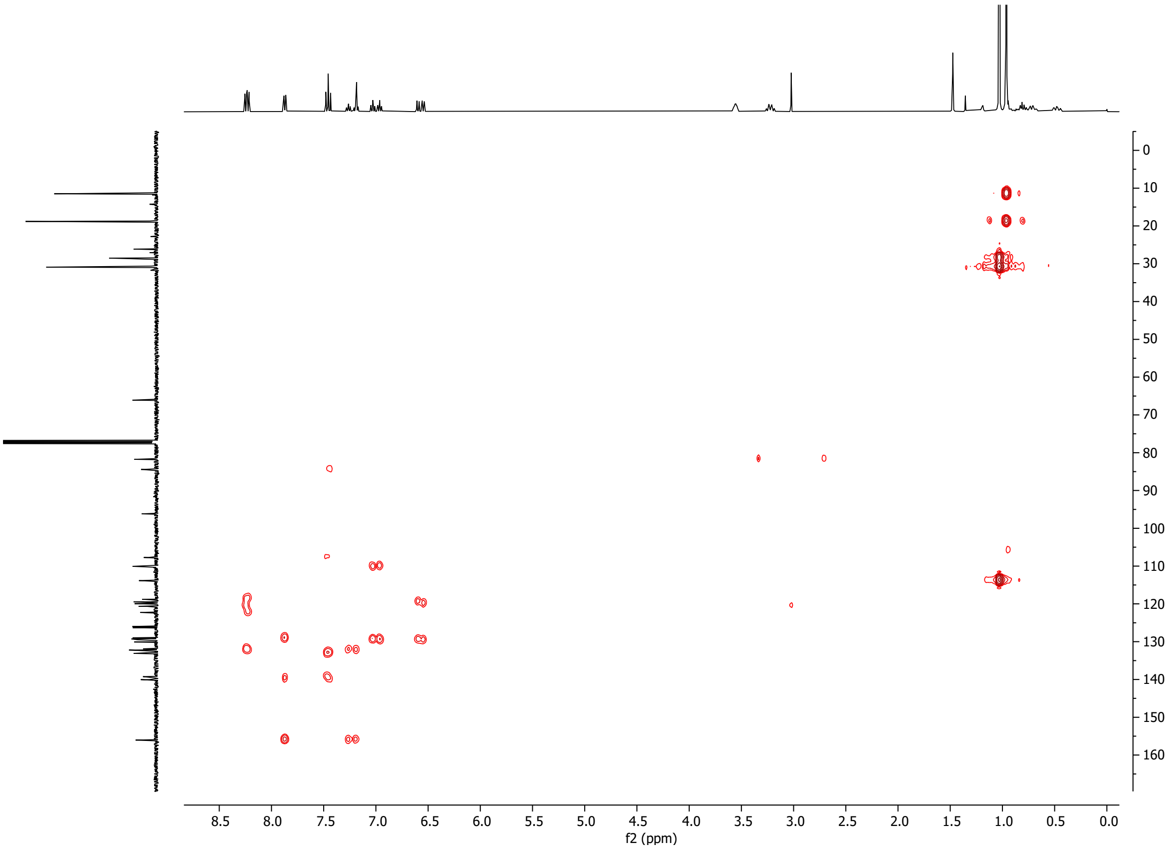

Figure S10. HMBC (400 MHz) of **1-D$_4$** in $CDCl_3$, measured at 298 K.

AD_8

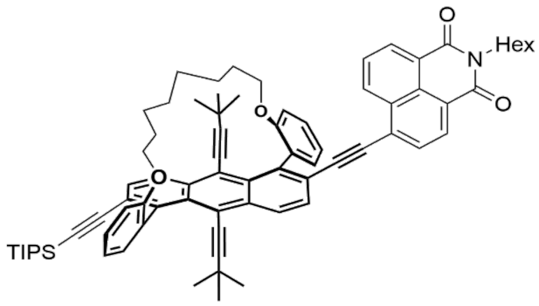

Following the general procedure **B**, 22 mg of **1-D$_8$** and 12 mg of 6-**Br-NI** were used to obtain **AD$_8$** as a yellow solid (18 mg, 61 % yield).

^{1}H NMR (400 MHz, $CDCl_3$) δ 8.76 (d, J = 9.2 Hz, 1H), 8.65 (d, J = 9.2 Hz, 1H), 8.57 (dd, J = 7.3, 1.2 Hz, 1H), 8.47 (d, J = 7.7 Hz, 1H), 7.79 (dd, J = 8.4, 1.2 Hz, 1H), 7.74 (d, J = 9.2 Hz, 1H), 7.70 – 7.61 (m, 2H), 7.60 (d, J = 9.0 Hz, 2H), 7.51 (dd, J = 7.4, 1.7 Hz, 1H), 7.36 (t, J = 7.6 Hz, 2H), 7.14 (td, J = 7.5, 1.0 Hz, 1H), 7.06 – 6.97 (m, 2H), 6.87 (d, J = 8.1 Hz, 1H), 4.19 – 4.09 (m, 2H), 3.98 (dt, J = 8.7, 4.2 Hz, 1H), 3.92 (dt, J = 8.8, 4.3 Hz, 1H), 3.80 (h, J = 4.2 Hz, 2H), 1.71 (p, J = 7.5 Hz, 2H), 1.41 (m, J = 7.7 Hz, 2H), 1.17 (s, 9H), 1.16 (s, 9H), 0.98 (s, 2H), 0.97 (s, 18H), 0.93 – 0.79 (m, 4H), 0.76 – 0.67 (m, 1H), 0.65 – 0.46 (m, 6H). **^{13}C NMR** (126 MHz, $CDCl_3$) δ 171.13, 164.12, 163.81, 158.09, 157.76, 141.72, 140.94, 134.87 (d, J = 1.2 Hz), 133.20, 132.42, 132.17, 131.61, 131.38, 131.36, 131.26, 130.53, 130.28, 129.68, 129.66, 129.30, 129.11, 128.33, 128.19, 128.05, 127.91, 127.36, 126.97, 123.80, 122.64, 122.10, 121.61, 120.46, 120.34, 119.96 (d, J = 1.3 Hz), 116.18, 116.05, 112.07, 111.74, 107.34, 100.74, 96.15, 91.24, 78.82, 78.65, 69.20 (d, J = 10.7 Hz), 60.38, 40.50, 31.55, 30.97, 30.86, 29.77, 29.63, 29.39, 29.21, 28.62, 28.59, 28.06, 27.15, 26.91, 26.79, 22.55, 21.03, 18.62, 14.04, 11.21.

HR-ESI-MS m/z (100%): 1116.62828 (100, $[M]^+$) calcd. for $C_{77}H_{85}NO_4Si^+$: 1116.63206.

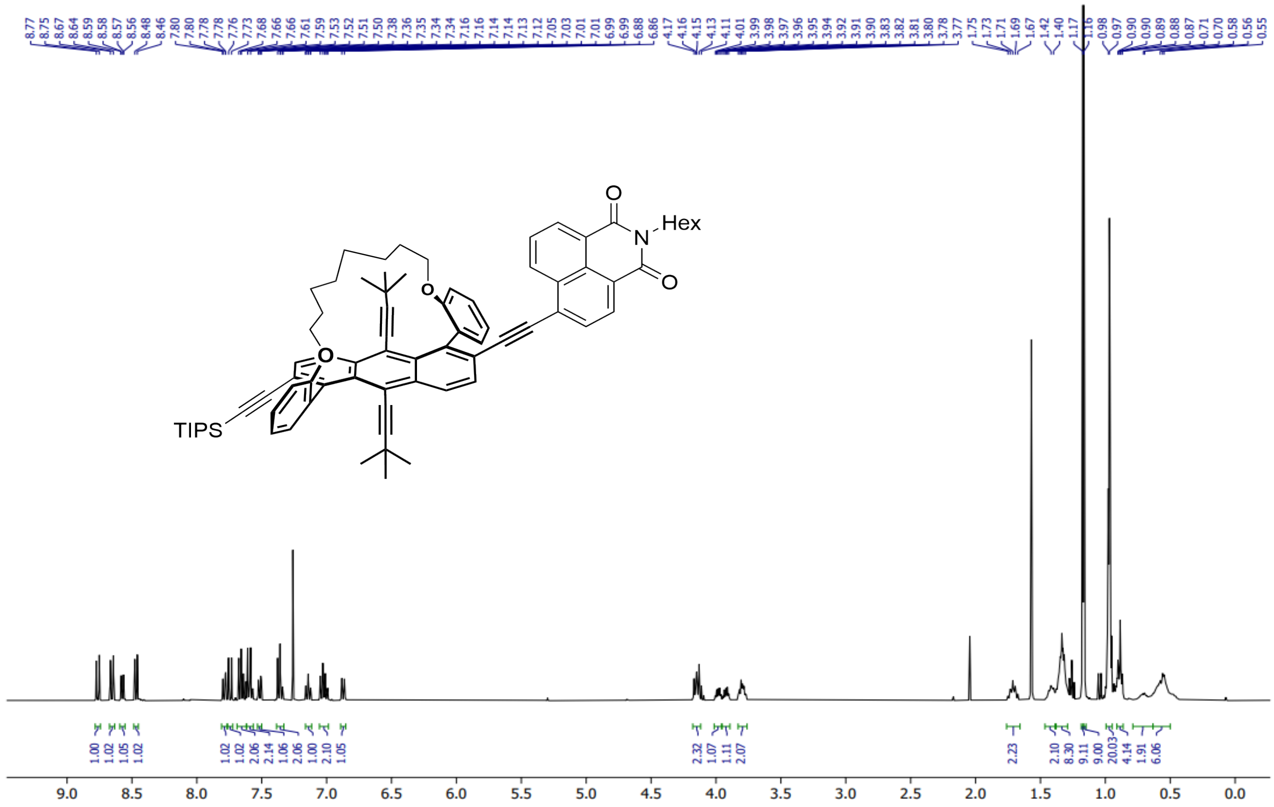

Figure S11. ^{1}H NMR (500 MHz) of **AD$_8$** in $CDCl_3$, measured at 298 K.

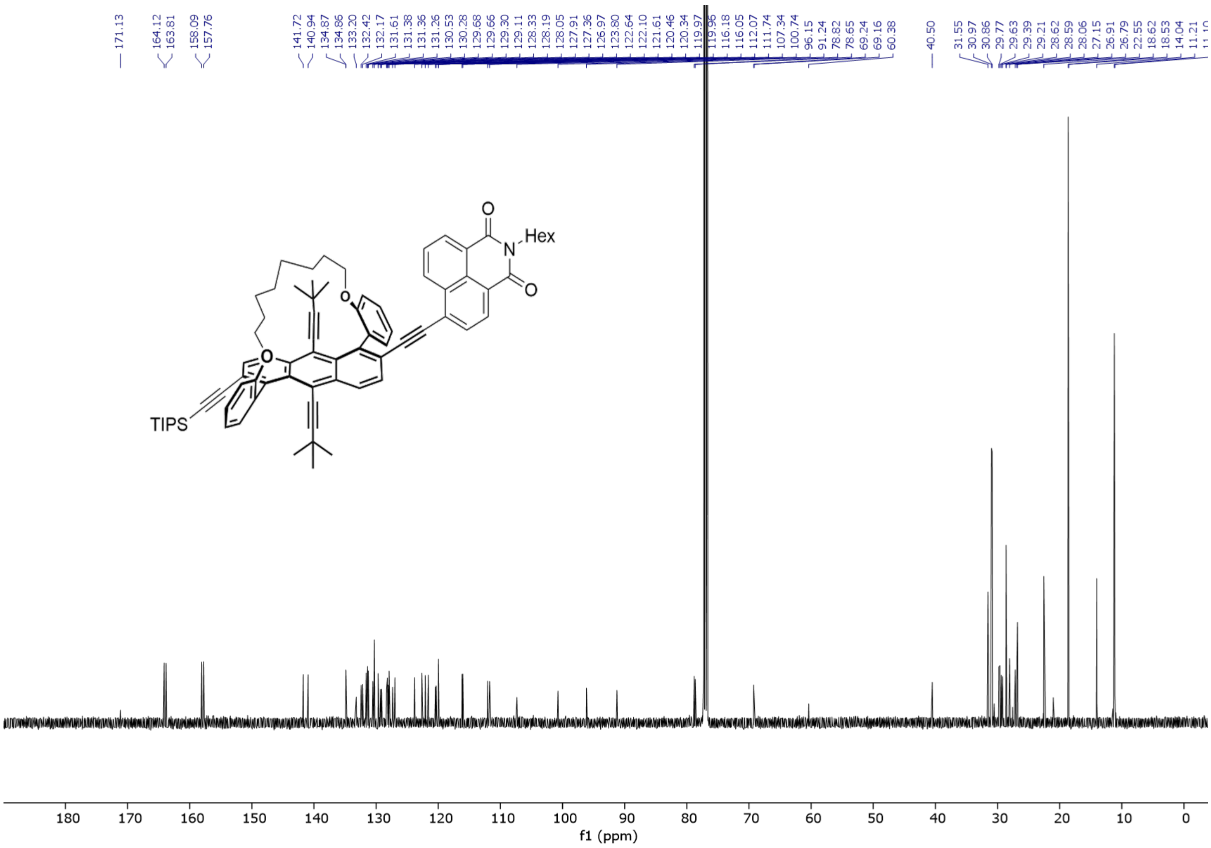

Figure S12. ^{13}C NMR (126 MHz) of **AD$_8$** in $CDCl_3$, measured at 298 K.

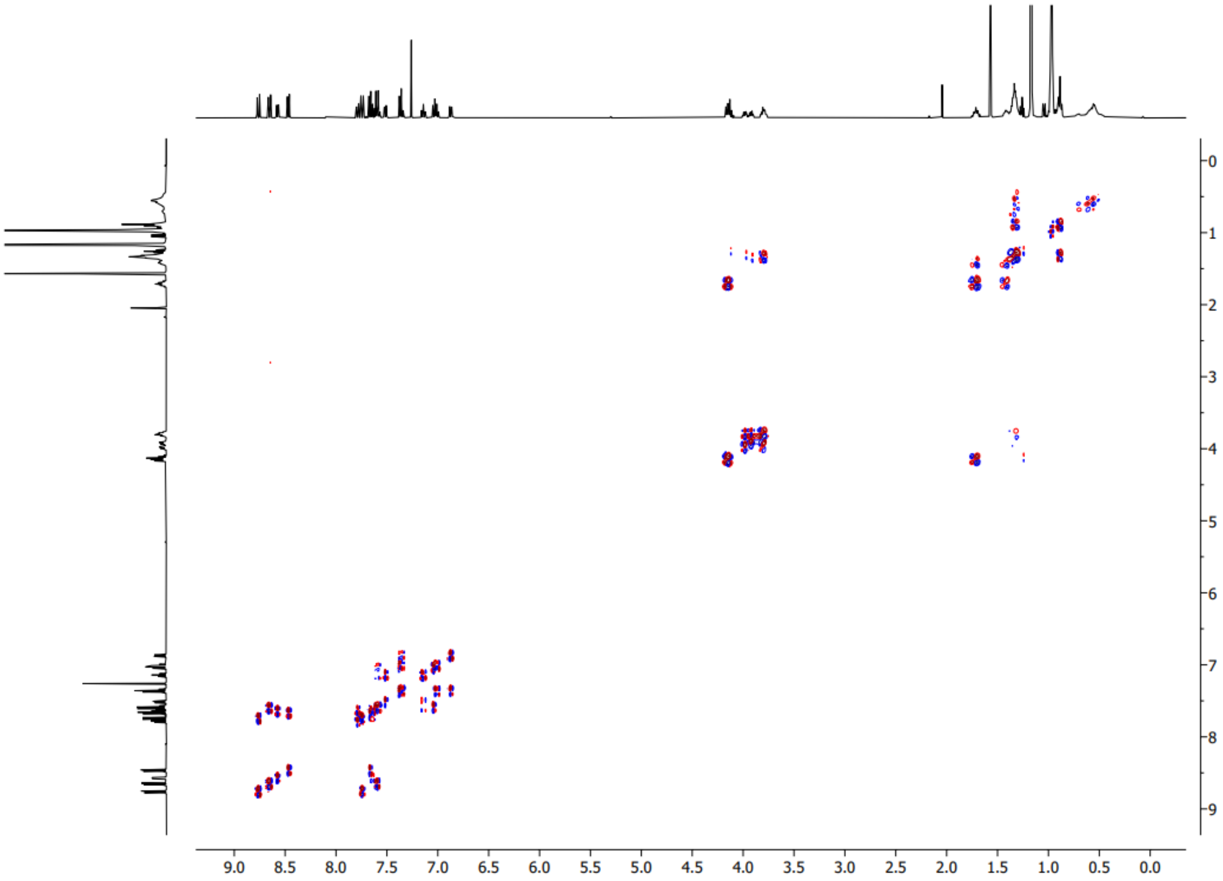

Figure S13. COSY (500 MHz) of $\mathbf{AD_8}$ in $CDCl_3$, measured at 298 K.

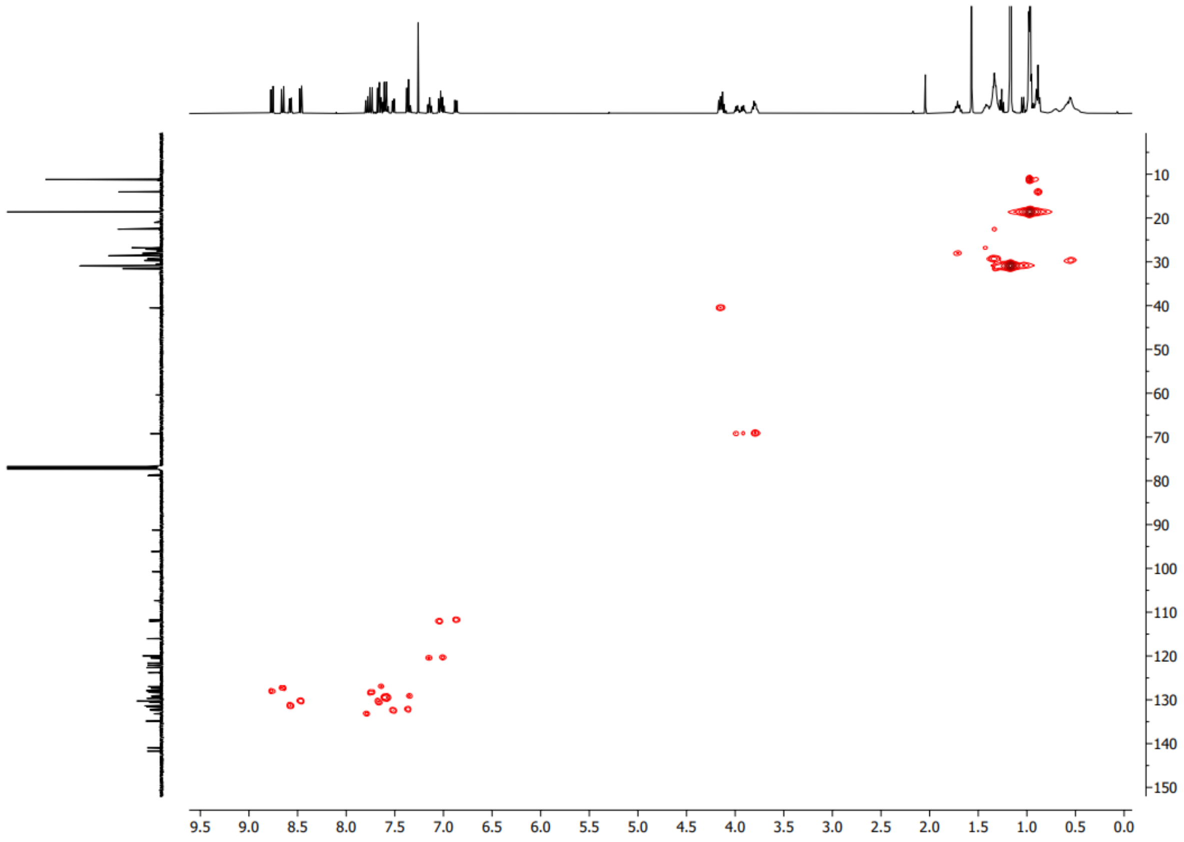

Figure S14. HSQC (500 MHz) of $\mathbf{AD_8}$ in $CDCl_3$, measured at 298 K.

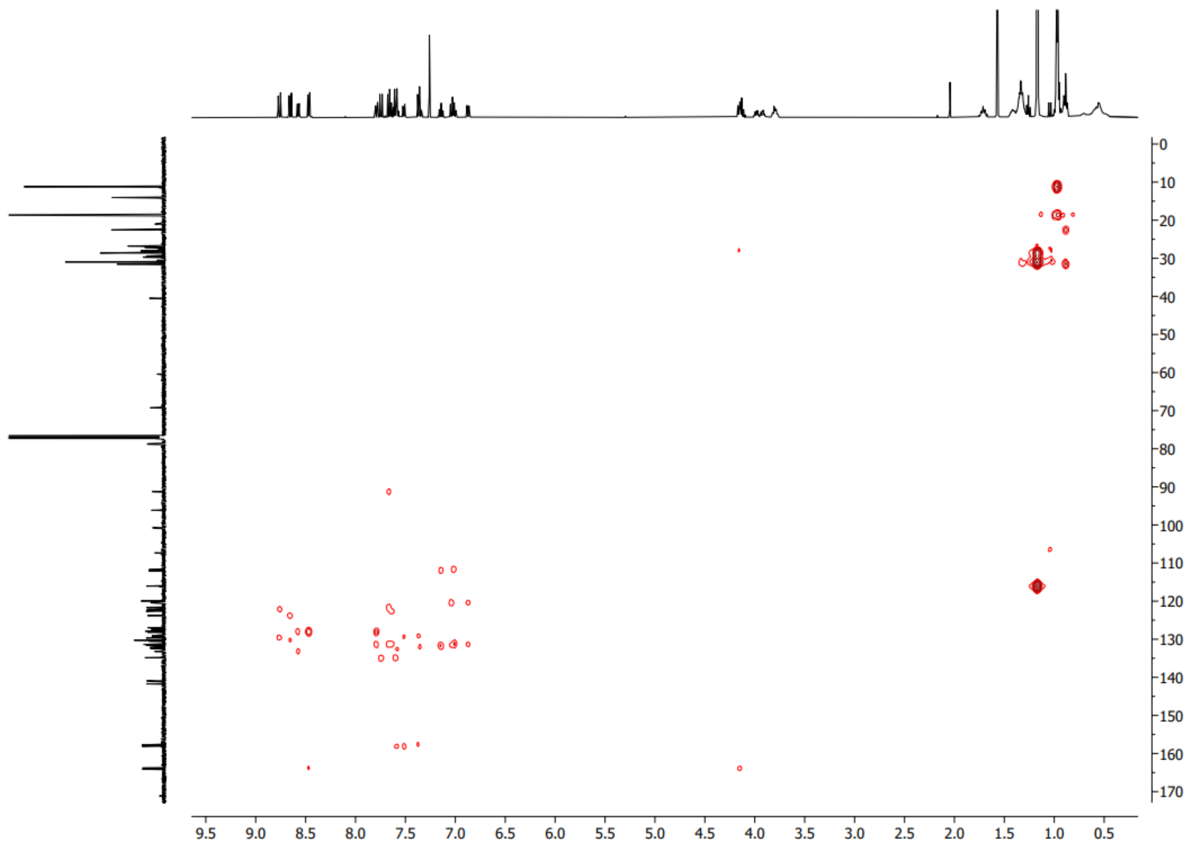

Figure S15. HMBC (500 MHz) of **AD$_8$** in $CDCl_3$, measured at 298 K.

AD$_4$

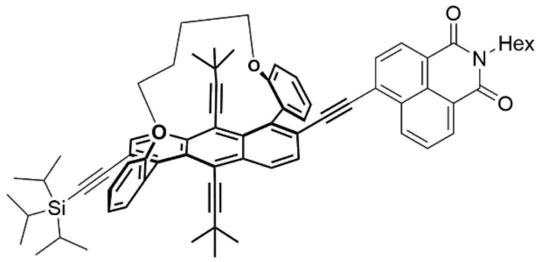

Following the general procedure **B**, 20 mg of **1-D$_4$** and 12 mg of 6-**Br-NI** were used to obtain **AD$_4$** as a yellow solid (15 mg, 55% yield).

^{1}H NMR (500 MHz, $CDCl_3$) δ 8.59 (dd, J = 7.3, 1.2 Hz, 1H), 8.51 (d, J = 7.6 Hz, 1H), 8.47 – 8.40 (m, 1H), 8.32 (s, 1H), 8.04 (ddd, J = 10.1, 7.9, 1.5 Hz, 2H), 7.96 (dd, J = 7.5, 1.7 Hz, 1H), 7.79 (d, J = 7.6 Hz, 1H), 7.71 (d, J = 9.0 Hz, 1H), 7.64 (dd, J = 8.3, 7.2 Hz, 1H), 7.57 (d, J = 9.0 Hz, 1H), 7.51 – 7.44 (m, 1H), 7.29 (dd, J = 7.7, 1.8 Hz, 1H), 7.16 (td, J = 7.5, 1.0 Hz, 1H), 7.05 (td, J = 7.5, 1.0 Hz, 1H), 6.82 – 6.74 (m, 1H), 6.66 – 6.61 (m, 1H), 4.20 – 4.08 (m, 2H), 3.72 – 3.62 (m, 2H), 3.44 – 3.35 (m, 1H), 3.34 – 3.25 (m, 1H), 1.81 – 1.67 (m, 2H), 1.42 (q, J = 7.4 Hz, 2H), 1.35 – 1.30 (m, 4H), 1.13 (s, 18H), 1.04 (s, 21H), 0.95 – 0.84 (m, 5H), 0.67 –

0.55 (m, 2H). **^{13}C NMR** (126 MHz, $CDCl_3$) δ 207.11, 164.27, 163.97, 156.44, 155.96, 140.65, 139.26, 133.33 (d, J = 4.7 Hz), 133.26, 132.50, 132.32, 132.16, 131.88, 131.58 (d, J = 4.9 Hz), 130.53 (d, J = 12.4 Hz), 130.33, 130.14, 129.57, 129.40, 129.05, 128.72 (d, J = 5.8 Hz), 128.35, 128.13, 127.21, 126.72, 125.97, 122.86, 122.56, 121.80, 120.89, 120.03 (d, J = 8.5 Hz), 119.01, 118.84, 114.25, 114.08, 110.65, 110.09, 107.61, 100.97, 96.51, 91.23, 66.39, 66.07, 40.69, 31.71, 30.93, 28.56 (d, J = 2.7 Hz), 28.22, 26.96, 26.28, 26.16, 22.72, 18.80 (d, J = 1.7 Hz), 14.21, 11.50.

HR-ESI-MS m/z (100%): 1060.56445 (100, $[M]^+$) calcd. for $C_{77}H_{85}NO_4Si^+$: 1060.56946.

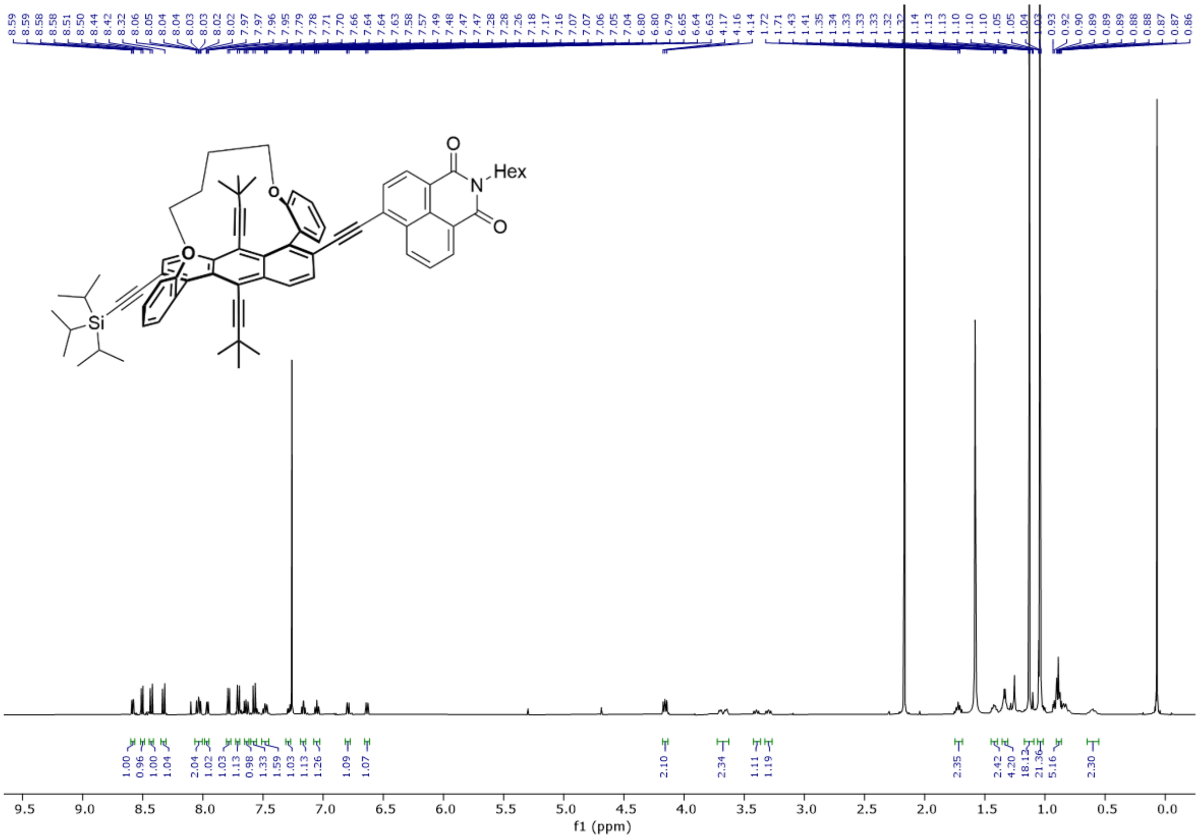

Figure S16. ^{1}H NMR (500 MHz) of **AD$_4$** in $CDCl_3$, measured at 298 K.

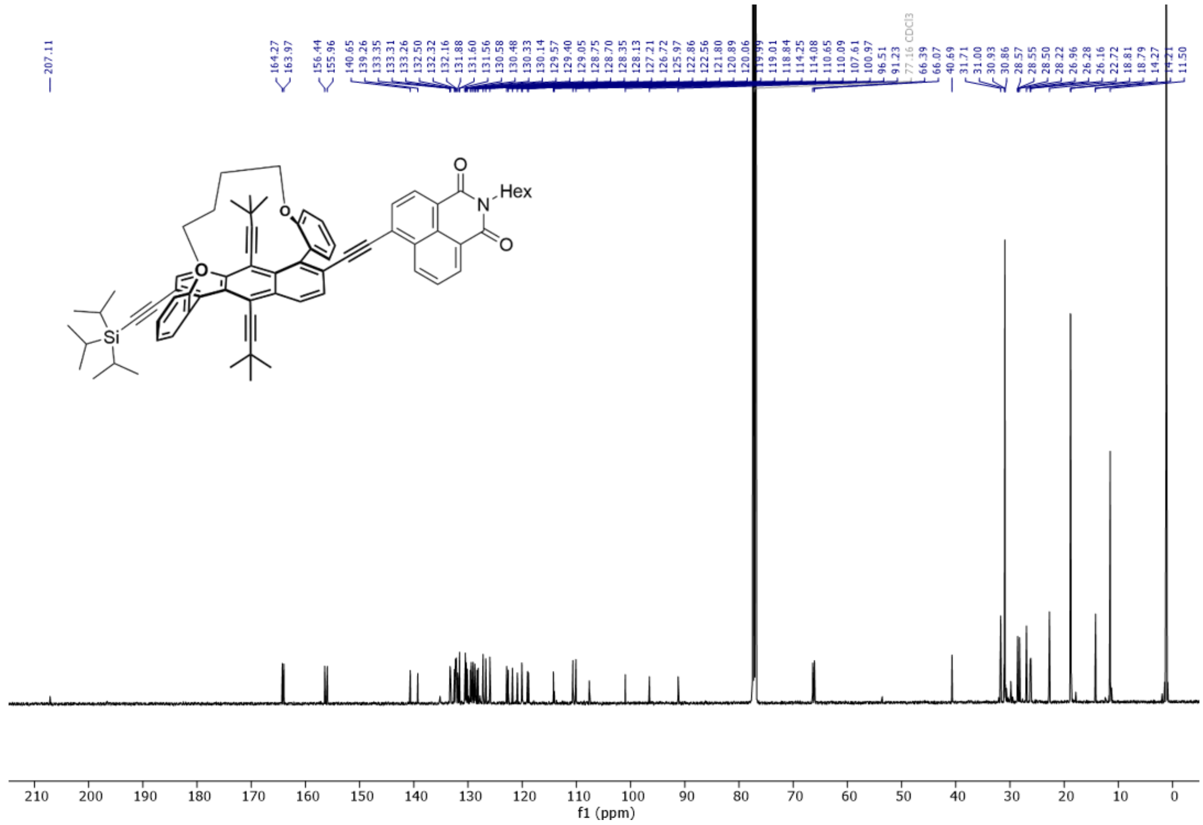

Figure S17. ^{13}C NMR (126 MHz) of **AD$_4$** in $CDCl_3$, measured at 298 K.

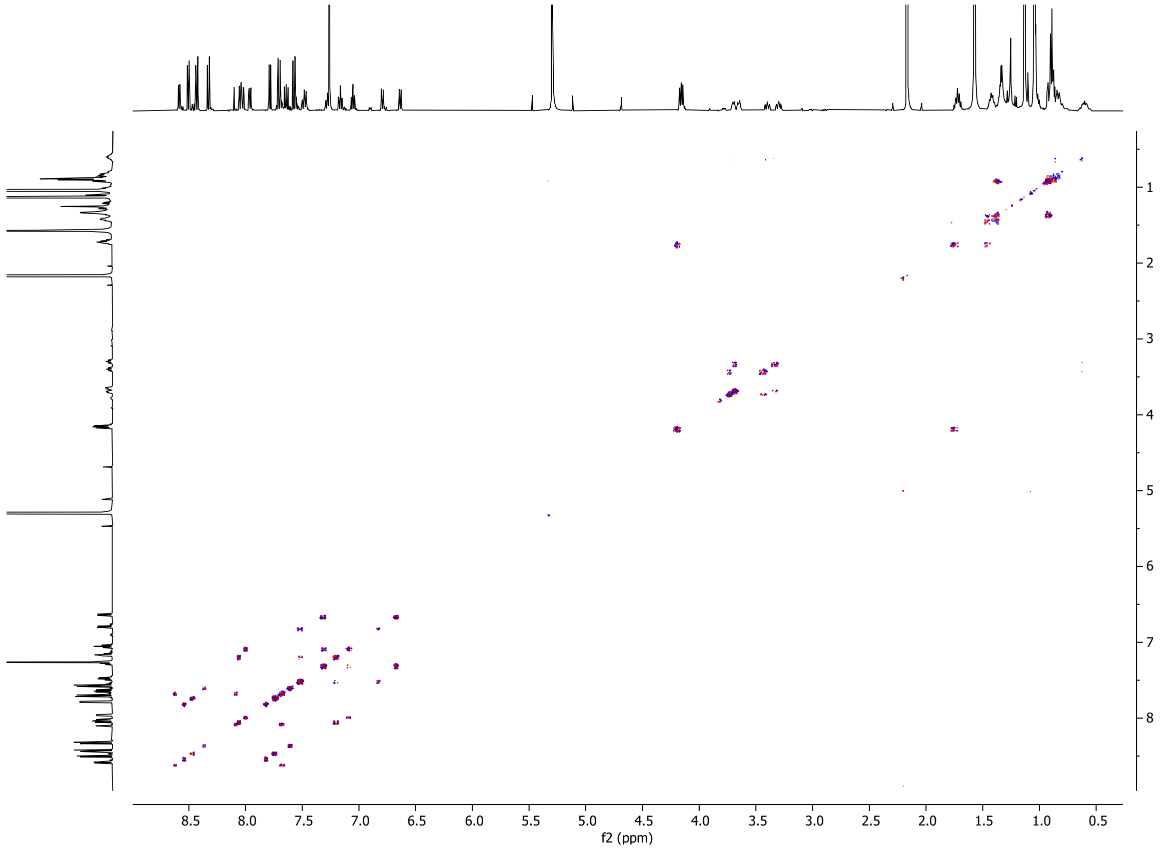

Figure S18. COSY NMR (400 MHz) of **AD$_4$** in $CDCl_3$, measured at 298 K.

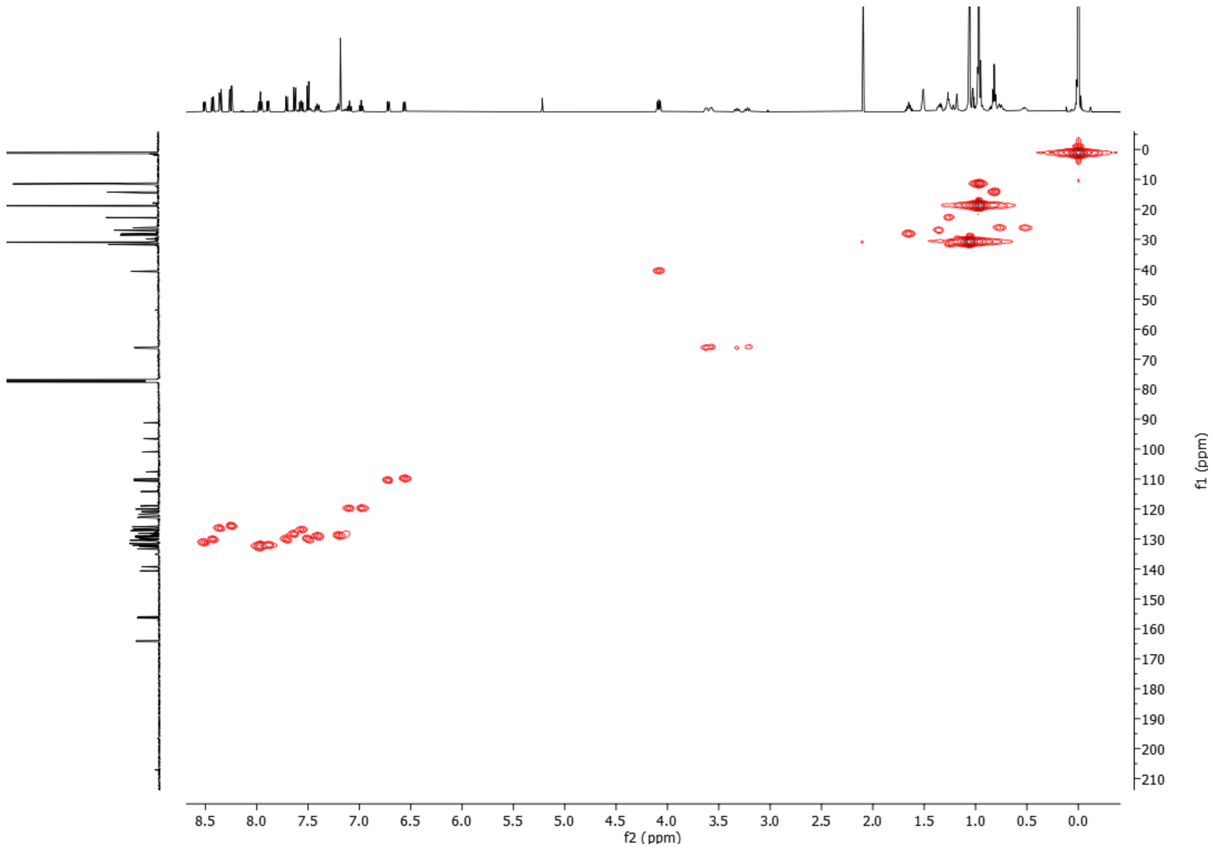

Figure S19. HSQC NMR (400 MHz) of **AD**$_4$ in $CDCl_3$, measured at 298 K.

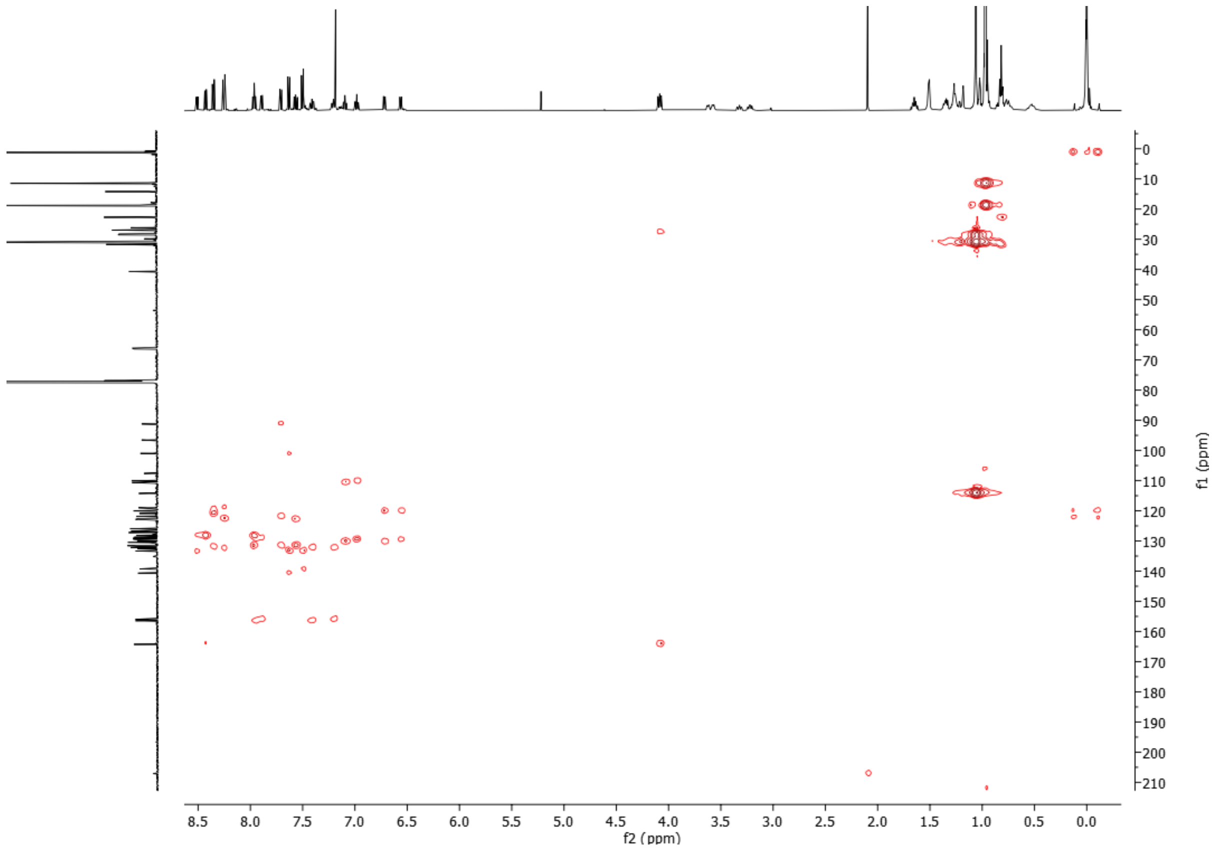

Figure S20. HMBC NMR (400 MHz) of **AD**$_4$ in $CDCl_3$, measured at 298 K.

P-AD$_4$A

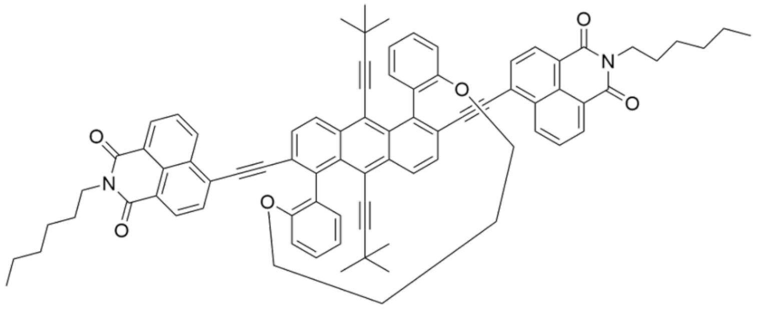

Following the general procedure **C**, 28 mg of *P*-**2-D$_4$** and 42 mg of 6-**Br-NI** were used to obtain *P*-**AD$_4$A** as an orange solid (17 mg, 33 % yield).

^{1}H NMR (400 MHz, $CDCl_3$) δ 8.59 (dd, *J* = 7.2, 1.2 Hz, 1H), 8.51 (d, *J* = 7.6 Hz, 1H), 8.45 (d, *J* = 9.0 Hz, 1H), 8.08 – 8.01 (m, 2H), 7.80 (d, *J* = 7.6 Hz, 1H), 7.75 (d, *J* = 9.0 Hz, 1H), 7.65 (dd, *J* = 8.4, 7.3 Hz, 1H), 7.54 – 7.47 (m, 1H), 7.19 (td, *J* = 7.5, 1.0 Hz, 1H), 6.81 (d, *J* = 8.2 Hz, 1H), 4.27 – 4.07 (m, 2H), 3.73 (d, *J* = 8.2 Hz, 1H), 3.47 – 3.30 (m, 1H), 1.73 (p, *J* = 7.6 Hz, 2H), 1.59 (s, 4H), 1.41 (dd, *J* = 7.8, 4.9 Hz, 1H), 1.34 (q, *J* = 3.9 Hz, 4H), 1.17 (s, 8H), 0.97 – 0.82 (m, 4H). **^{13}C NMR** (126 MHz, $CDCl_3$) δ 164.25, 163.95, 156.33, 140.60, 133.61, 133.18, 132.29, 132.20, 131.60 (d, *J* = 2.8 Hz), 130.66, 130.47, 129.95, 129.52, 129.15, 128.17 (d, *J* = 8.2 Hz), 127.26, 126.68, 122.90, 121.92, 121.35, 120.14, 119.24, 114.66, 110.63, 100.70, 91.52, 66.29, 40.71, 31.71, 31.08, 29.85, 28.64, 28.23, 26.96, 26.17, 22.72, 14.21.

MALDI-TOF m/z:1182.495 (100, $[M]^+$) calcd. for $C_{82}H_{74}N_2O_6$: 1182.554

M-AD$_8$A

Following the general procedure, 30 mg of *M*-**2-D$_8$** and 42 mg were used to obtain *M*-**AD$_8$A** as an orange solid (13 mg, 25% yield). NMR spectra of *M*-**AD$_8$A** are consistent the NMR spectra of compound *P*-**AD$_8$A**.

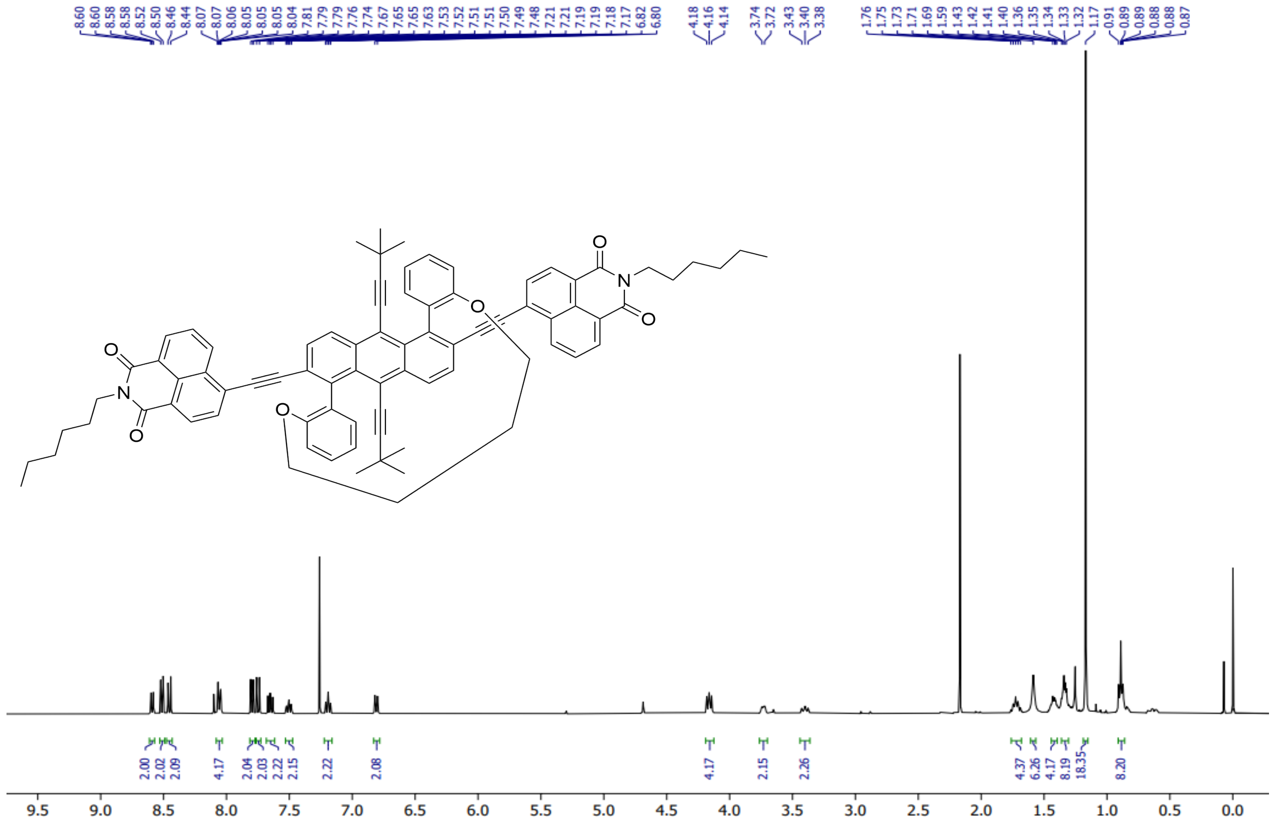

Figure S21. ^{1}H NMR (400 MHz) of *P*-**AD$_4$A** in $CDCl_3$, measured at 298 K.

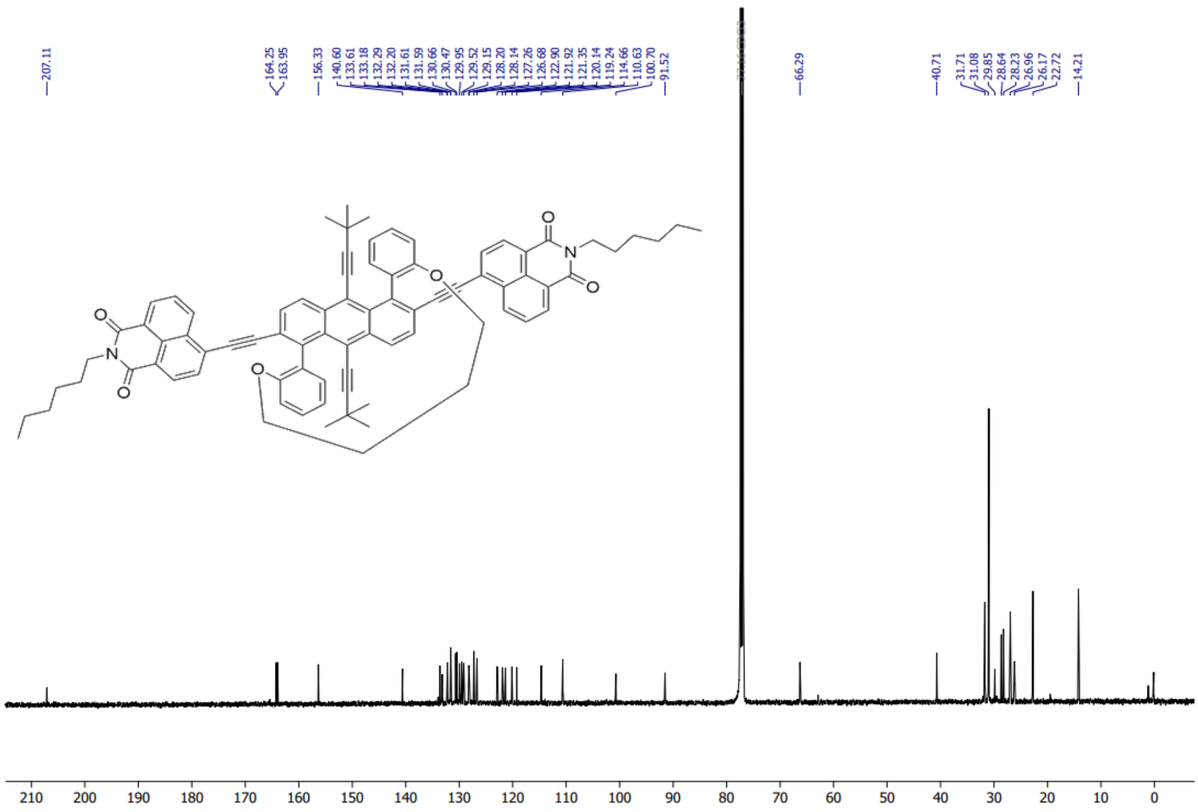

Figure S22. ^{13}C NMR (126 MHz) of *P*-**AD$_4$A** in $CDCl_3$, measured at 298 K.

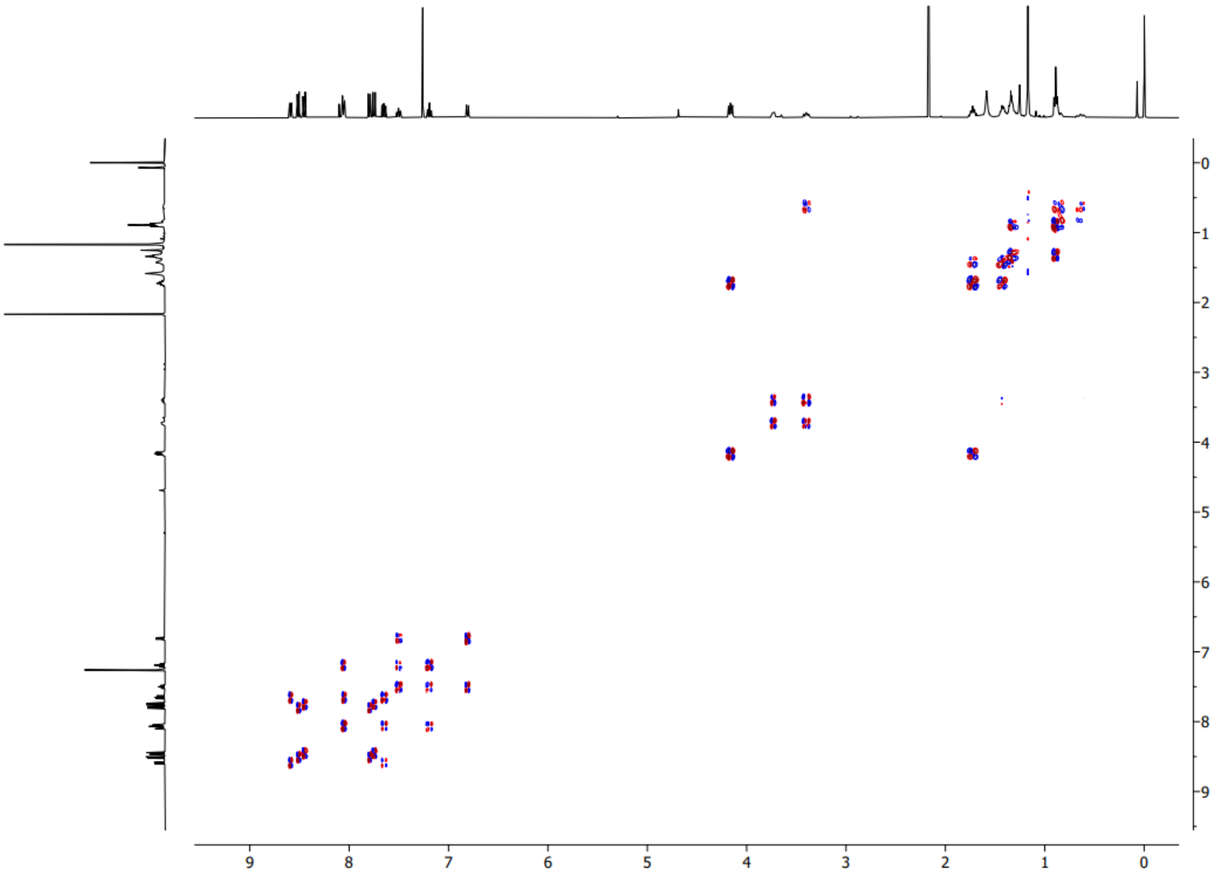

Figure S23. COSY (400 MHz) of ***P*-AD$_4$A** in $CDCl_3$, measured at 298 K.

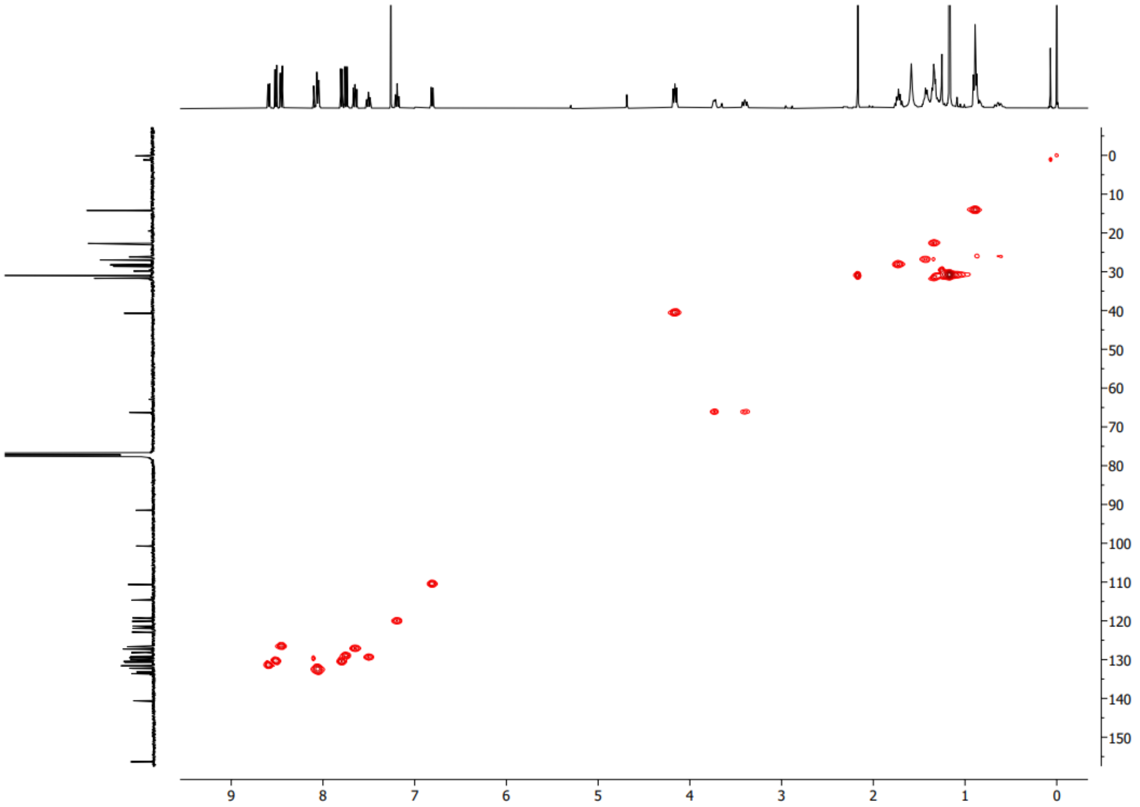

Figure S24. HSQC (400 MHz) of ***P*-AD$_4$A** in $CDCl_3$, measured at 298 K.

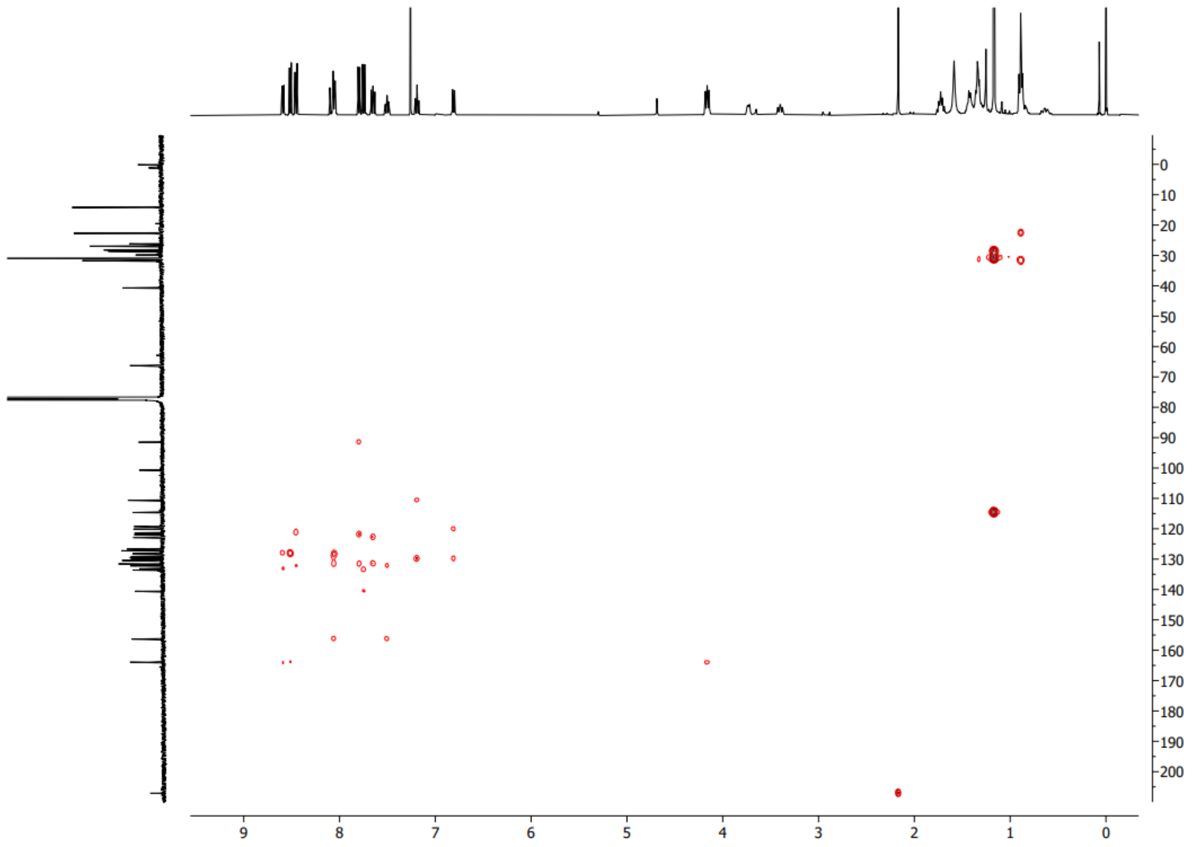

Figure S25. HMBC (400 MHz) of *P*-**AD$_4$A** in $CDCl_3$, measured at 298 K.

M-AD$_4$A

Following the general procedure **C**, 28 mg of *M*-**2-D$_4$** and 42 mg of 6-**Br-NI** were used to obtain *M*-**AD$_4$A** as a orange solid (14 mg, 30% yield). NMR spectra of *M*-**AD$_4$A** are consistent the NMR spectra of compound *P*-**AD$_4$A**.

P-AD$_8$A

Following the general procedure **(C)**, 30 mg of *P*-**2-D$_8$** and 42 mg of 6-**Br-NI** were used to obtain *P*-**AD$_8$A** as an orange solid (**15** mg, 28 % yield).

^{1}H NMR (500 MHz, $CDCl_3$) δ 8.80 (d, *J* = 9.2 Hz, 2H), 8.59 (dd, *J* = 7.2, 1.2 Hz, 2H), 8.48 (d, *J* = 7.6 Hz, 2H), 7.81 (dd, *J* = 1.2 Hz, 2H), 7.79 (d, *J* = 9.2 Hz, 2H), 7.67 (t, *J* = 7.4 Hz, 3H), 7.65 (d, *J* = 7.4 Hz, 1H), 7.61 (ddd, *J* = 8.3, 7.5, 1.7 Hz, 2H), 7.53 (dd, *J* = 7.4, 1.7 Hz, 2H), 7.17 (td, *J* = 7.4, 1.0 Hz, 2H), 7.06 (dd, *J* = 8.3, 1.1 Hz, 2H), 4.19 – 4.11 (m, 4H), 3.99 (dt, *J* = 8.8, 4.2 Hz, 2H), 3.87 – 3.81 (m, 2H), 1.72 (p, *J* = 7.7 Hz, 4H), 1.45 – 1.39 (m, 4H), 1.37 – 1.30 (m, 12H), 1.21 (s, 18H), 0.92 – 0.84 (m, 6H), 0.69 (m, 2H), 0.57 (m, 6H). **^{13}C NMR** (126

MHz, CDCl3) δ 164.27, 163.96, 158.29, 141.94, 135.28, 133.30, 132.56, 131.67, 131.55, 130.78, 130.44, 130.24, 129.58, 128.88, 128.21, 128.08, 127.18, 122.84, 122.76, 121.89, 120.70, 120.44, 116.79, 112.30, 100.62, 91.69, 78.86, 69.57, 40.68, 31.71, 31.10, 29.82, 29.45, 28.85, 28.23, 27.15, 26.95, 22.72, 14.21.

MALDI-TOF m/z:1238.617 (100, $[M]^+$) calcd. for $C_{86}H_{82}N_2O_6$: 1238.784.

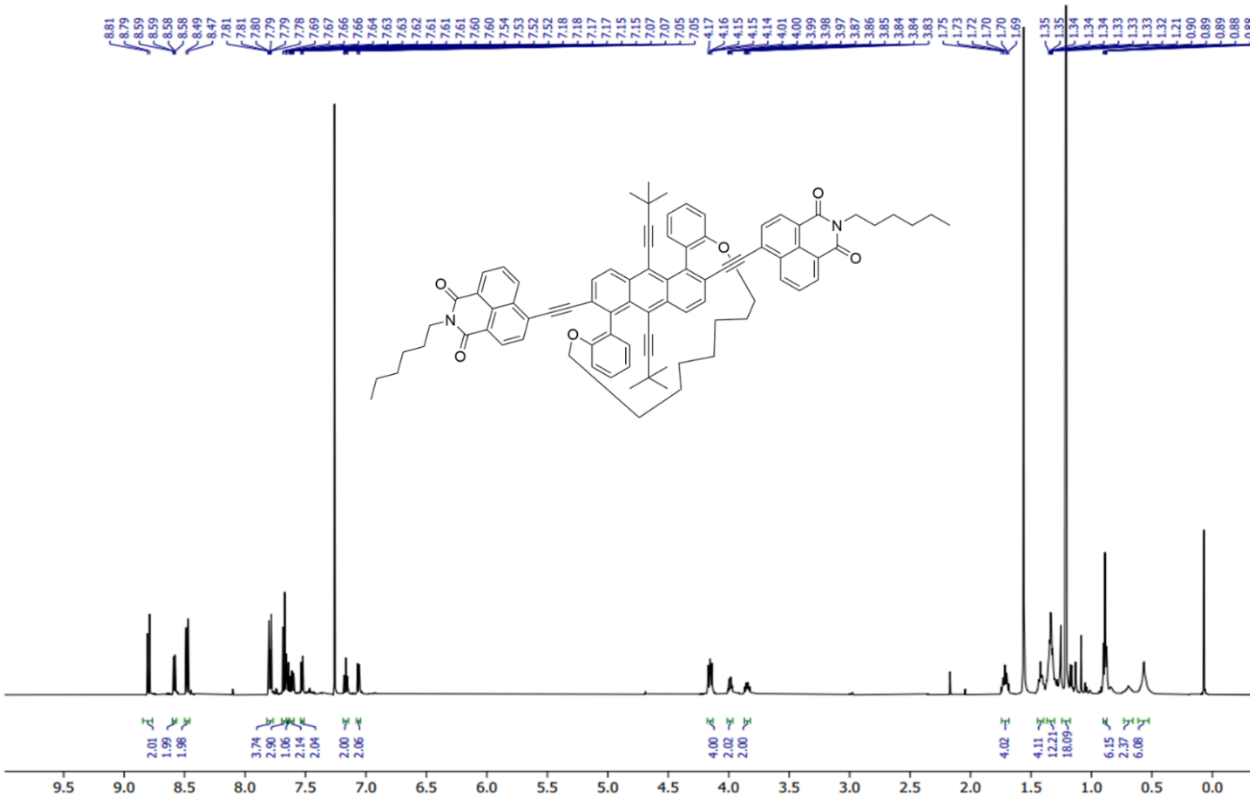

Figure S26. ^{1}H NMR (126 MHz) of *P*-**AD$_8$A** in $CDCl_3$, measured at 298 K.

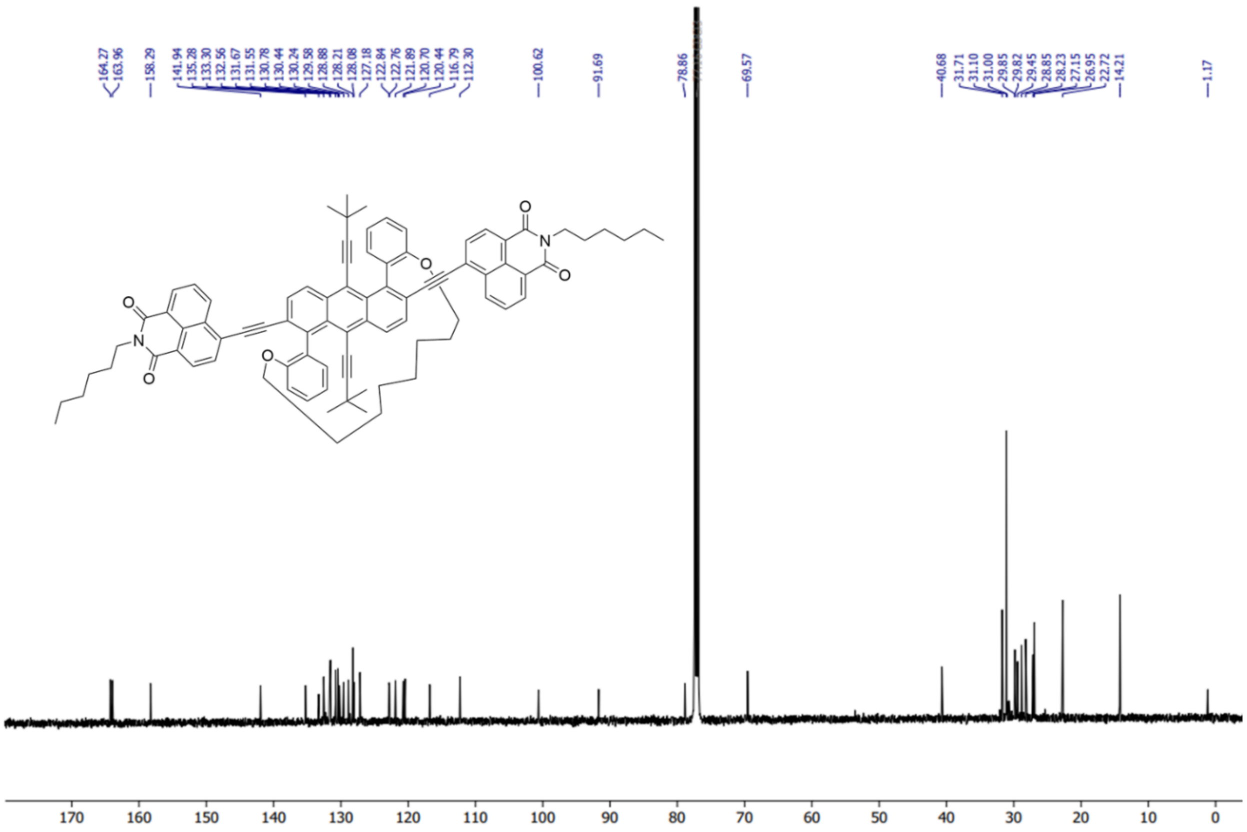

Figure S27. ^{13}C NMR (126 MHz) of *P*-**AD$_8$A** in $CDCl_3$, measured at 298 K.

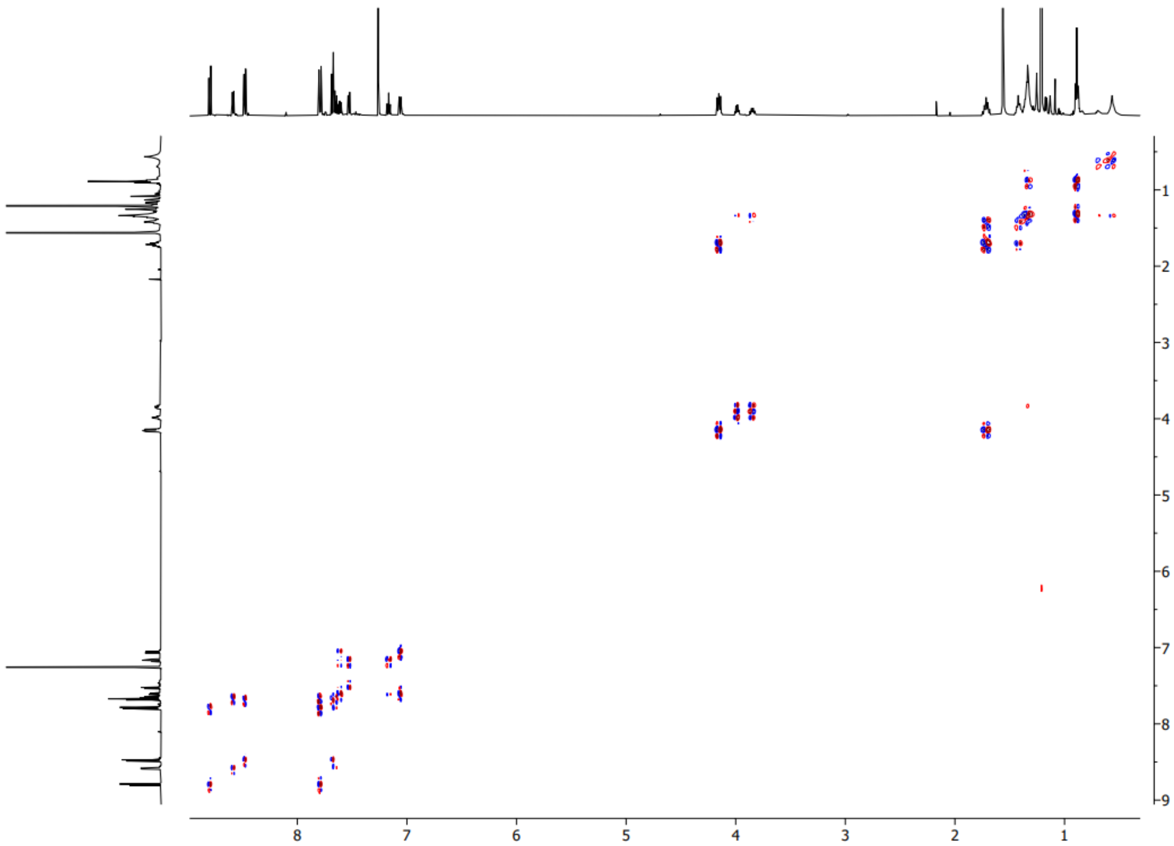

Figure S28. COSY (500 MHz) of *P*-**AD$_8$A** in $CDCl_3$, measured at 298 K.

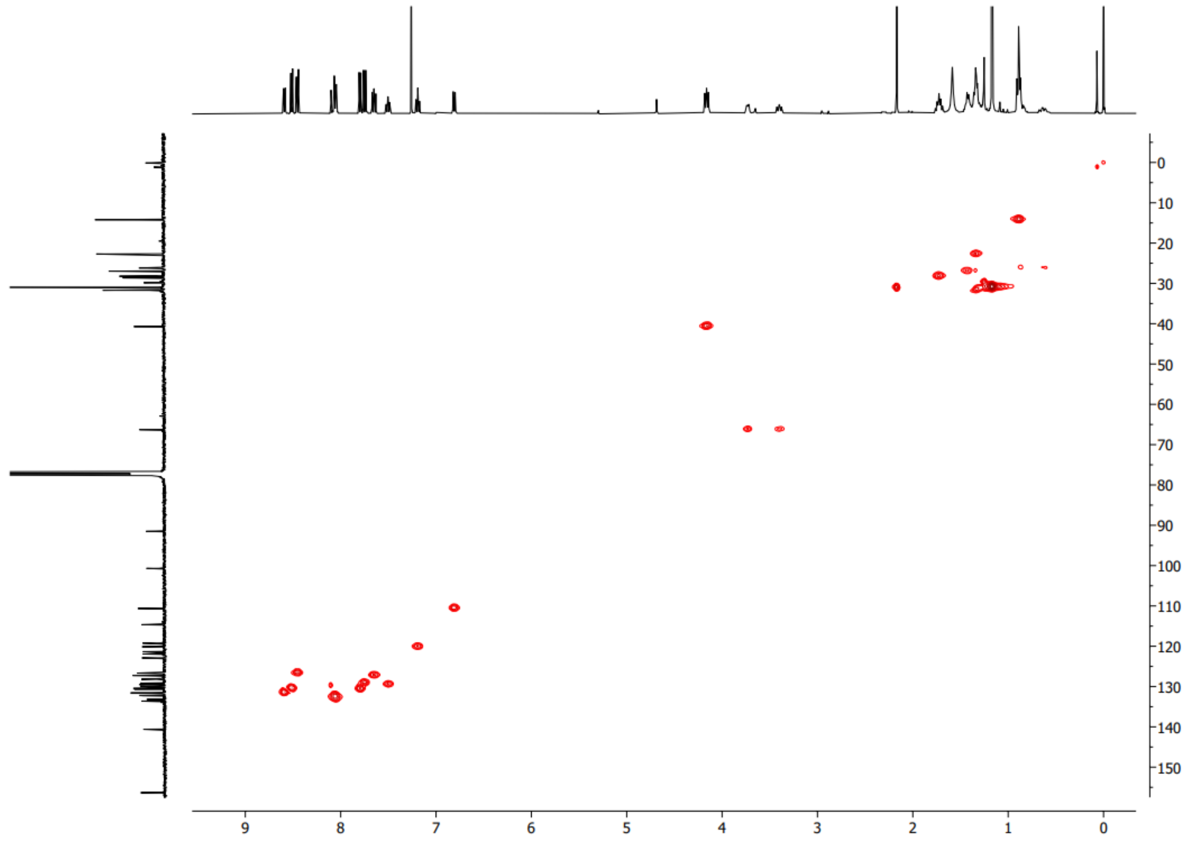

Figure S29. HSQC (500 MHz) of *P*-$\mathbf{AD_8A}$ in $CDCl_3$, measured at 298 K.

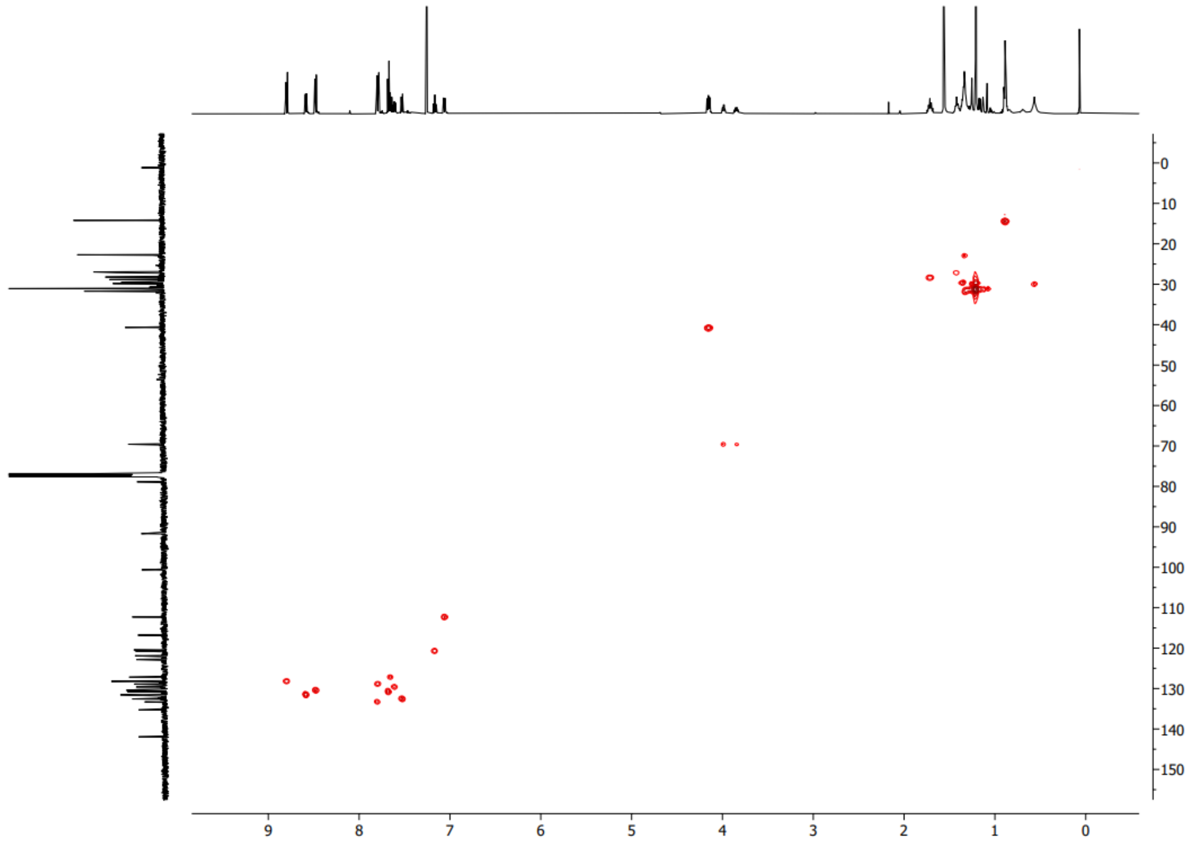

Figure S30. HMBC (500 MHz) of *P*-$\mathbf{AD_8A}$ in $CDCl_3$, measured at 298 K.

S3.2 Mass spectroscopy

S3.2.1 MALDI

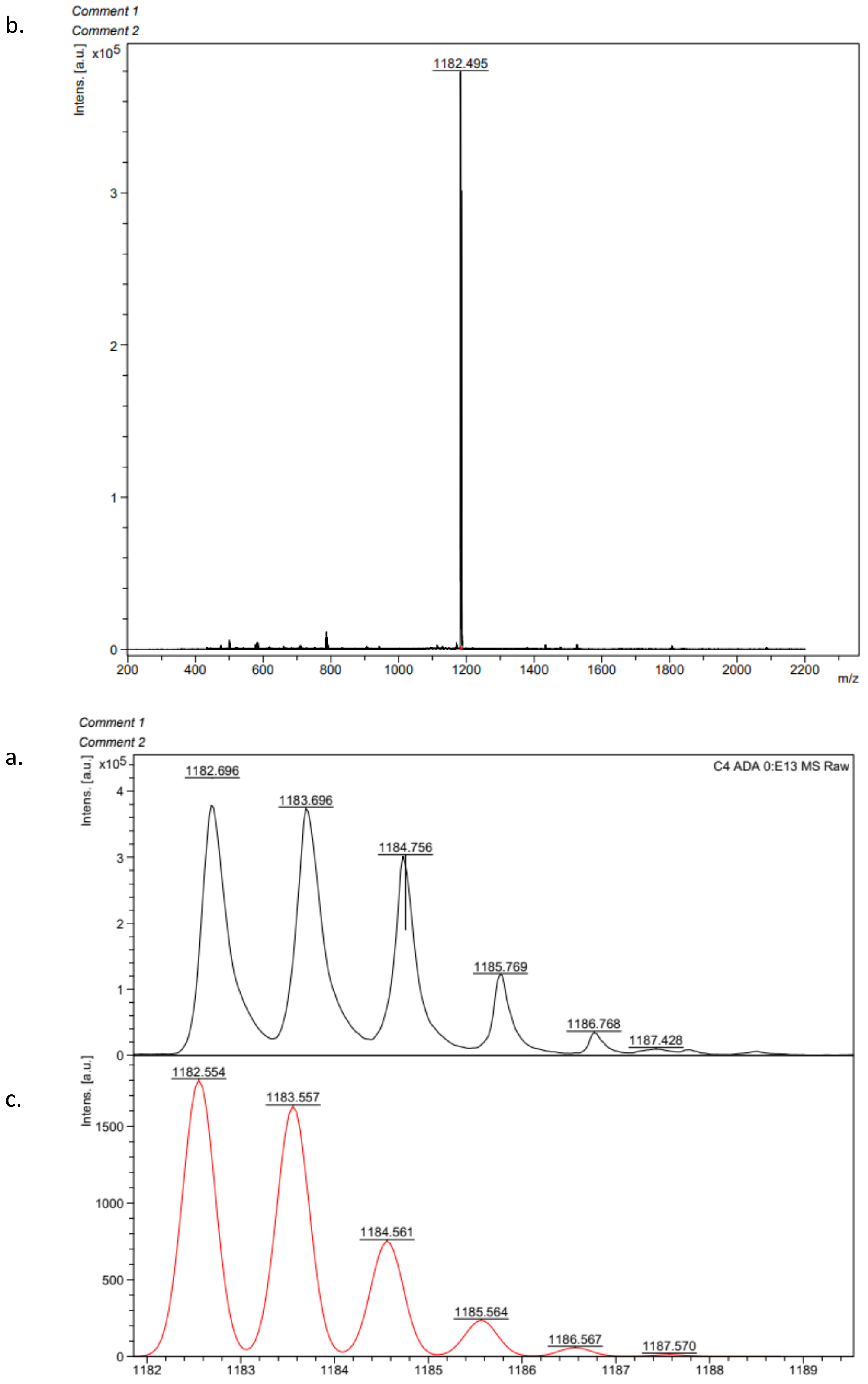

Figure S31. MALDI-TOF spectrum of *P*-**AD$_4$A**. a. full spectrum, b. experimental spectrum, c. theoretical spectrum.

a.

b.

c.

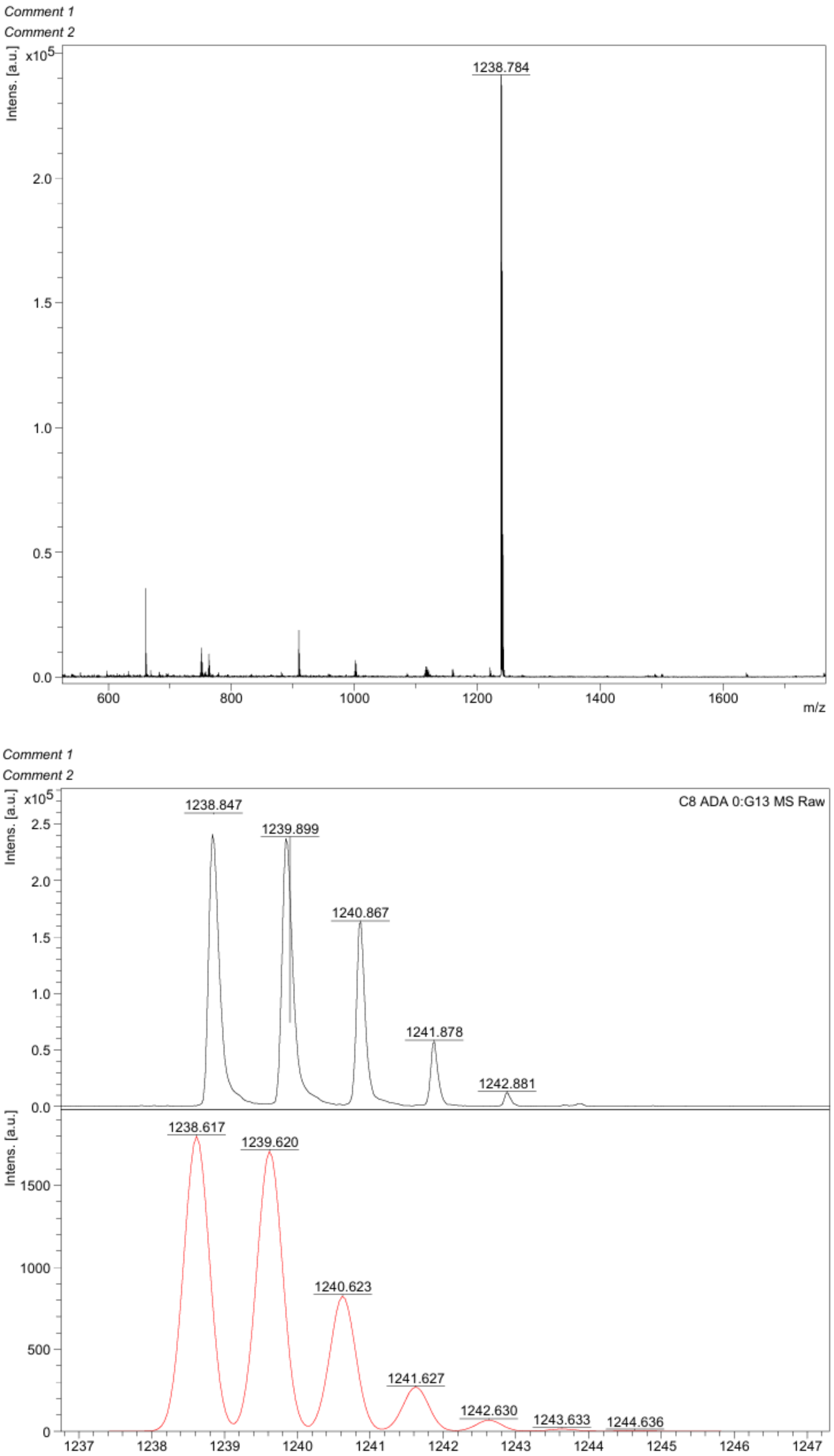

Figure S32. MALDI-TOF spectrum of *P*-**AD$_8$A**. a. full spectrum, b. experimental spectrum, c. theoretical spectrum.

S4 Photophysical and chiroptical properties

S4.1 Electronic absorption spectra

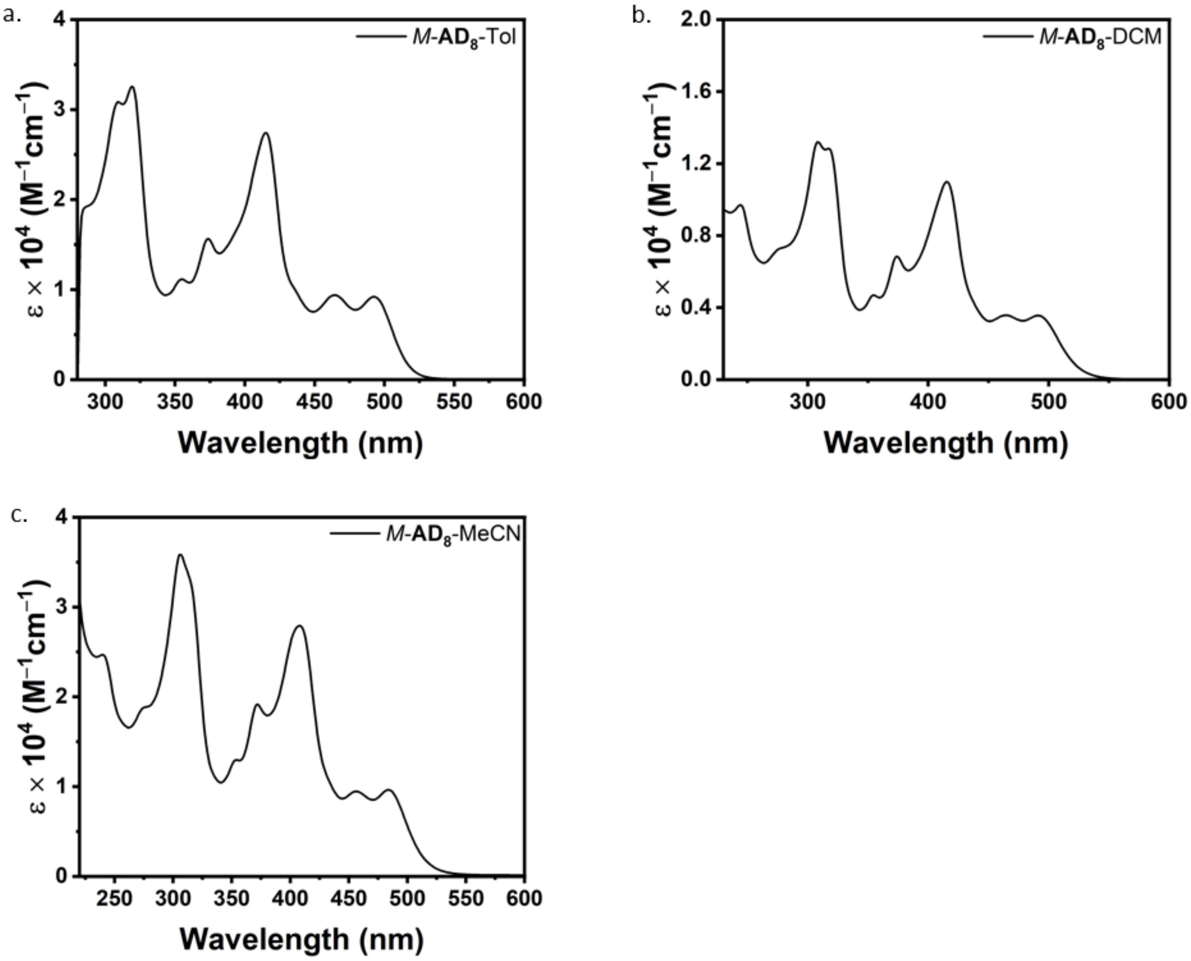

Figure S33. Electronic absorption spectra of *M*-**AD**$_8$ in different solvents (a) toluene (Tol) (b) DCM (c) acetonitrile (MeCN) measured at 298 K.

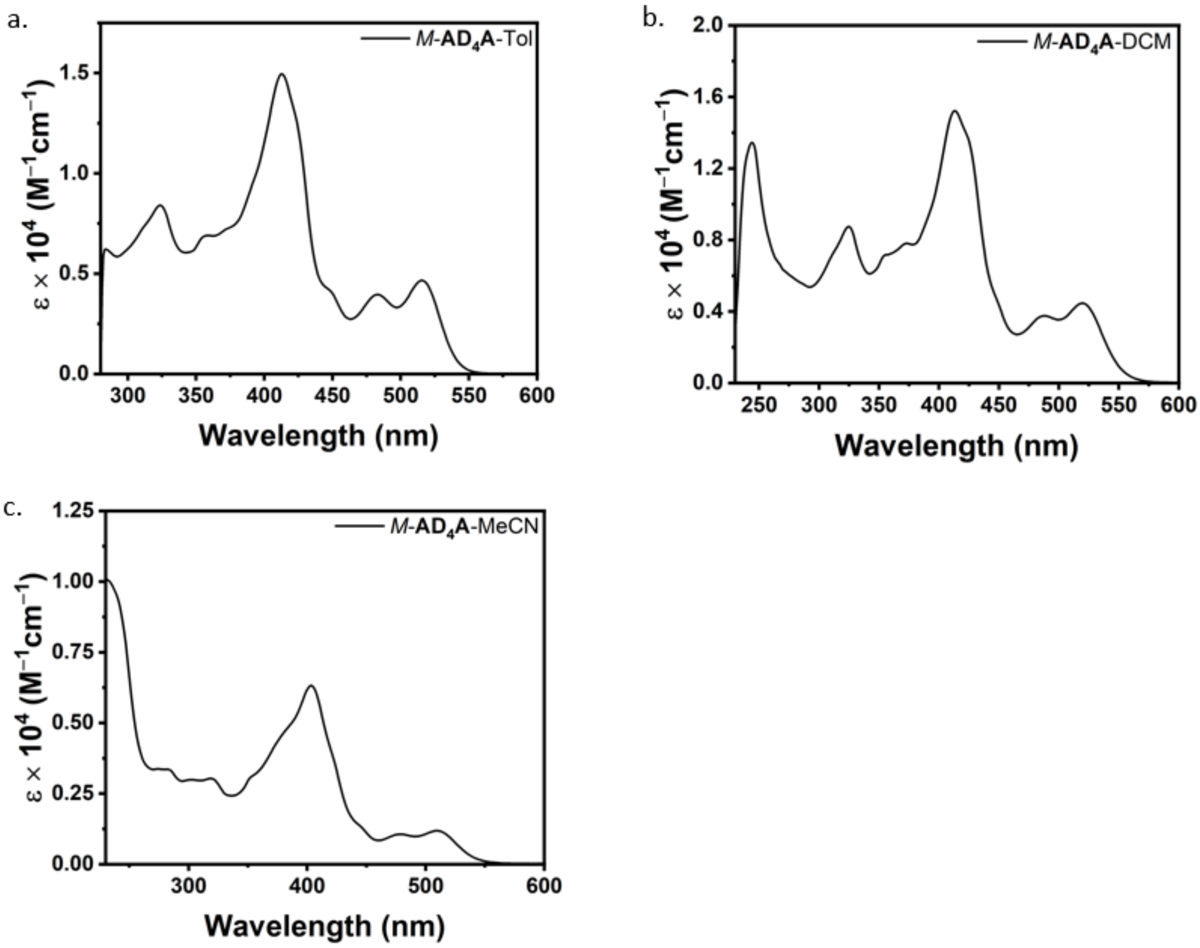

Figure S34. Electronic absorption spectra of *M*-**AD$_4$A** in different solvents (a) toluene (Tol) (b) DCM (c) acetonitrile (MeCN) measured at 298 K.

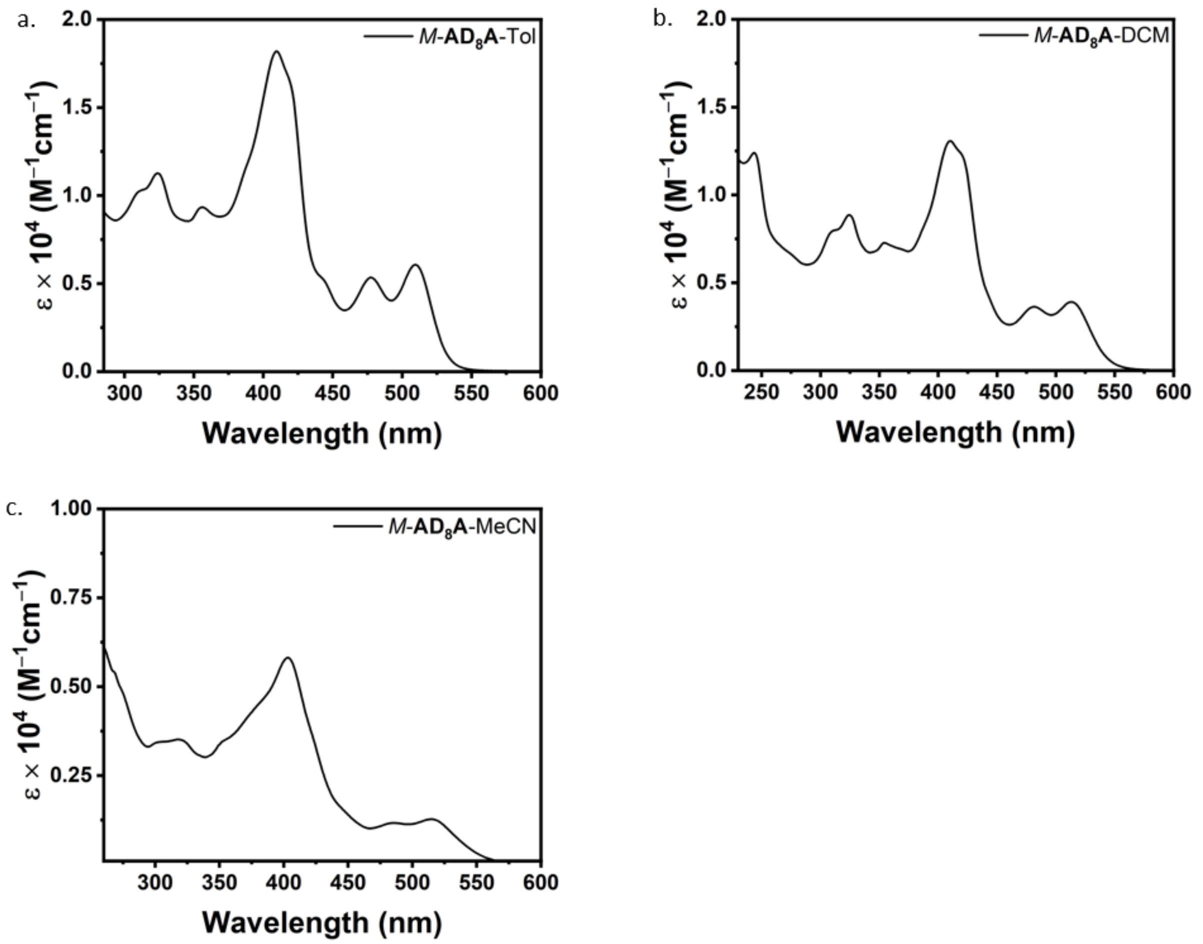

Figure S35. Electronic absorption spectra of *M*-**AD**$_8$**A** in different solvents (a) toluene (Tol) (b) DCM (c) acetonitrile (MeCN) measured at 298 K.

S4.2 ECD spectra

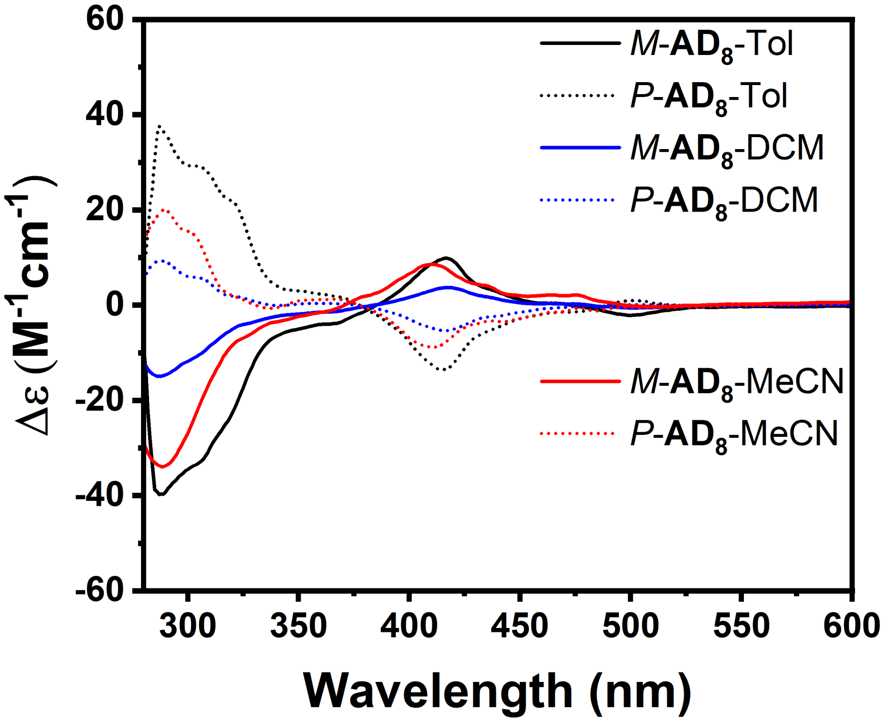

Figure S36. ECD spectra of *M*-**AD8** and *P*-**AD8** in toluene (Tol), DCM, acetonitrile (MeCN) measured at 298 K.

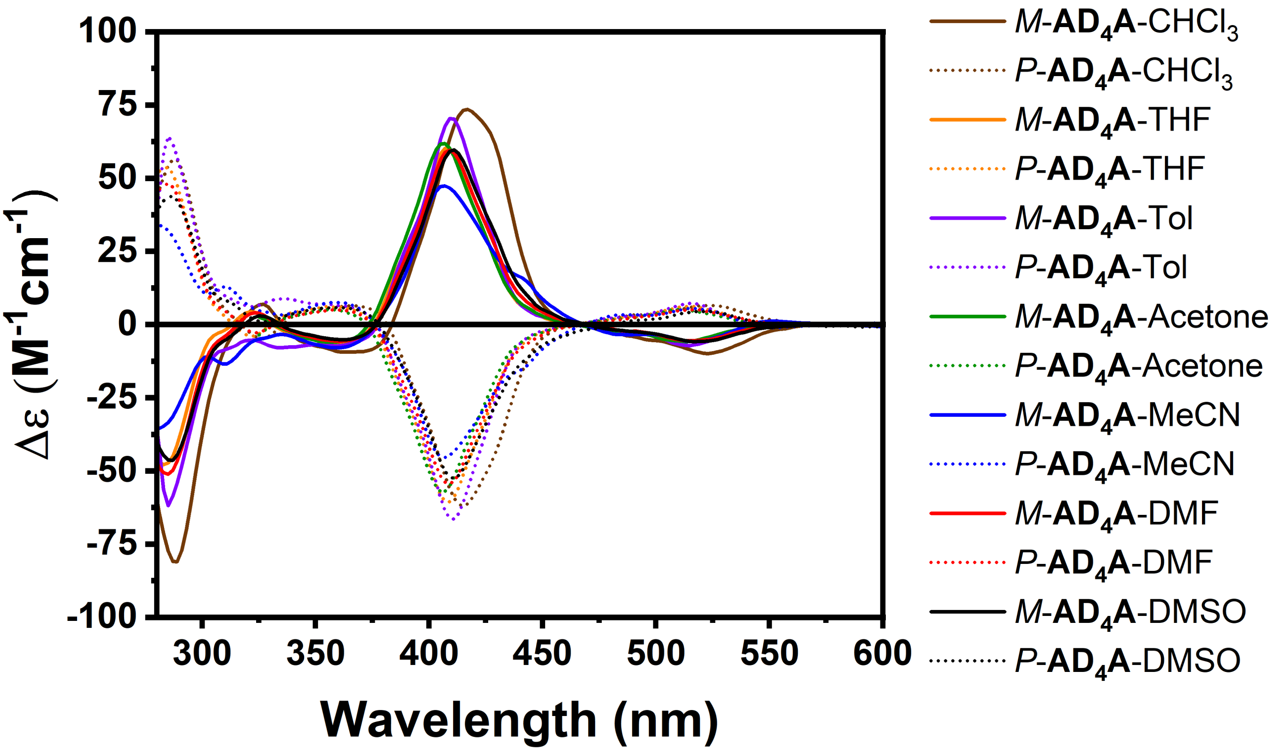

Figure S37. ECD spectra of *M*-**AD4A** and *P*-**AD4A** in chloroform ($CHCl_3$), THF, toluene (Tol), Acetone, acetonitrile (MeCN), DMF, and DMSO measured at 298 K.

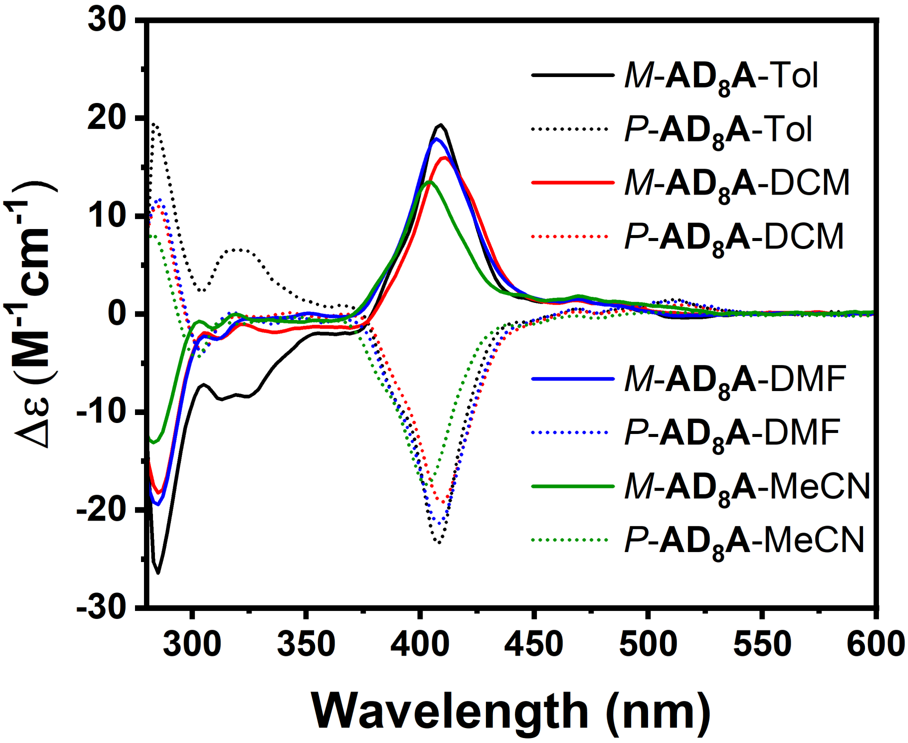

Figure S38. ECD spectra of *M*-**AD**$_8$**A** and *P*-**AD**$_8$**A** in toluene (Tol), DCM, DMF acetonitrile (MeCN) measured at 298 K.

S4.3 Stationary fluorescence spectra

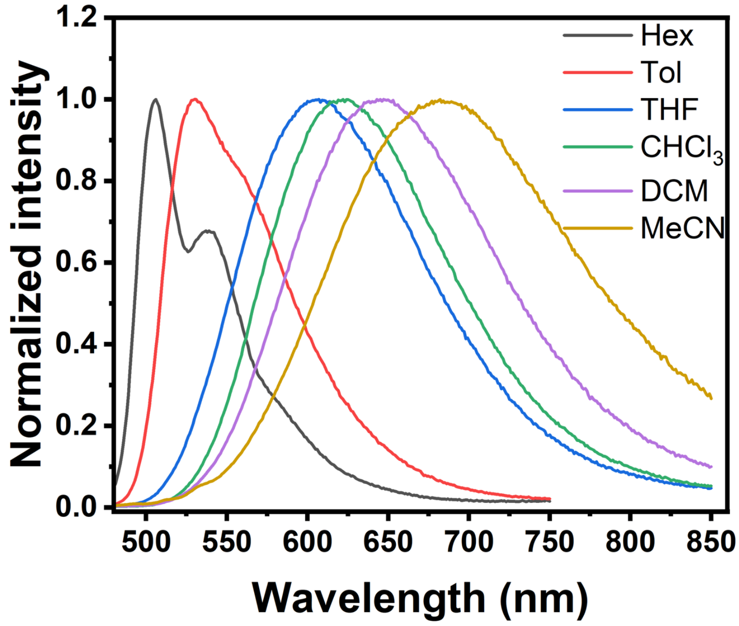

Figure S39. Stationary fluorescence spectra of **AD**$_8$ in hexane (Hex), toluene (Tol), THF, chloroform ($CHCl_3$), DCM, and acetonitrile (MeCN) measured at 298 K.

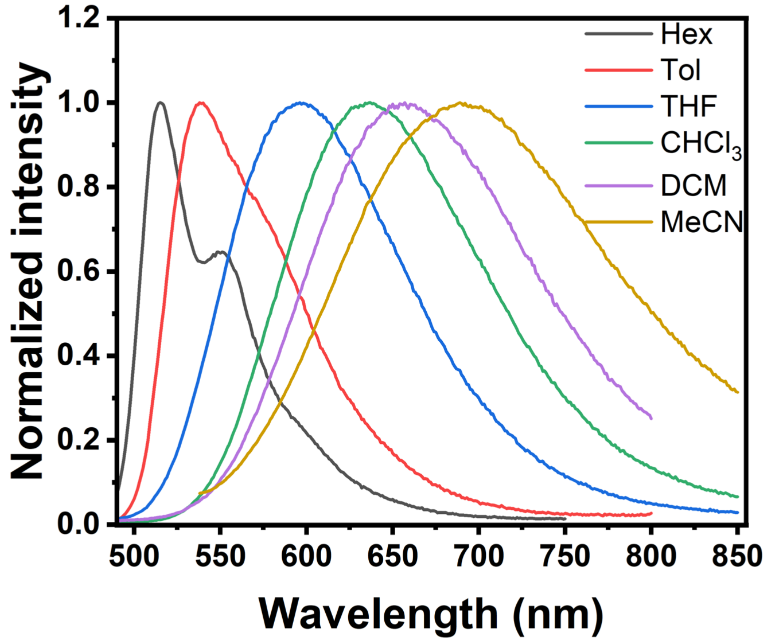

Figure S40. Stationary fluorescence spectra of $\mathbf{AD_4}$ in hexane (Hex), toluene (Tol), THF, chloroform ($CHCl_3$), DCM, and acetonitrile (MeCN) measured at 298 K.

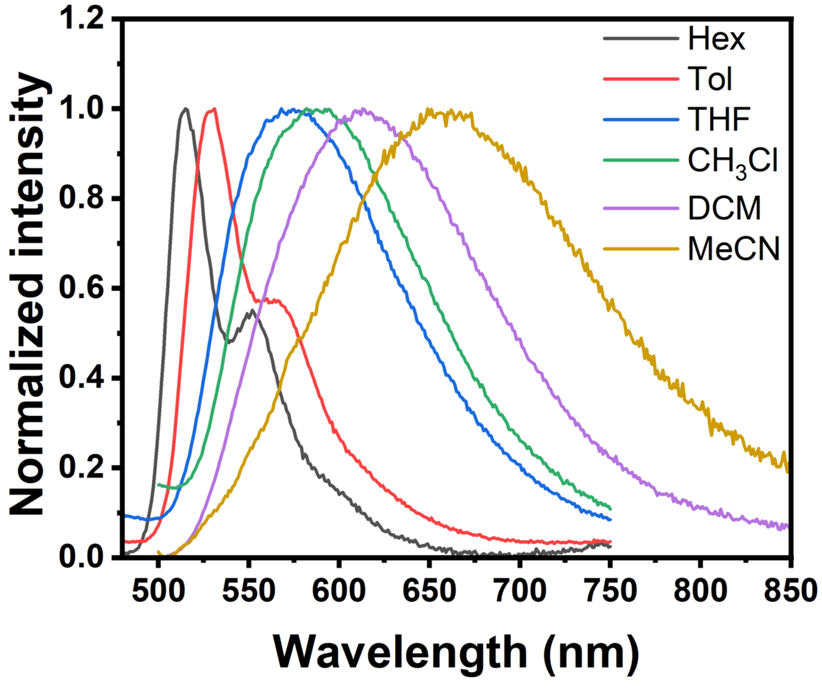

Figure S41. Stationary fluorescence spectra of $\mathbf{AD_8A}$ in hexane (Hex), toluene (Tol), THF, chloroform ($CHCl_3$), DCM, and acetonitrile (MeCN) measured at 298 K.

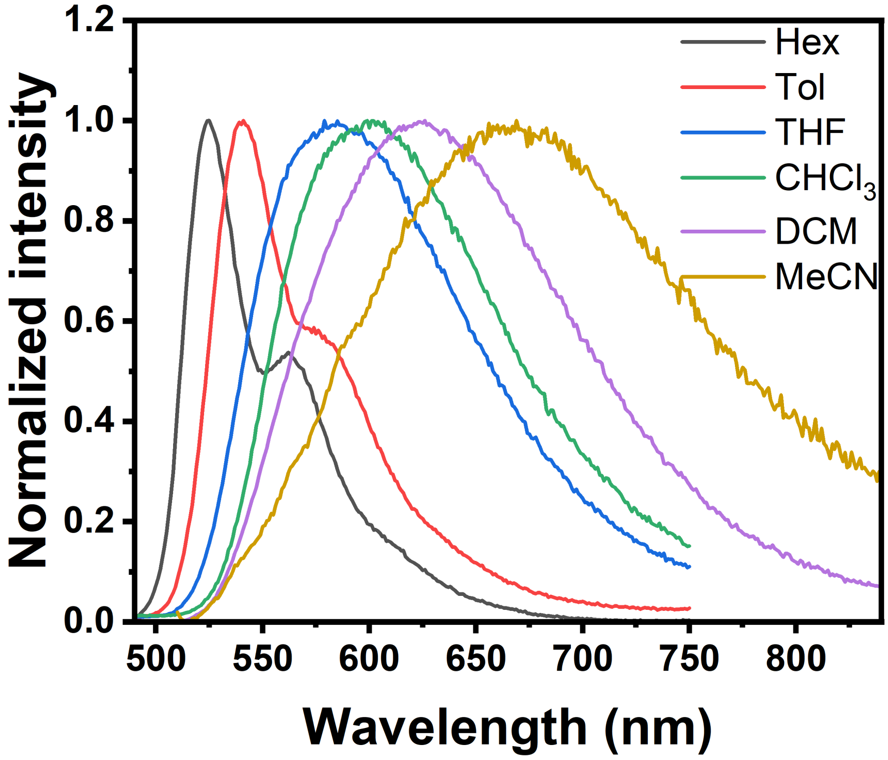

Figure S42. Stationary fluorescence spectra of **AD$_4$A** in hexane (Hex), toluene (Tol), THF, chloroform ($CHCl_3$), DCM, and acetonitrile (MeCN) measured at 298 K.

S4.4 Solvatochromism

The orientation polarizability, Δf, was calculated as:

$$\Delta f = f(\epsilon) - f(n^2)$$

with $$f(x) = \frac{2(x-1)}{(2x+1)}$$

and x=ϵ or n, where is ϵ and n are the dielectric constant and the refractive index of the solvent.

Table S1. Dielectric properties of the solvents

	**Solvent**	ϵ	n	n^2	Δf
1	Hexane	1.89	1.37	1.89	0
2	Toluene	2.38	1.49	2.24	0.026
3	THF	7.52	1.41	1.98	0.419
4	Chloroform	4.81	1.44	2.07	0.296
5	DCM	8.93	1.42	2.03	0.434
6	Acetonitrile	36.64	1.34	1.81	0.61

Table S2. Solvatochromism of **AD_4A**

	**Solvent**	**λ_{abs} / nm**	**E_{abs} / cm^{-1}**	**λ_{em} / nm**	**E_{em} / cm^{-1}**
1	Hexane	510	19608	525	19048
2	Toluene	515	19417	541	18484
3	THF	515	19417	585	17094
4	Chloroform	524	19084	599	16694
5	DCM	520	19231	626	15974
6	Acetonitrile	509	19646	669	14948

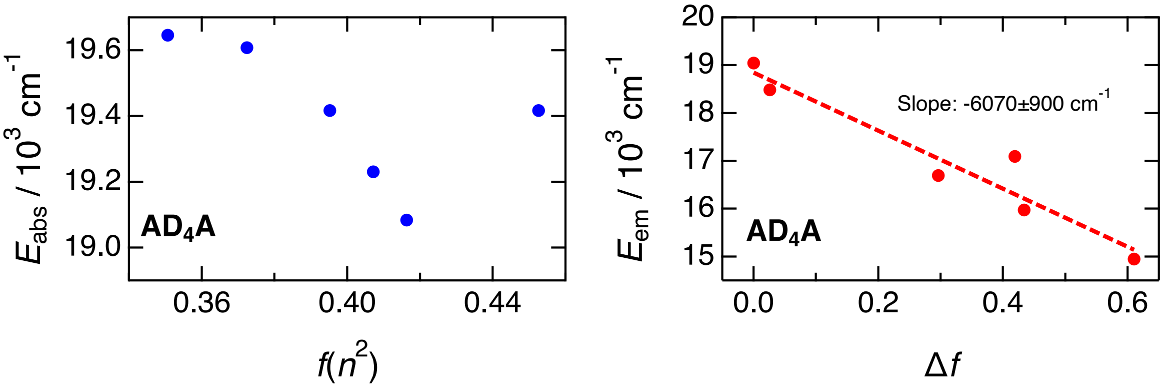

Figure S43. Absorption and emission solvatochromism **AD4A**.

Table S3. Solvatochromism of **AD8A**

	Solvent	λ_{abs} / nm	E_{abs} / cm^{-1}	λ_{em} / nm	E_{em} / cm^{-1}
1	**Hexane**	503	19881	515	19417
2	**Toluene**	509	19646	531	18832
3	**THF**	509	19646	574	17422
4	**Chloroform**	518	19305	592	16892
5	**DCM**	514	19455	613	16313
6	**Acetonitrile**	504	19841	660	15152

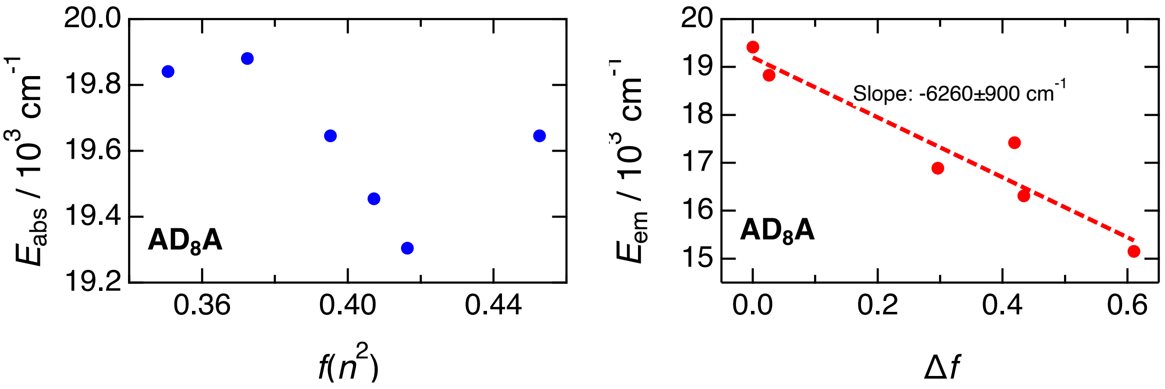

Figure S44. Absorption and emission solvatochromism **AD8A**.

Table S4. Solvatochromism of **AD_4**

	Solvent	λ_{abs} / nm	E_{abs} / cm^{-1}	λ_{em} / nm	E_{em} / cm^{-1}
1	**Hexane**	497	20121	515	19417
2	**Toluene**	502	19920	538	18587
3	**THF**	497	20121	596	16779
4	**Chloroform**	506	19763	637	15699
5	**DCM**	500	20000	657	15221
6	**Acetonitrile**	493	20284	689	14514

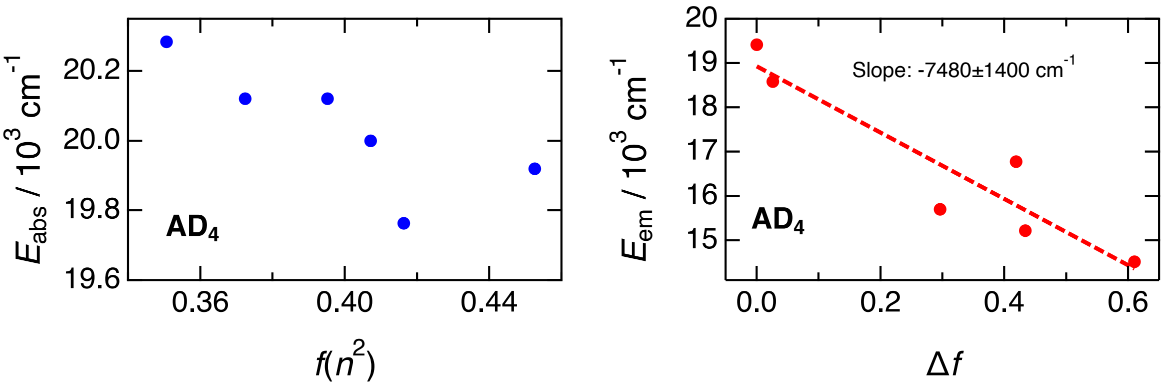

Figure S45. Absorption and emission solvatochromism of **AD_4**

Table S5. Solvatochromism of **AD₈**

	Solvent	λ_{abs} / nm	E_{abs} / cm^{-1}	λ_{em} / nm	E_{em} / cm^{-1}
1	**Hexane**	492	20325	506	19763
2	**Toluene**	496	20161	530	18868
3	**THF**	491	20408	607	16474
4	**Chloroform**	500	20000	622	16077
5	**DCM**	495	20202	643	15552
6	**Acetonitrile**	487	20534	682	14663

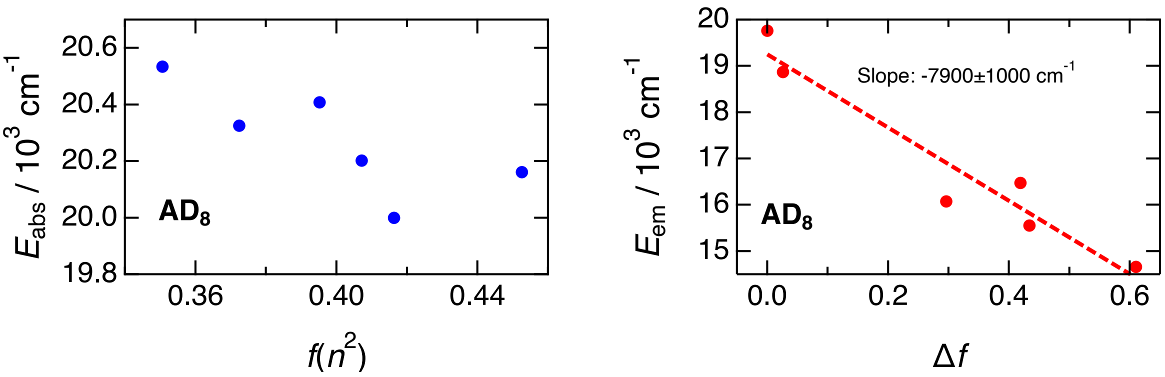

Figure S46. Absorption and emission solvatochromism of **AD$_8$**

S4.5 Fluorescence excitation spectra

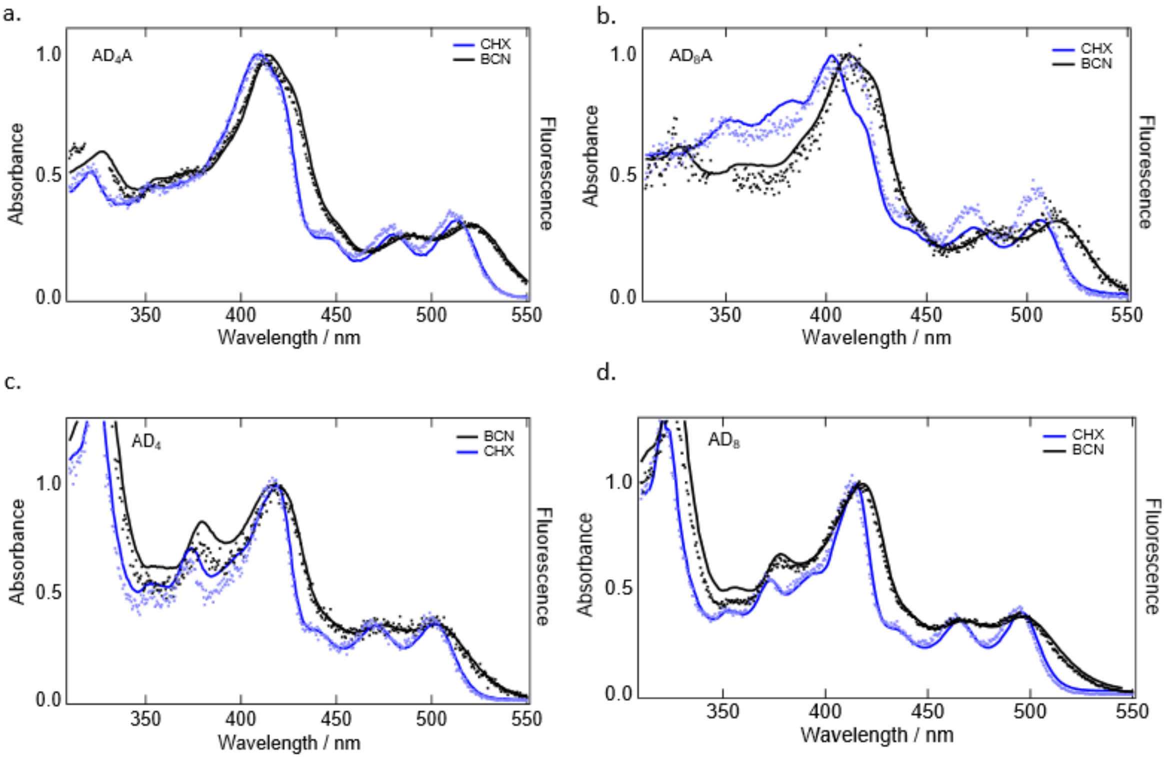

Figure S47. Absorption (solid line) and fluorescence excitation spectra (dotted line) measured for (a) **AD$_4$A**, (b) **AD$_8$A** (c), **AD$_4$** and (d) **AD$_8$** molecules in cyclohexane (CHX) and benzonitrile (BCN).

S4.6 Fluorescence quantum yields

Table S2. Fluorescence quantum yield of **AD$_n$A** in different solvents

Compound	Hexane	Acetonitrile
**AD$_4$A**	0.43	---
**AD$_8$A**	0.50	0.02

S4.7 Circularly polarized luminescence (CPL)

S4.7.1 AD_4A

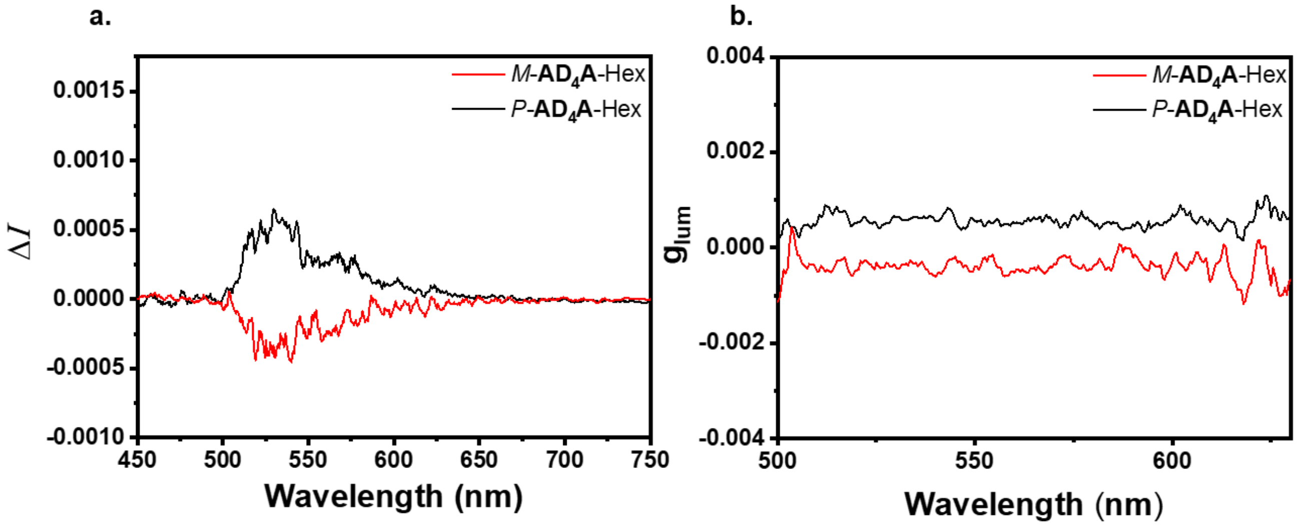

Figure S48. (a) CPL and (b) g_{lum} spectra of $\mathbf{AD_4A}$ enantiomers measured in hexane (Hex).

S4.7.2. AD_8A

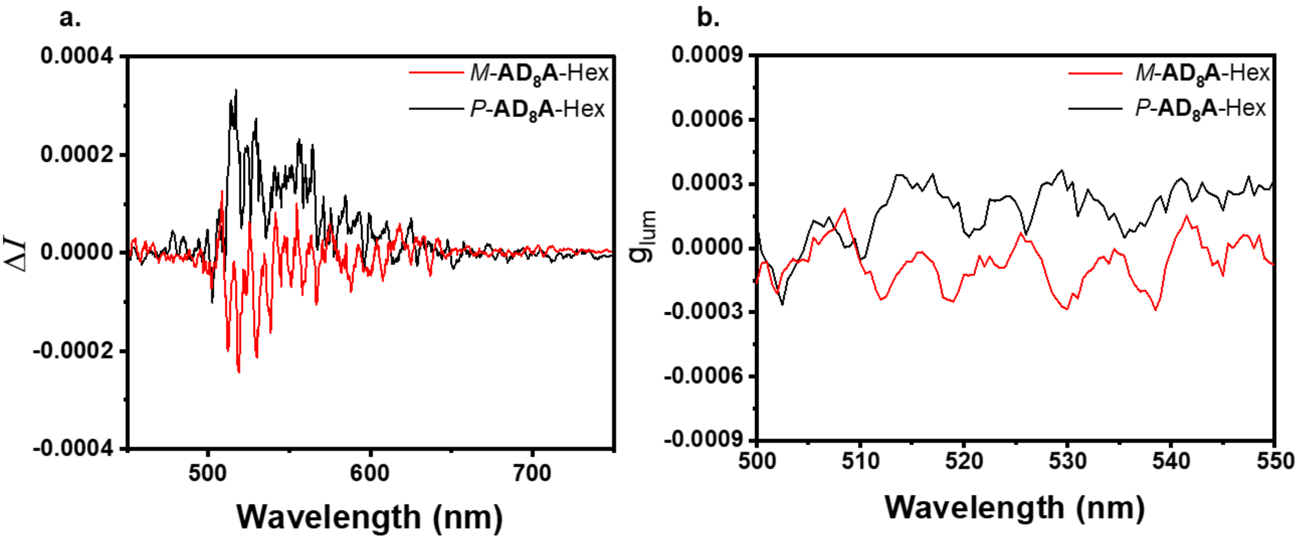

Figure S49. (a) CPL and (b) g_{lum} spectra of $\mathbf{AD_8A}$ enantiomers measured in hexane (Hex).

S4.8 Stationary vibrational spectroscopy

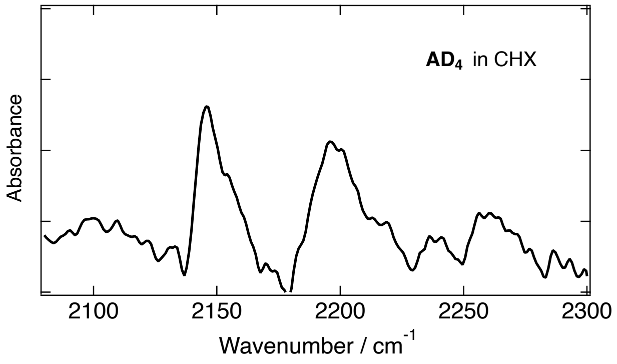

Figure S50. Stationary IR absorption spectra in the -C≡C- stretching region of **AD₄** in cyclohexane (CHX).

S5. Time-resolved spectroscopic measurements

S5.1 Electronic transient absorption spectroscopy

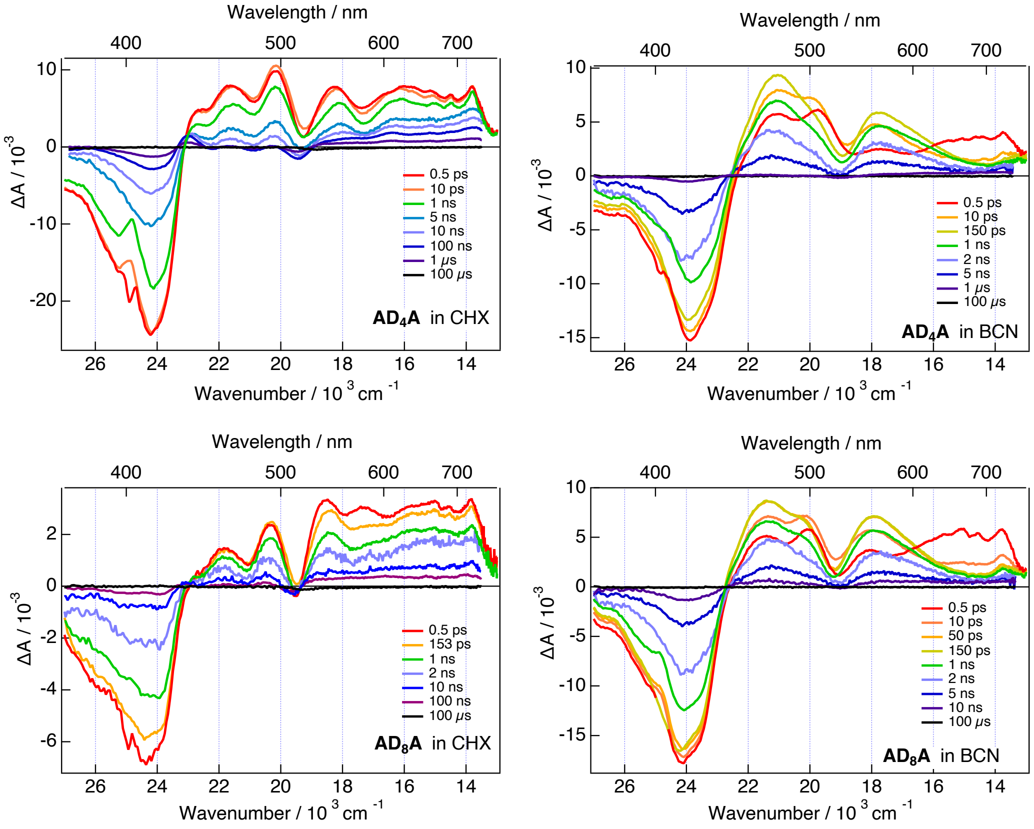

Figure S51. Transient absorption spectra recorded at different times after 400 nm excitation of $\mathbf{AD_nA}$ in cyclohexane (CHX) and benzonitrile (BCN).

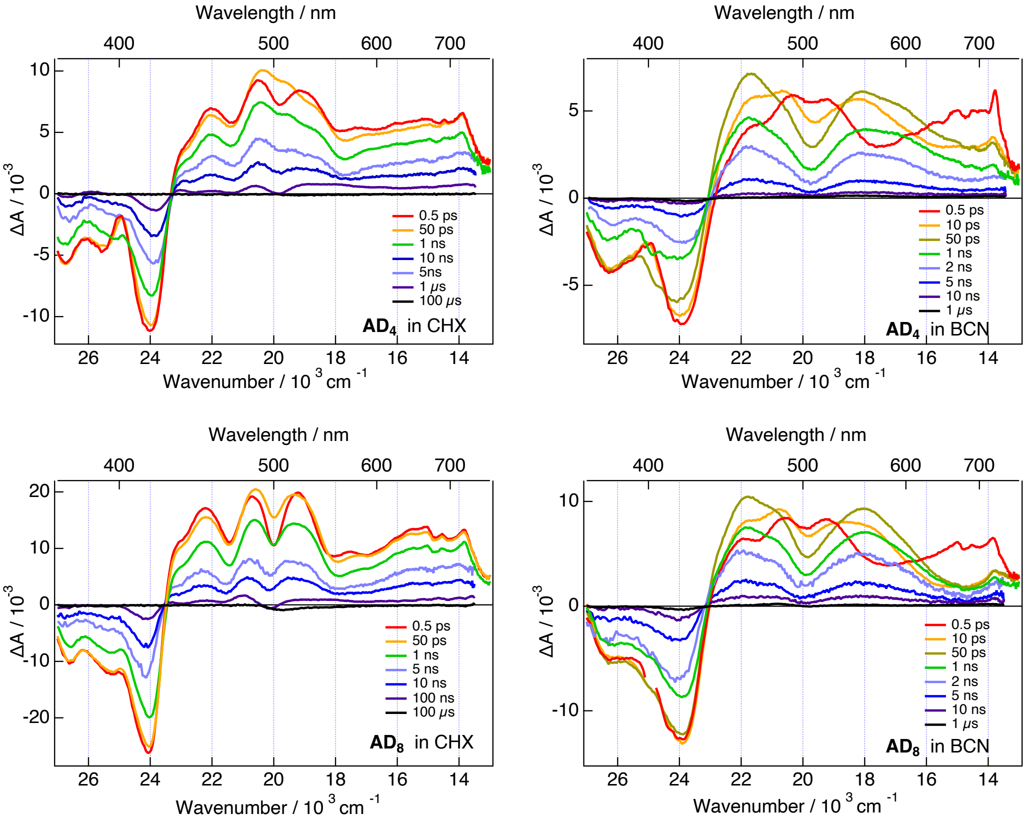

Figure S52. Transient absorption spectra recorded at different times after 400 nm excitation of $\mathbf{AD_n}$ in cyclohexane (CHX) and benzonitrile (BCN)

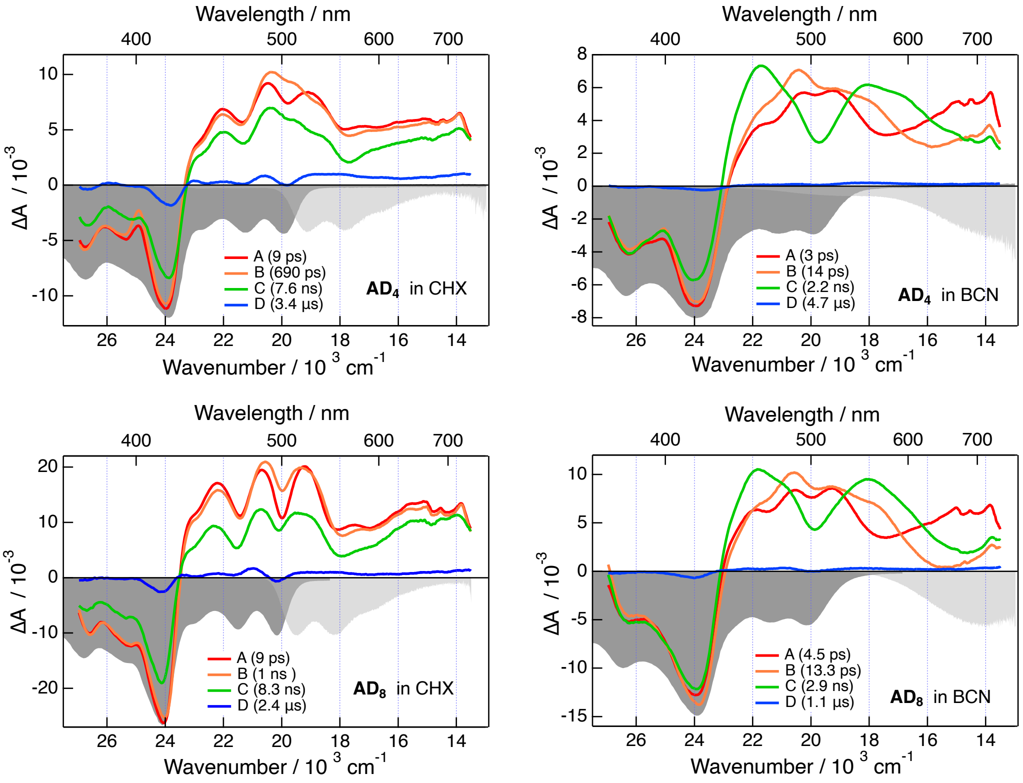

Figure S53. Evolution-associated difference absorption spectra and time constants obtained from a global analysis of the transient electronic absorption spectra recorded after 400nm excitation of **AD$_n$** in cyclohexane (CHX) and benzonitrile (BCN), assuming a series of four successive exponential steps.

S5.2 Time-resolved IR spectroscopy

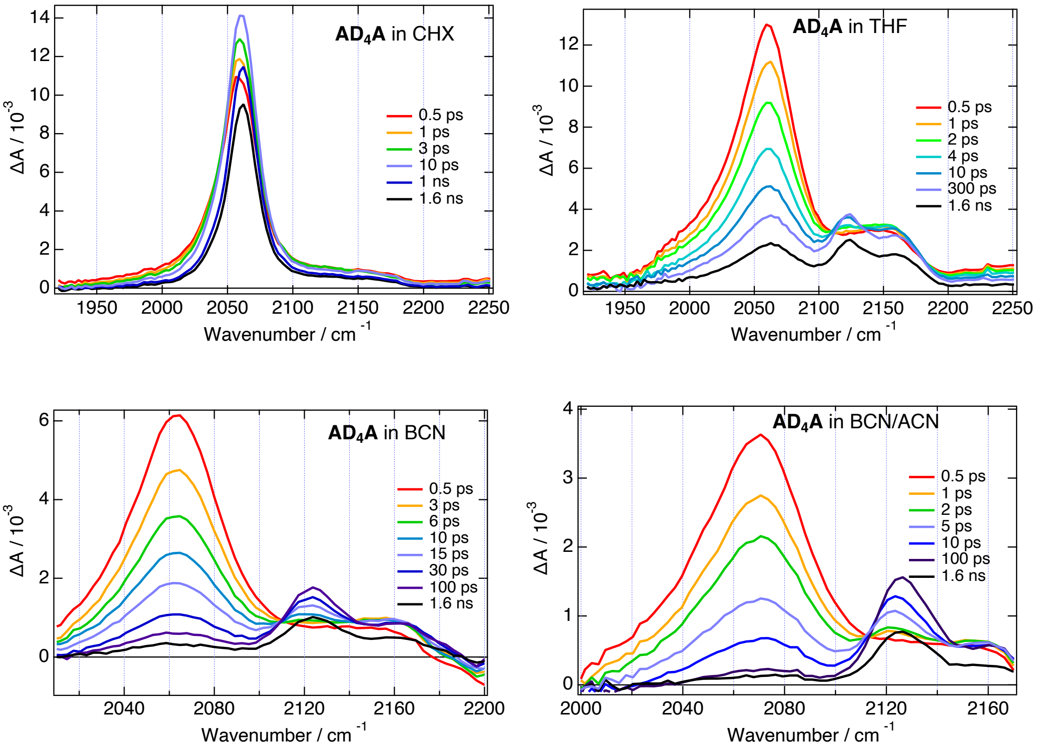

Figure S54. Time-resolved IR spectra recorded at different times after 400 nm excitation of **AD$_4$A** in various solvents.

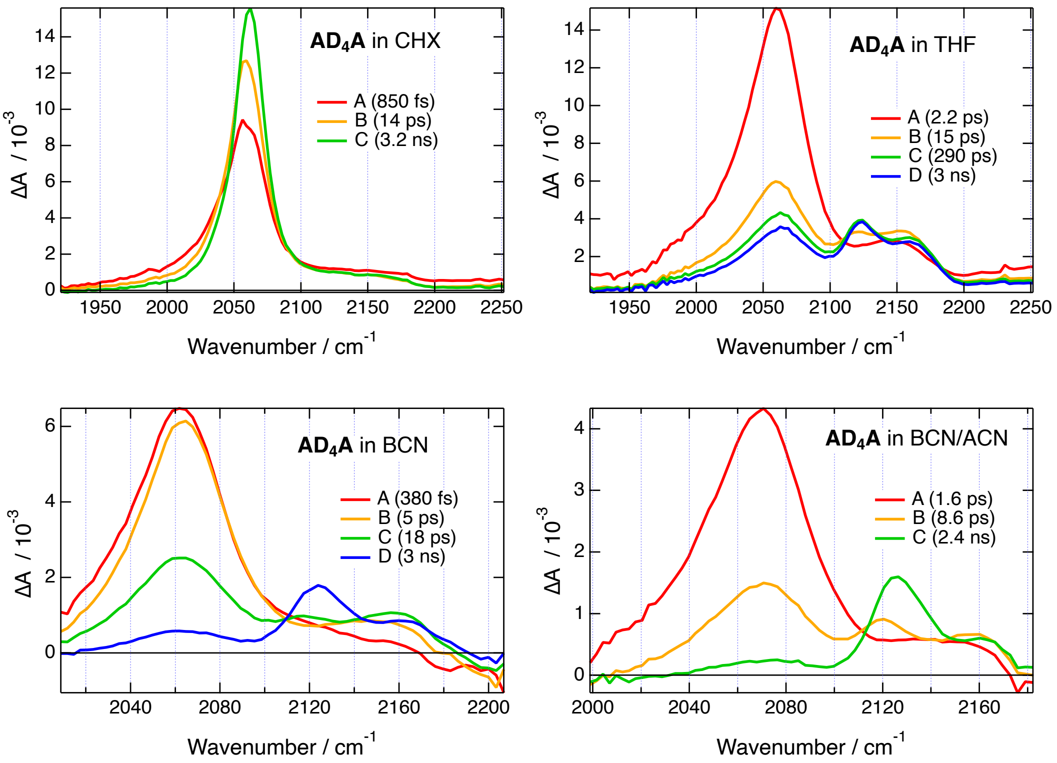

Figure S55. Evolution-associated difference absorption spectra and time constants obtained from a global analysis of the time-resolved IR spectra recorded after 400nm excitation of **AD4A** in various solvents assuming a series of successive steps.

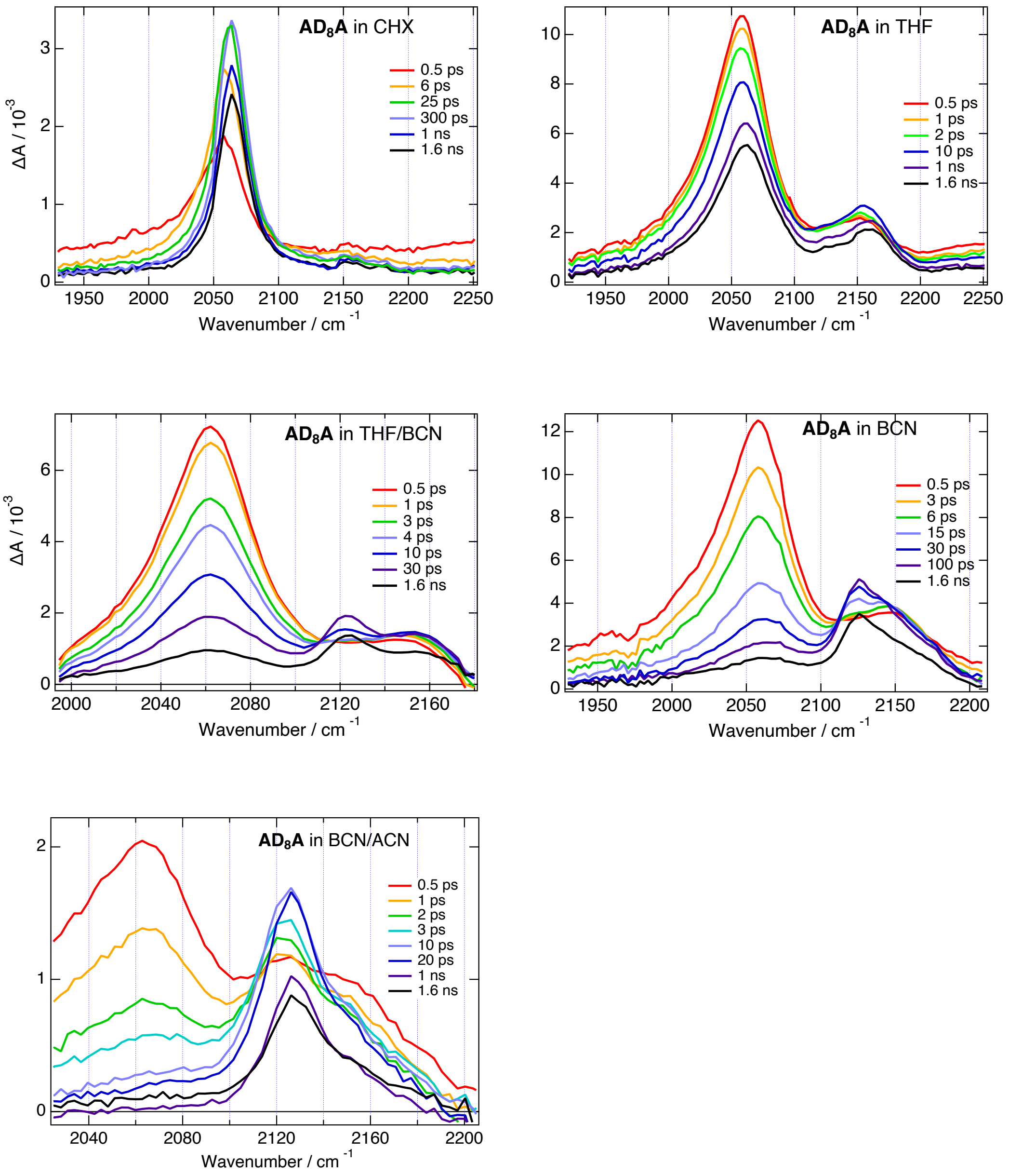

Figure S56. Time-resolved IR spectra recorded at different times after 400 nm excitation of **AD$_8$A** in various solvents: cyclohexane (CHX), THF and benzonitrile (BCN).

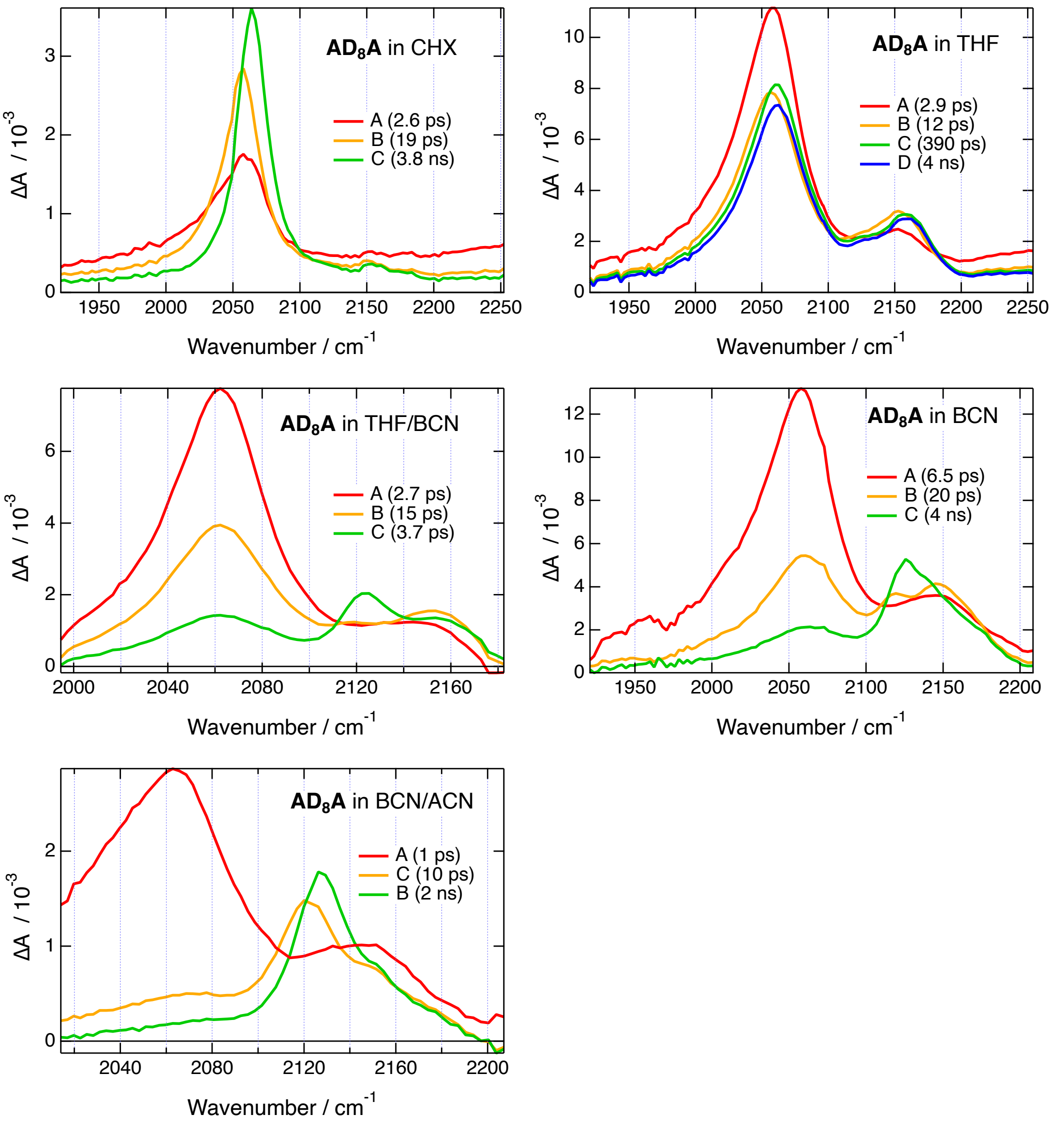

Figure S57. Evolution-associated difference absorption spectra and time constants obtained from a global analysis of the time-resolved IR spectra recorded after 400nm excitation of **AD$_8$A** in various solvents assuming a series of successive steps.

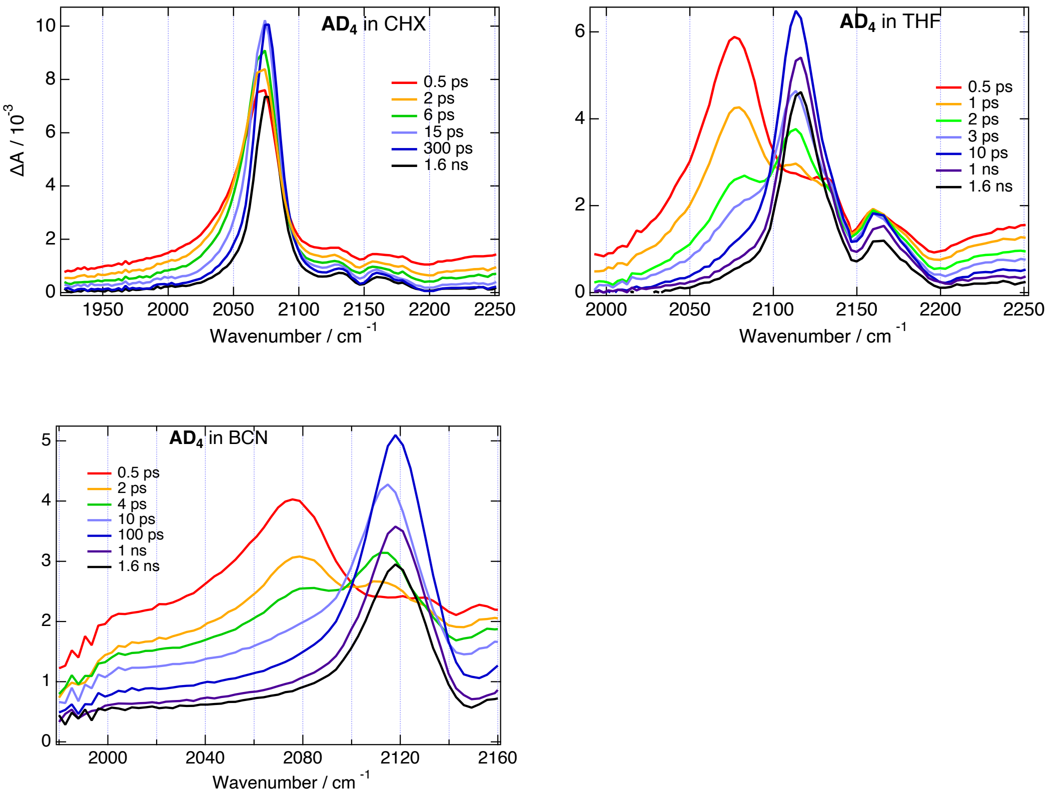

Figure S58. Time-resolved IR spectra recorded at different times after 400 nm excitation of $\mathbf{AD_4}$ in various solvents: cyclohexane (CHX), THF and benzonitrile (BCN).

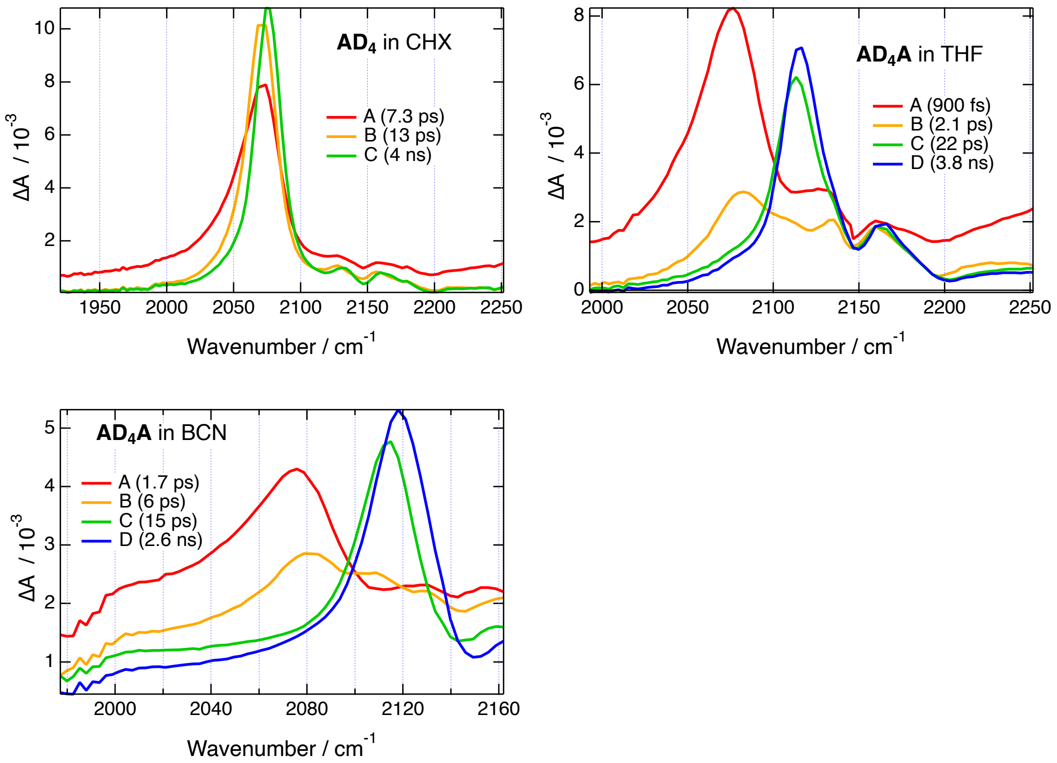

Figure S59. Evolution-associated difference absorption spectra and time constants obtained from a global analysis of the time-resolved IR spectra recorded after 400nm excitation of **AD_4** in various solvents assuming a series of successive steps.

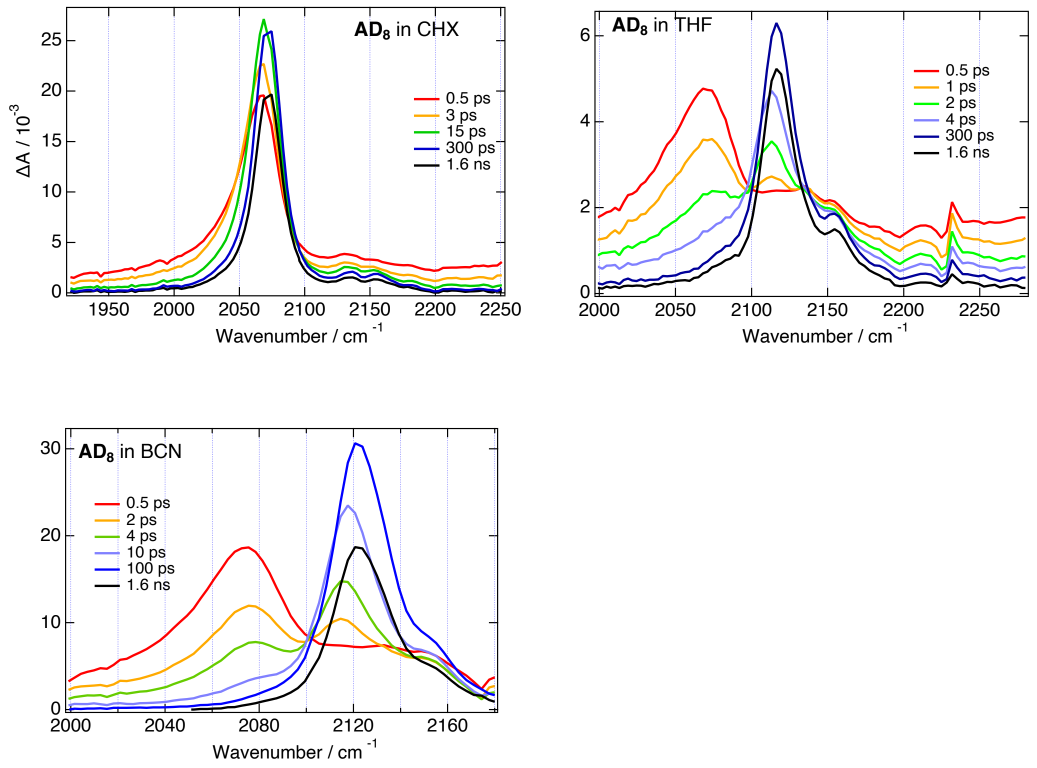

Figure S60. Time-resolved IR spectra recorded at different times after 400 nm excitation of **AD_8** in various solvents.

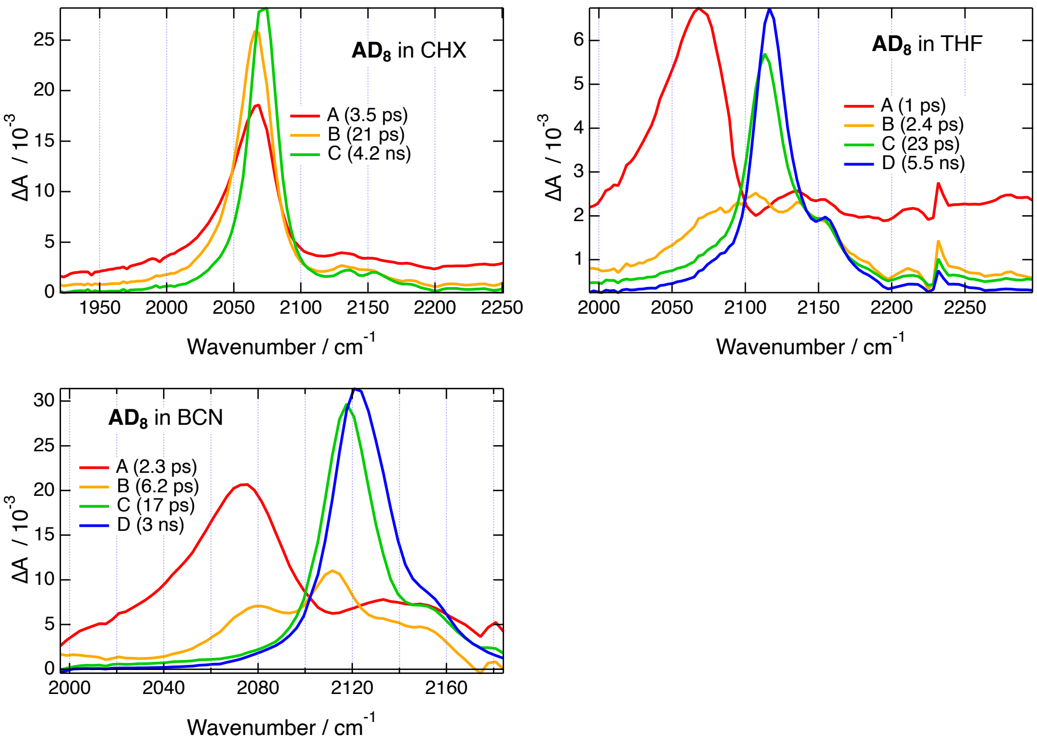

Figure S61. Evolution-associated difference absorption spectra and time constants obtained from a global analysis of the time-resolved IR spectra recorded after 400nm excitation of $\mathbf{AD_8}$ in various solvents assuming a series of successive steps.

S6 Single crystal X-ray diffraction crystallography (SCXRD)

Table 3. Crystal data and structure refinement for *rac*-**AD_4A**

**Identification code**	*Rac*-**AD_4A**
Deposition Number	2467522
Empirical formula	$C_{82}H_{74}N_2O_6$
Formula weight	1183.43
Temperature/K	150.0(1)
Crystal system	triclinic
Space group	P-1
a/Å	13.486(1)
b/Å	14.946(1)
c/Å	16.8069(6)
α/°	82.516(4)
β/°	81.466(5)
γ/°	74.694(7)
Volume/Å^3	3216.4(4)
Z	2
ρ_{calc}g/cm^3	1.222
μ/mm^{-1}	0.076
F(000)	1256.0
Crystal size/mm^3	0.4 × 0.07 × 0.04
Radiation	Mo Kα (λ = 0.71073)
2Θ range for data collection/°	4.226 to 49.994
Index ranges	$-16 \leq h \leq 15$, $-17 \leq k \leq 17$, $-18 \leq l \leq 19$
Reflections collected	26162
Independent reflections	11220 [R_{int} = 0.0707, R_{sigma} = 0.0926]
Data/restraints/parameters	11220/0/1000
Goodness-of-fit on F^2	1.070
Final R indexes [I>=2σ (I)]	R_1 = 0.1162, wR_2 = 0.3092
Final R indexes [all data]	R_1 = 0.1622, wR_2 = 0.3294
Largest diff. peak/hole / e Å^{-3}	0.58/-0.37

S7 Quantum-chemical calculations

All calculations were carried out using the Gaussian 09 program applying density functional theory (DFT).[6,7] All molecules were optimized using a hybrid density functional and Becke's three parameter exchange functional combined with the LYP correlation functional (B3LYP) and with the 6-31G(d) basis set (B3LYP/6-31G(d)).[8] To understand the UV-vis and CD spectral transition of the molecules, time dependent (TD)-DFT calculations were performed using the CAM-B3LYP functional with the 6-31G(d) basis set.[9] Scanning potential energy surface was performed using DFT/B3LYP-GD3/6-31G(d) (using Grimmes's dispersion with the D3 damping function).[10] Two-dimensional potential (2D) energy surface calculated at the Hartree–Fock level with the 6-31G(d) basis set, and the local and global minima were then optimized at DFT/B3LYP-GD3/6-31G(d) level of theory (Figure S63).

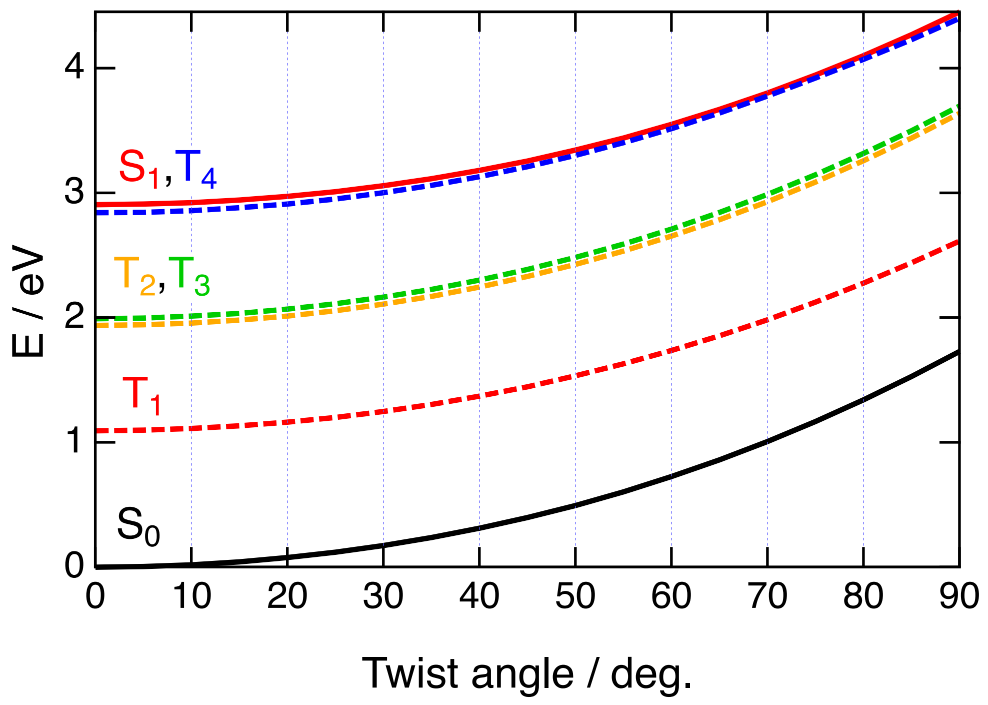

Figure S62. Energy of the ground and lowest Franck Condon singlet and triplet excited states of an analogue of **AD$_n$A** without the tether as a function of the twist angle calculated at the TD-DFT level (CAM-B3LYP/6-31G(d)) in the gas phase.

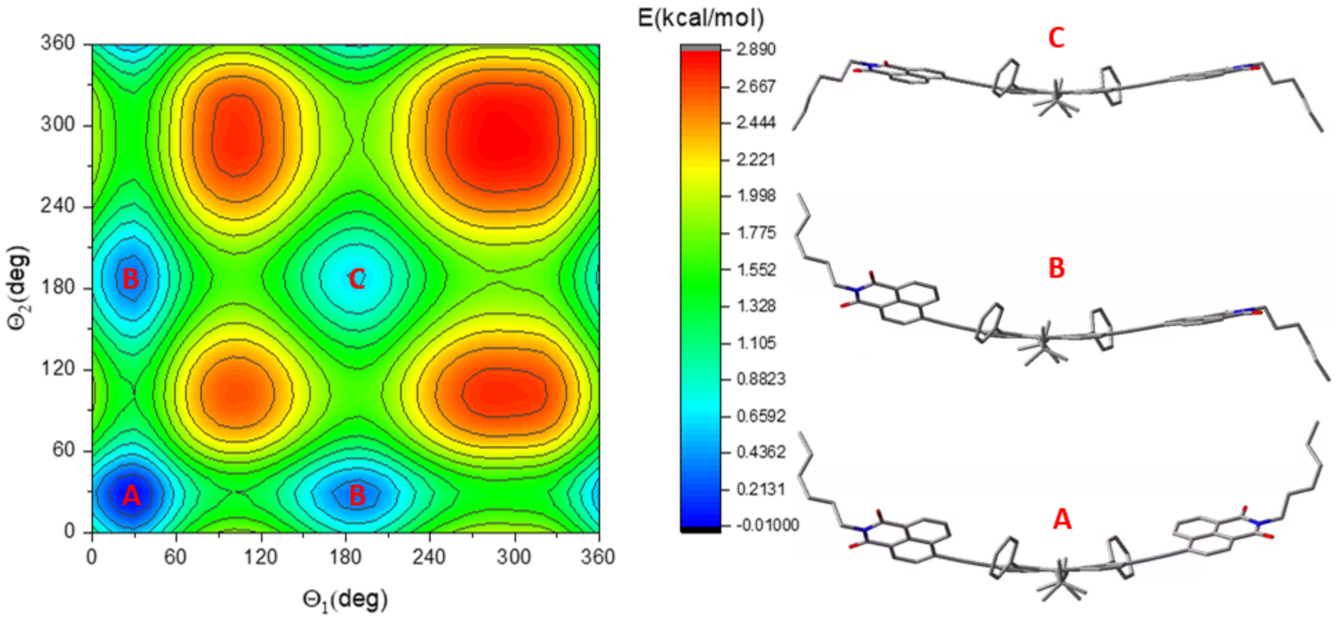

Figure S63. Two-dimensional potential energy surface generated by scanning two dihedral angles (θ_1 and θ_2) of **AD$_n$A** molecule. The plot shows three distinct conformers, due to the changes in these torsional degrees of freedom. These conformers were then optimized at the DFT/B3LYP-GD3/6-31G(d) level of theory.

S8 References

(1) Chuard, N.; Fujisawa, K.; Morelli, P.; Saarbach, J.; Winssinger, N.; Metrangolo, P.; Resnati, G.; Sakai, N.; Matile, S. Activation of Cell-Penetrating Peptides with Ionpair−π Interactions and Fluorophiles. *J. Am. Chem. Soc.* **2016**, *138* (35), 11264–11271. https://doi.org/10.1021/jacs.6b06253.

(2) Bedi, A.; Manor Armon, A.; Diskin-Posner, Y.; Bogosalvsky, B.; Gidron, O. Controlling the Helicity of π-Conjugated Oligomers by Tuning the Aromatic Backbone Twist. *Nat. Commun.* **2022**, *13* (1), 451. https://doi.org/10.1038/s41467-022-28072-7.

(3) Govind, C.; Balanikas, E.; Sanil, G.; Gryko, D. T.; Vauthey, E. Structural and Solvent Modulation of Symmetry-Breaking Charge-Transfer Pathways in Molecular Triads. *Chem. Sci.* **2024**, *15* (42), 17362–17371. https://doi.org/10.1039/D4SC05419A.

(4) Lang, B.; Mosquera-Vázquez, S.; Lovy, D.; Sherin, P.; Markovic, V.; Vauthey, E. Broadband Ultraviolet-Visible Transient Absorption Spectroscopy in the Nanosecond to Microsecond Time Domain with Sub-Nanosecond Time Resolution. *Rev. Sci. Instrum.* **2013**, *84* (7), 073107. https://doi.org/10.1063/1.4812705.

(5) Koch, M.; Letrun, R.; Vauthey, E. Exciplex Formation in Bimolecular Photoinduced Electron-Transfer Investigated by Ultrafast Time-Resolved Infrared Spectroscopy. *J. Am. Chem. Soc.* **2014**, *136* (10), 4066–4074. https://doi.org/10.1021/ja500812u.

(6) Calais, J.-L. Density-Functional Theory of Atoms and Molecules. R.G. Parr and W. Yang, Oxford University Press, New York, Oxford, 1989. IX + 333 Pp. Price £45.00. *Int. J. Quant. Chem.* **1993**, *47* (1), 101–101. https://doi.org/10.1002/qua.560470107.

(7) Frisch, M. J.; Trucks, G. W.; Schlegel, H. B.; Scuseria, G. E.; Robb, M. A.; Cheeseman, J. R.; Scalmani, G.; Barone, V.; Petersson, G. A.; Nakatsuji, H.; Li, X.; Caricato, M.; Marenich, A. V.; Bloino, J.; Janesko, B. G.; Gomperts, R.; Mennucci, B.; Hratchian, H. P.; Ortiz, J. V.; Izmaylov, A. F.; Sonnenberg, J. L.; Williams-Young, D.; Ding, F.; Lipparini, F.; Egidi, F.; Goings, J.; Peng, B.; Petrone, A.; Henderson, T.; Ranasinghe, D.; Zakrzewski, V. G.; Gao, J.; Rega, N.; Zheng, G.; Liang, W.; Hada, M.; Ehara, M.; Toyota, K.; Fukuda, R.; Hasegawa, J.; Ishida, M.; Nakajima, T.; Honda, Y.; Kitao, O.; Nakai, H.; Vreven, T.; Throssell, K.; Montgomery, J. A. Jr.; Peralta, J. E.; Ogliaro, F.; Bearpark, M. J.; Heyd, J. J.; Brothers, E. N.; Kudin, K. N.; Staroverov, V. N.; Keith, T. A.; Kobayashi, R.; Normand, J.; Raghavachari, K.; Rendell, A. P.; Burant, J. C.; Iyengar, S. S.; Tomasi, J.; Cossi, M.; Millam, J. M.; Klene, M.; Adamo, C.; Cammi, R.; Ochterski, J. W.; Martin, R. L.; Morokuma, K.; Farkas, O.; Foresman, J. B.; Fox, D. J. Gaussian 16 Revision C.01, 2016.

(8) Lee, C.; Yang, W.; Parr, R. G. Development of the Colle-Salvetti Correlation-Energy Formula into a Functional of the Electron Density. *Phys. Rev. B* **1988**, *37* (2), 785–789. https://doi.org/10.1103/PhysRevB.37.785.

(9) Yanai, T.; Tew, D. P.; Handy, N. C. A New Hybrid Exchange–Correlation Functional Using the Coulomb-Attenuating Method (CAM-B3LYP). *Chem. Phy. Lett.* **2004**, *393* (1), 51–57. https://doi.org/10.1016/j.cplett.2004.06.011.

(10) Becke, A. D. Density-functional Thermochemistry. III. The Role of Exact Exchange. *J. Chem. Phys.* **1993**, *98* (7), 5648–5652. https://doi.org/10.1063/1.464913.
